# A Noncovalent Click‐to‐Release Strategy to Control Bond Cleavage and Prodrug Activation

**DOI:** 10.1002/anie.202515594

**Published:** 2026-01-18

**Authors:** Xuancheng Fu, Bowen Xu, Suman Maity, Michelle Wu, Luke G. Westbrook, James H. Henderson, Yaoying Wu, Katie A. Edwards, Atanu Acharya, Xiaoran Hu

**Affiliations:** ^1^ Department of Chemistry Syracuse University Syracuse New York 13244 USA; ^2^ BioInspired Institute Syracuse University Syracuse New York 13244 USA; ^3^ Department of Biomedical & Chemical Engineering Syracuse University Syracuse New York 13244 USA; ^4^ Department of Microbiology and Immunology State University of New York, Upstate Medical University Syracuse New York 13210 USA; ^5^ Department of Pharmaceutical Sciences School of Pharmacy and Pharmaceutical Sciences Binghamton University Binghamton New York 13902 USA

**Keywords:** Bioorthogonal cleavage, Click to release, Host‐guest systems, Noncovalent click chemistry, Self–immolative chemistry

## Abstract

Click‐to‐release chemistry enables bioorthogonal bond cleavage and controlled release via a click‐type ligation reaction serving as both the trigger and means of localization. Extending this concept beyond covalent ligation reactions, we introduce a noncovalent click‐to‐release strategy based on cucurbit[7]uril‐adamantane (CB‐Ad) association. The CB host molecule forms a pre‐assembled host‐guest complex with a self‐immolative guest (SIG) SIG1, where the masked SIG remains inert. Introduction of a high‐affinity guest Ad initiates the CB‐Ad noncovalent click reaction, displacing SIG1 and triggering its self‐immolation and cargo release. As a proof‐of‐concept, we used a prototype prodrug SIG2 to demonstrate our strategy's potential for controlled therapeutic release, effectively regulating the photodynamic cell killing in vitro. This noncovalent click‐to‐release approach broadens the structural and functional scope of bioorthogonal cleavage strategies with promising implications for stimuli‐responsive materials and biomedical applications.

## Graphical abstract

Extending the click‐to‐release concept beyond covalent ligation, we introduce a noncovalent click‐to‐release strategy based on cucurbituril‐adamantane (CB‐Ad) association. A supramolecular preassembly encapsulating a self‐immolative guest (SIG) is rationally designed to prevent premature release, while adding a high‐affinity Ad guest initiates the CB‐Ad click reaction, displacing the SIG and triggering its preprogrammed cleavage for cargo release.

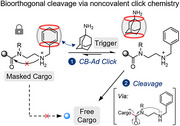

“Click to release” is a type of bioorthogonal cleavage reaction in which a selective, click‐type ligation reaction, such as inverse electron‐demand Diels‐Alder cycloaddition and Staudinger ligation, initiates a cascade that results in bond cleavage and payload release (Figure 1a).^[^ ^1^, ^2^, ^3^, ^4^, ^5^ ^]^ Because the ligation event is both the trigger and means of localization, click‐to‐release strategies offer spatial and temporal control over the cleavage events, making them invaluable tools for various chemical biology and therapeutic applications. For example, scientists have developed drug delivery strategies that locally capture *trans*‐cyclooctene (TCO)‐modified prodrugs and release native cytotoxic agents using tetrazine (Tz) linked to a targeting motif,^[^ ^6^, ^7^ ^]^ immobilized on cell surfaces,^[^ ^8^ ^]^ or incorporated into a hydrogel implant.^[^ ^9^, ^10^ ^]^ Click‐to‐release has also enabled bioorthogonal probe activation^[^ ^11^, ^12^, ^13^, ^14^ ^]^ and on‐demand biological perturbations.^[^ ^15^, ^16^, ^17^ ^]^ Despite the success of existing strategies, conventional click‐to‐release reactions are often limited by specific drawbacks, such as oxidation susceptibility in Staudinger reactions, slow reaction kinetics for azide–cyclooctene systems, and side reactions limiting TCO‐Tz release efficacy.^[^ ^5^, ^18^ ^]^ Thus, the development of diverse, robust click‐to‐release strategies remains a critical task for broader biological applications.

**Figure 1:**
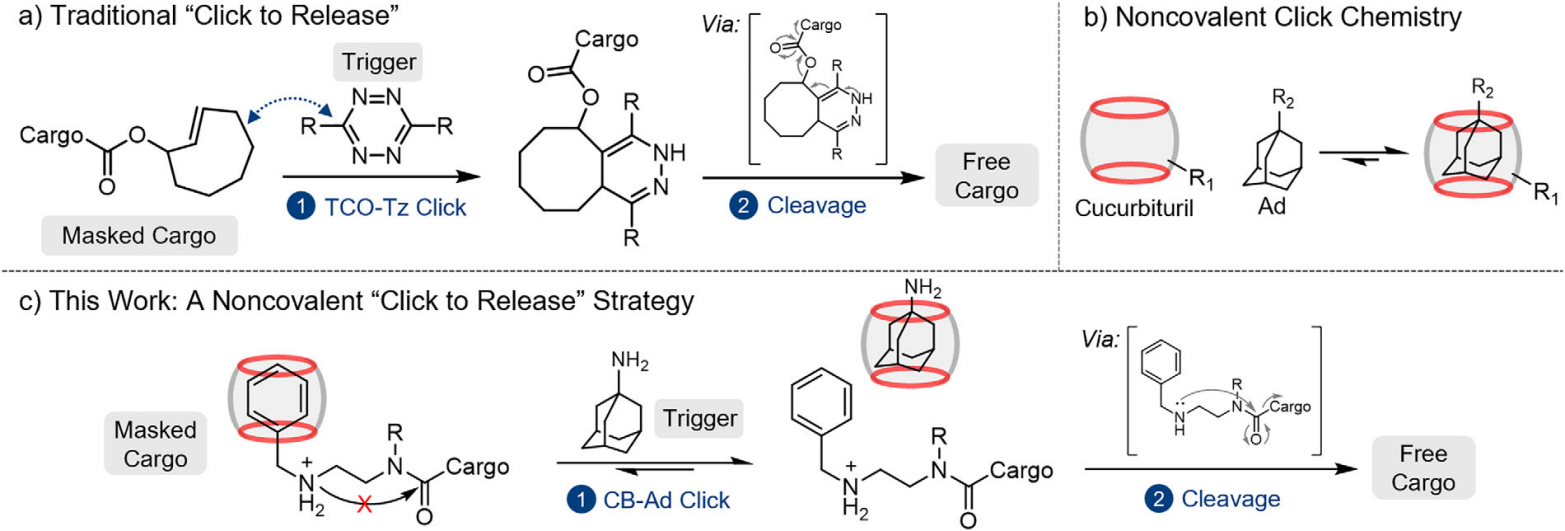
Overview. a) Traditional click‐to‐release strategies employ covalent click reactions to trigger cascade bond cleavage. b) High‐affinity cucurbituril‐adamantane host‐guest interactions are widely used as noncovalent click chemistry. c) Our noncovalent click‐to‐release strategy harnesses CB‐Ad binding to control covalent bond cleavage and cargo release.

The emerging concept of noncovalent click reactions^[^ ^19^, ^20^, ^21^, ^22^, ^23^, ^24^, ^25^ ^]^ (Figure 1b) also known as supramolecular latching^[^ ^26^, ^27^ ^]^ offers a compelling complement to traditional covalent ligation approaches, with the potential to significantly broaden click chemistry's structural and functional scope. Such noncovalent strategies exploit complementary molecular recognition partners to form ultra‐stable, yet reversible host‐guest complexes. Of particular interest are host‐guest binding mediated by cucurbiturils, a group of synthetic macrocyclic molecules that bind complementary guests with *K*_a_ values up to 10^9^–10^15^ M^−1^.^[^ ^28^, ^29^, ^30^, ^31^ ^]^ These noncovalent associations occur without interfering with biological systems and have been widely featured as noncovalent click or bioorthogonal chemistry for applications such as biomolecule labeling^[^ ^32^, ^33^, ^34^, ^35^, ^36^ ^]^ and biological imaging.^[^ ^37^, ^38^, ^39^, ^40^, ^41^ ^]^ Noncovalent click reactions differ fundamentally from conventional covalent ligation by relying on high‐affinity molecular recognition rather than highly activated reagents, offering distinct structure‐property relationships that may open new avenues for designing and diversifying click systems. Noncovalent systems also exhibit distinct features such as (typically) rapid binding kinetics and reversible ligation. Extending the noncovalent click chemistry to click‐to‐release systems could therefore significantly diversify and enhance the structural and functional scope of bioorthogonal cleavage strategies.

Unlike click‐to‐release chemistry which releases covalently linked cargo molecules through bond cleavage, a related yet fundamentally different strategy uses dynamic host–guest interactions to control the release of physically entrapped cargos without bond‐breaking.^[^ ^42^ ^]^ In such systems, bioactive molecules are sequestered by the guest and deactivated, while introducing a high‐affinity competing guest uncages the bioactive cargo.^[^ ^43^, ^44^, ^45^, ^46^, ^47^, ^48^, ^49^, ^50^ ^]^ However, this physical entrapment strategy is constrained to specific payloads that simultaneously exhibit potent bioactivity and high host‐guest binding affinity—an uncommon combination that significantly restricts broader applicability. Thus, chemical modification is typically required to enhance host‐guest interactions, but such modifications often compromise or abolish the intended function of the payload. A general approach that enables the supramolecular controlled release of unmodified payload molecules remains an undeveloped yet transformative research avenue.

Herein, we introduce a noncovalent click‐to‐release strategy to control bioorthogonal cleavage and prodrug activation through cucurbit[7]uril‐mediated host‐guest association (Figure 1c). We rationally designed a self‐immolative guest (SIG) model molecule **SIG1** to form a pre‐assembled complex with the CB host molecule. The self‐immolative reactivity of CB‐capped **SIG1**—referred to as CB/**SIG1**, where CB essentially serves as a “supramolecular protecting group” masking the reactive functional group is effectively suppressed in the pre‐assembly, preventing premature cargo release. The subsequent introduction of a high‐affinity 1‐adamantylamine (Ad) guest molecule initiates an association‐based competition, which functions as a noncovalent click chemistry with CB to displace **SIG1**, triggering its preprogrammed cleavage and cargo release. As a proof‐of‐concept, we developed a prototype prodrug **SIG2** to demonstrate the potential of this platform in the controlled release of a therapeutic photosensitizer, effectively regulating the photodynamic killing of *HeLa* cells. By extending the concept of noncovalent click chemistry to click‐to‐release systems, our platform broadens the structural and functional scope of bioorthogonal cleavage strategies with promising implications for material science and biomedical applications.

The CB mediated host–guest interaction, a classical noncovalent click chemistry system^[^ ^20^ ^]^ with high binding affinity and selectivity, was chosen for this initial study. Benzylamines exhibit high binding affinities (∼10^5^–10^7^ M^−1^)^[^ ^28^ ^]^ with CB, where the ammonium cation remains at the negatively polarized CB portal, while the benzyl motif displaces the high‐energy water inside the hydrophobic CB cavity.^[^ ^51^, ^52^, ^53^ ^]^ Inspired by ethylenediamine‐carbamate self‐immolative spacer structures that undergo cleavage via intramolecular cyclization,^[^ ^54^, ^55^, ^56^, ^57^, ^58^ ^]^ we designed and synthesized SIG molecules containing a CB‐binding benzylamine motif (Figure 2a), with the cyclization‐elimination reactivity of a model SIG molecule confirmed by isolating and analyzing its self‐immolation products (Figure S1). The cyclization‐elimination proceeds via nucleophilic attack of the secondary amine on the carbonyl group (Figure 1c). We hypothesized that caging SIG's benzylamine motif by CB would introduce steric hindrance around the nucleophilic reactive site, thereby suppressing the amine and carbonyl sites from approaching each other and inhibiting the cyclization‐elimination. Although preliminary studies with a model compound **SIG0** showed noticeable reactivity suppression (Figure S2), the degree of inhibition was insufficient. To further amplify this steric congestion effect, we rationally designed **SIG1** (Figure 2) incorporating an additional steric substituent at the carbamate nitrogen—a benzyl substituent was chosen for synthetic feasibility. Computational results revealed the geometry of the CB‐SIG complexes, where the benzylamine guest motifs were fully inserted inside the bulky CB cages (Figure 2b).

**Figure 2:**
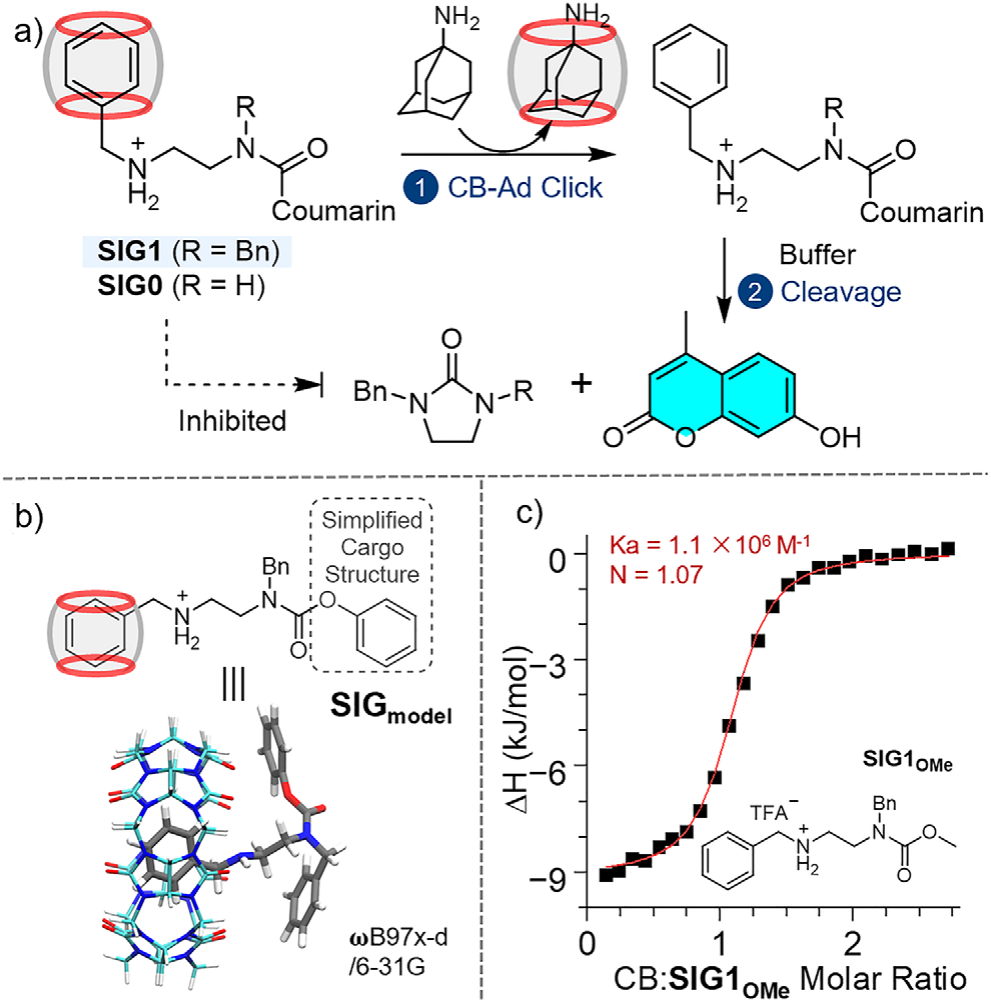
a) Schematic showing that a CB‐**SIG1** pre‐assembly is rationally designed to mask the reactivity, while CB‐Ad click reaction activates the self‐immolative structure, initiating the cargo release. The structure of **SIG0** is also given. b) Optimized structure of a model host‐guest complex. Atoms: oxygen (red), nitrogen (blue), hydrogen (white); CB and **SIG_model_** carbons in cyan and grey, respectively. c) ITC titration of **SIG1_OMe_** with CB in MES buffer.

Experimentally, we first validated the host‐guest binding between CB and **SIG1_OMe_**, an inactive **SIG1** analogue in which methanol acts as a poor leaving group (Figure S3a), making it suitable for investigating the SIG platform under buffered conditions where **SIG1** would otherwise undergo self‐immolation. Isothermal titration calorimetry (ITC) measurements (Figure 2c) reveal a 1:1 binding between CB and **SIG1_OMe_** with high affinity (*K*_a_ = 1.1 × 10^6^ M^−1^) in pH 6.5 MES buffer. Additionally, NMR results (Figures S9–S11) confirm selective CB binding to the benzylamine motif. Although **SIG1** could not be directly measured under buffered conditions due to its intrinsic reactivity, its binding behavior in pure water was comparable to that of **SIG1_OMe_** (Supporting Information Section 3: Binding Studies).

To identify pre‐assembly formulations that effectively prevent premature release, we next investigated the reactivity of CB‐**SIG1** complexes as a function of CB equivalents. Complexes containing 10 µM **SIG1** and varying equivalents of CB were incubated in MES buffer, and their conversion was monitored by tracking the release of the fluorogenic coumarin cargo using fluorescence spectroscopy. The half‐life (t_1/2_) of free **SIG1** was estimated to be 1.5 h by fitting the time‐dependent coumarin release results to a first‐order rate expression (Figure 3a). The introduction of 1/2/4/10 × CB extended the t_1/2_ of coumarin release to 3.5/12.4/32.3/82.8 h respectively, representing up to 55‐fold slowdown compared to the free **SIG1**. The residual release is attributed to incomplete host‐guest complexation (Supporting Information Section 4: Equilibrium Calculations). Additionally, studies conducted at millimolar concentrations enabled monitoring **SIG1** self‐immolation by NMR, confirming binding‐induced suppression of reactivity (Figure S4). Based on these results, the 10×CB/10 µM **SIG1** formulation provides a stable pre‐assembly that strongly suppresses release at biologically relevant concentrations.

**Figure 3:**
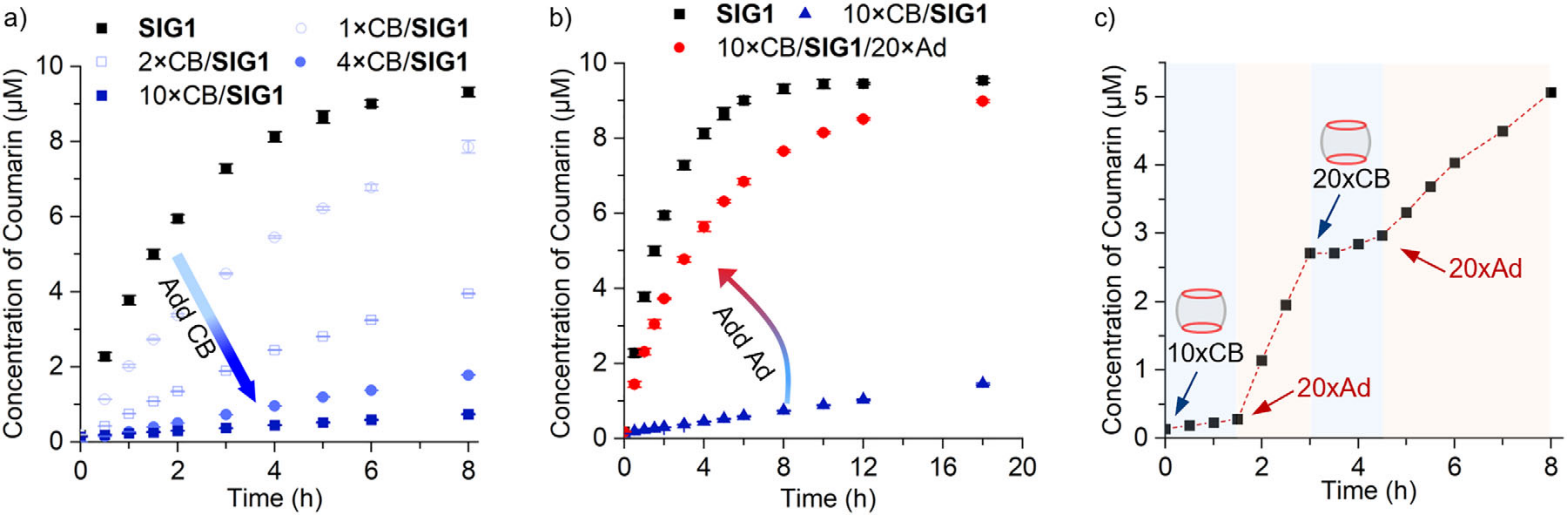
Concentration of released coumarin from 10 µM **SIG1** pre‐assembled with varying equivalents of CB, with or without added Ad: a) pre‐assemblies with increasing CB equivalents exhibit slower coumarin release, while b) introducing Ad restores the release rate (red dot). c) Ad‐triggered release from the 10×CB/**SIG1** complex can be reversibly controlled: release is inhibited in the pre‐assembled complex form, activated by 20 × Ad at 1.5 h, paused by the addition of 20 × CB at 3 h, and reactivated by another 20 × Ad at 4.5 h. *λ*_ex_ = 365 nm. Experiments were conducted in pH 6.5 MES solutions at room temperature. Error bars: standard deviation from three repeats.

Cleavage and cargo release from the masked CB‐**SIG1** pre‐assembly can be reactivated by adding the “supramolecular click trigger” Ad (Figure 3b), leveraging Ad's ultra‐high affinity as a competing guest and its biocompatibility as an FDA‐approved drug.^[^ ^59^ ^]^ We added 200 µM Ad into the 10×CB/10 µM **SIG1** complex. The introduction of Ad essentially restored the rate of coumarin release as shown by fluorescence measurements (Figure 3b). Further, the dynamic nature of the CB‐Ad click reaction allows us to switch the cleavage activity on and off reversibly (Figure 3c): alternating addition of CB and Ad turned the self‐immolation off and on, respectively, enabling temporal control of the guest reactivity on demand. Importantly, we effectively regulated the on‐off switching of **SIG1** reactivity in MES buffer supplemented with 1% serum or 1 mg/mL cell lysate (Figure S5), indicating negligible competing interactions with these biomaterials and consistent with the well‐established bioorthogonal nature of cucurbituril host‐guest interactions. We also evaluated the cell permeability of the SIG system under different supramolecular environments using a fluorescent model molecule **SIG1_NBD_** (Figure S6): **SIG1_NBD_** exhibited efficient cellular uptake, while CB complexation impedes **SIG1_NBD_** uptake likely by masking its cationic sites. Addition of Ad partially restored cellular uptake. The Ad‐triggered cellular uptake, together with controlled cleavage and release, provide a synergistic advantage for potential application as a controlled‐release platform.

Next, we examined the generality of this noncovalent click‐to‐release strategy (Figure 4a). Compound **SIG1_SCO_**, **SIG1_4NP_**, and **SIG1_4‐OHT_** bearing different phenolic cargos exhibited similar behaviors to **SIG1** (Figure S7 and S8), suggesting that our strategy applies broadly to phenols. SIGs with acholic and amine leaving groups showed negligible self‐immolative reactivity (Figure S3), reflecting a reduced rate of the rate‐determining cyclization‐elimination step^[^ ^57^ ^]^ due to their poor leaving‐group ability.^[^ ^60^, ^61^ ^]^ To overcome this limitation in cargo scope, a classical 4‐hydroxybenzyl alcohol linker^[^ ^62^, ^63^, ^64^ ^]^ (Figure 4b) was incorporated in the prototype prodrug **SIG2** to enable the release of an otherwise unreactive amine cargo, reduced methylene blue (rMB), via a rapid 1,6‐elimination pathway.^[^ ^65^ ^]^ rMB is then instantaneously oxidized by dissolved oxygen to methylene blue (MB),^[^ ^66^ ^]^ a well‐known photosensitizer used in photodynamic therapy (PDT).^[^ ^67^ ^]^

**Figure 4:**
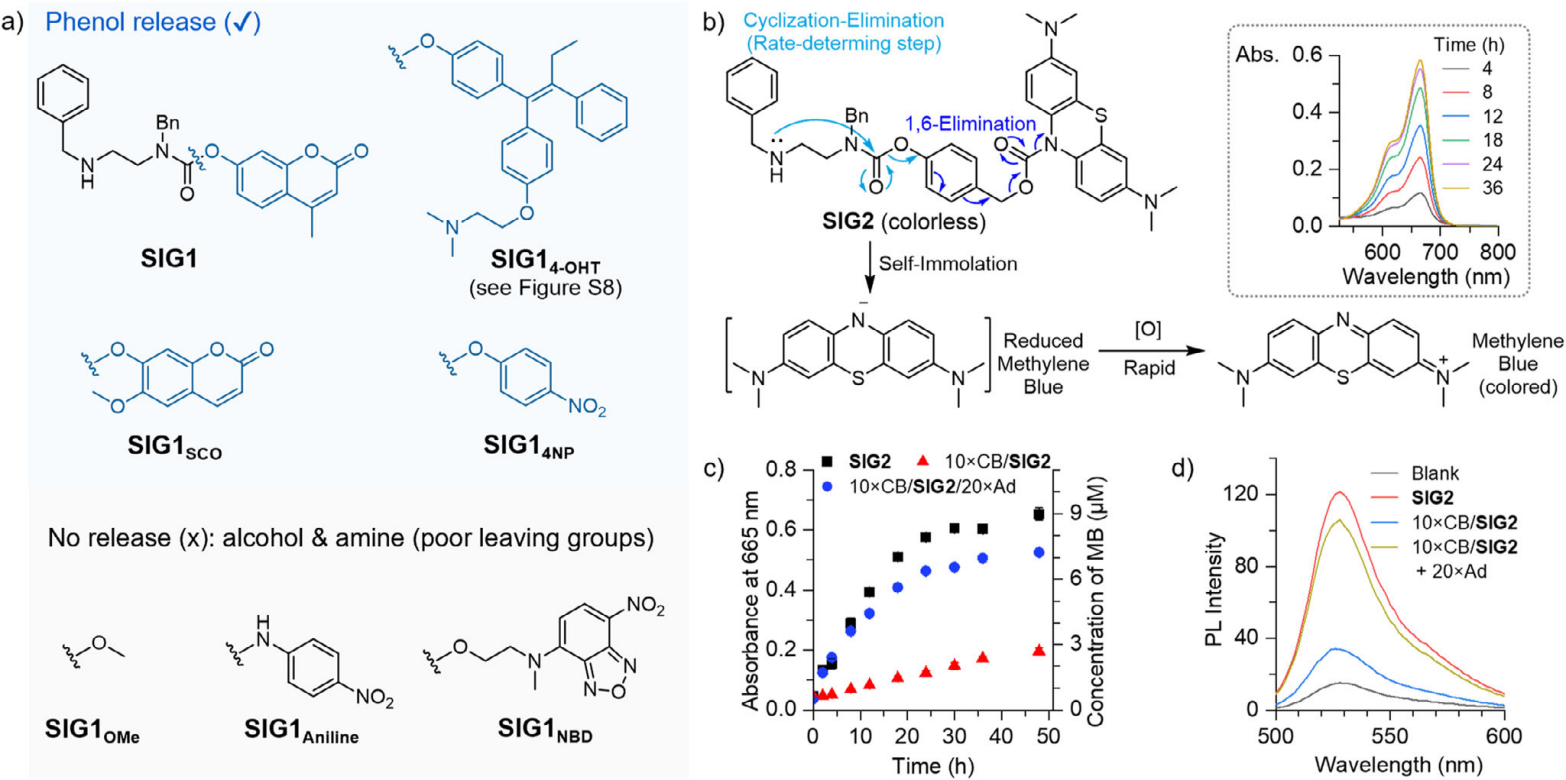
a) Structural scope of examined substrates. Also see Figure S8 for an in vitro demonstration of **SIG1_4‐OHT_** prodrug activation. b) Structure and reactivity of **SIG2**. Inset: Time‐course absorbance of a **SIG2** solution. c) Peak absorbance (left y‐axis) and corresponding MB concentration (right y‐axis) of respective solutions over time. See Figure S15 for details. Error bars: standard deviation from three repeats. d) Photo‐generated ^1^O_2_ in respective SIG systems analyzed post 4‐h incubation using SOSG assay ([SOSG] = 5 µM, λ_excitation_ = 488 nm). All incubations were conducted with 16 µM **SIG2** in pH 6.5 MES solutions with respective stoichiometries of CB and Ad at 37 °C. Light irradiation: 5 min, > 560 nm, 2.2 mW/cm^2^.

Upon dissolving **SIG2** in MES buffer, its self‐immolative reactivity was evidenced by the emerging MB absorption peak at 665 nm (Figure 4b) and was further confirmed through isolation of reaction products (Figure S14). Pre‐assembly with 10 × CB suppressed the rate of cleavage and release (Figure 4c, triangle). Importantly, subsequent addition of 20 × Ad displaced **SIG2** through competing CB–Ad binding, acting as a noncovalent click reaction to restore self‐immolative reactivity and MB release (Figure 4c, dot). Singlet Oxygen Sensor Green (SOSG) was used to evaluate the photoinduced ^1^O_2_‐generation ability of the released MB (Figure 4d, also see Supporting Information Section 5). Significant SOSG fluorescence was observed in the free **SIG2** group (Figure 4d, red), while fluorescence was suppressed in the pre‐assembled 10×CB/**SIG2** group (blue). Notably, the 10×CB/**SIG2**/20×Ad group (brown) showed restored fluorescence, demonstrating that the CB‐Ad click reaction effectively triggered MB release and activated its photodynamic effect. We attribute the less pronounced rate suppression for **SIG2**, compared to that in **SIG1** (Figure 3), to the higher temperature (37 °C) used in **SIG2** experiments, which weakened binding affinity and led to incomplete complexation. Future work will enhance binding by incorporating higher‐affinity motifs into the SIG platform.

The prototype **SIG2** prodrug serves as a proof‐of‐concept and demonstrates noncovalent “click‐to‐release” control of PDT cell killing (Figure 5a and Supporting Information: PDT Experiments). *HeLa* cells incubated with free **SIG2** followed by 10 min photo‐irradiation showed < 10% viability (Figure 5b, solid). In contrast, PDT treatment using the pre‐assembled 10×CB/**SIG2** complex under otherwise identical conditions exhibited minimal photo‐induced cytotoxicity (Figure 5b, striped). Importantly, the PDT effect was restored in the 10×CB/**SIG2**/20×Ad group (Figure 5b, crosshatched), which included Ad as a noncovalent click chemistry triggering component that initiated prodrug activation. Control experiments in the dark showed good viability across all groups (Figure 5b, white bars), confirming that the observed therapeutic cytotoxic effects arise from PDT. Treatments involving only supramolecular entities (CB, Ad, or both) demonstrated no cytotoxicity (Figure 5c).

**Figure 5:**
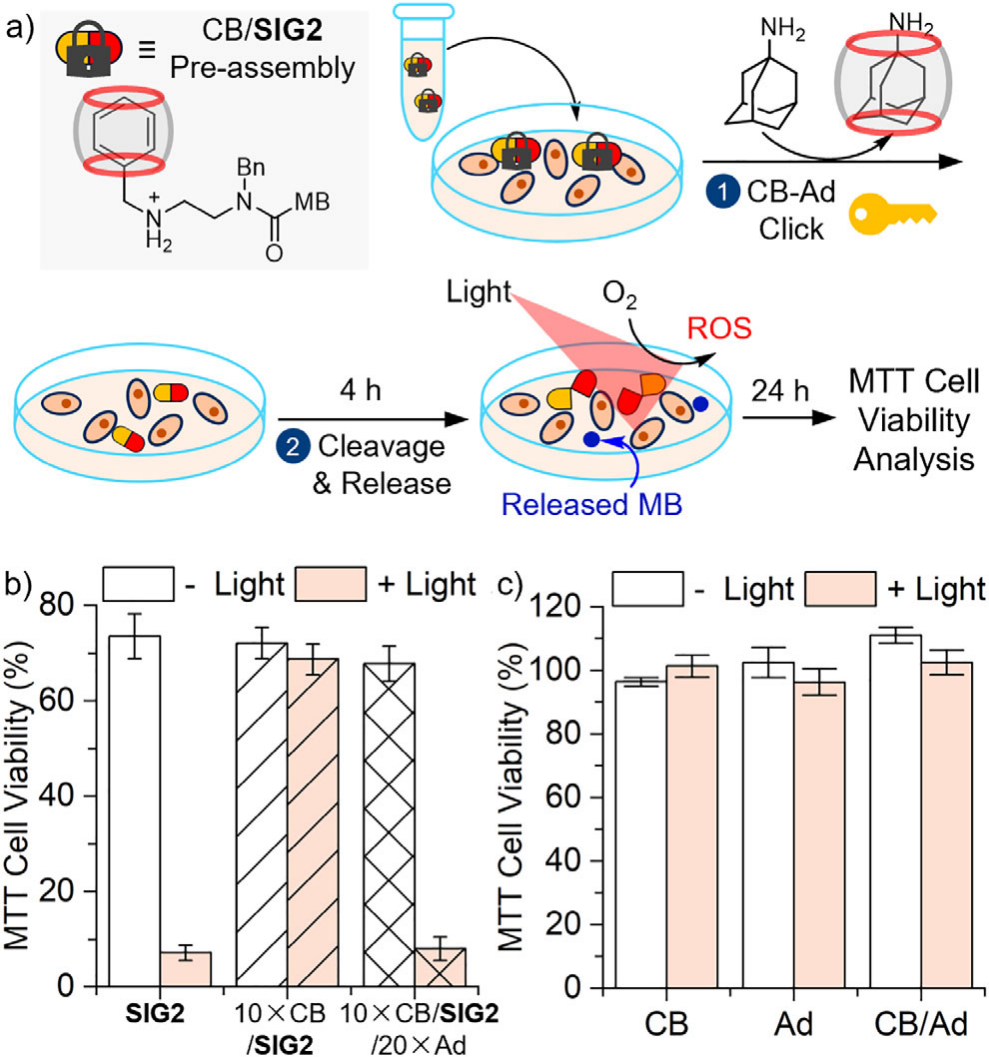
a) Schematic of the cellular experiments demonstrating supramolecular regulation of PDT using the CB‐Ad click‐to‐release platform. b) MTT assays show photoinduced cytotoxicity under various treatments. [**SIG2**]_0_ = 16 µM. c) Controls of CB (160 µM), Ad (320 µM), and CB/Ad (160 µM/320 µM) complex show minimal interference. Error bars: standard deviation from three repeats. 10 min irradiation at 13.2 mW/cm^2^.

This study presents a general strategy for controlling bond cleavage and cargo release through noncovalent click chemistry, as exemplified by the regulation of self‐immolative cleavage using the CB‐Ad click pair. Pre‐assembled CB‐SIG complexes are rationally designed to inhibit premature release, while the addition of a high‐affinity competing guest initiates CB‐Ad binding, which functions as a noncovalent click reaction to displace the SIG and trigger its preprogrammed cleavage for cargo release. As a proof‐of‐concept, we demonstrate the potential of this noncovalent click‐to‐release strategy for the controlled release of a photosensitizer, effectively regulating photodynamic cell killing in vitro. Despite its 10^5^–10^6^ M^−1^ affinity stabilizing most SIG molecules in the host‐guest complex, a key limitation of the current system is that residual unbound SIG still causes premature release before the competing guest is added, particularly at 37 °C. Future work will minimize this premature release by incorporating higher‐affinity binding motifs into the SIG platform. By extending the concept of noncovalent click chemistry to click‐to‐release systems, our platform broadens the structural and functional scope of bioorthogonal cleavage strategies with promising implications for material science and biomedical applications.

## Supporting Information

The Supporting Information is available free of charge online. Experimental details, supporting figures, synthetic procedures, ITC, UV–vis, fluorescence, and NMR spectra.

## Author Contributions

Xuancheng Fu led the study and contributed to the manuscript writing. Bowen Xu, Michelle Wu, Luke G. Westbrook contributed to the experimental work. Suman Maity contributed to the computational work. James H. Henderson and Yaoying Wu shared research resources. Katie A. Edwards contributed to the ITC studies. Atanu Acharya led the computational work. Xiaoran Hu conceived and oversaw the project, secured funding and resources, and contributed to the manuscript writing.

## Conflict of Interests

The authors declare no conflict of interest.

## Supporting information



Supporting Information

## Data Availability

The data that support the findings of this study are available in the Supporting Information of this article.
