# Supplementary material for: A Noncovalent Click‐to‐Release Strategy to Control Bond Cleavage and Prodrug Activation
Source: Angew Chem Int Ed Engl. 2026 Jan 18;65(9):e15594. doi: 10.1002/anie.202515594 (PMC12930019; doi:10.1002/anie.202515594)

Supporting Information

A Noncovalent Click-to-Release Strategy to Control Bond Cleavage and Prodrug Activation

Xuancheng Fu,^[a][b]^ Bowen Xu,^[a][b]^ Suman Maity,^[a][b]^ Michelle Wu,^[a][b]^ Luke G.Westbrook,^[a][b]^ James H. Henderson,^[b][c]^ Yaoying Wu,^[b][c][d]^ Katie A. Edwards,^[e]^ Atanu Acharya,* ^[a][b]^ Xiaoran Hu* ^[a][b]^

[a] Department of Chemistry, Syracuse University, Syracuse, New York, 13244, United States

[b] BioInspired Institute, Syracuse University, Syracuse, New York, 13244, United States

[c] Department of Biomedical & Chemical Engineering, Syracuse University, Syracuse, New York, 13244, United States

[d] Department of Microbiology and Immunology, State University of New York, Upstate Medical University, Syracuse, New York 13210, United States

[e] Department of Pharmaceutical Sciences, School of Pharmacy and Pharmaceutical Sciences, Binghamton University, Binghamton, New York, 13902, United States

* Overall correspondence should be addressed to Dr. Hu (xhu156@syr.edu); correspondence regarding computational results should be addressed to Dr. Acharya (achary01@syr.edu).

Contents

[1. General Considerations 2](#_Toc216947082)

[2. Supporting Figures 4](#_Toc216947083)

[3. Binding Studies 11](#_Toc216947084)

[4. Equilibrium Calculations 16](#_Toc216947085)

[5. Characterizations of SIG2 17](#_Toc216947086)

[6. PDT Experiments 20](#_Toc216947087)

[7. Synthetic Details 22](#_Toc216947088)

[8. NMR Spectrum 35](#_Toc216947089)

# 1. General Considerations

All reactions were conducted under standard air-free conditions under an atmosphere of nitrogen gas with magnetic stirring unless otherwise mentioned. All reactants and solvents were purchased from commercial suppliers and used without further purification unless otherwise noted. Flash chromatography was performed on a Biotage Isolera System with Yamazen Corp. universal silica gel columns (Pore Size 60 angstroms, Particle Size 40-63 microns).

NMR spectra were acquired on a Bruker Avance III HD 400 and 800 MHz spectrometer. ^1^H NMR spectra are reported relative to residual protonated solvent (7.26 ppm for chloroform, 4.79 ppm for water, 3.31 ppm for methanol, 2.05 ppm for acetone). ^13^C NMR spectra are reported relative to residual protonated solvent (77.16 ppm for chloroform, 49.00 ppm for methanol, 29.84 and 206.26 for acetone). Multiplicity abbreviations are as follows: s = singlet, d = doublet, t = triplet, q = quartet, dd = doublet of doublets, ABq = AB quartet, m = multiplet, br = broad.

Mass spectra were acquired on a DART-SVP (Direct Analysis in Real Time) ion source (IonSense, Saugus, MA) coupled to an Exactive Orbitrap mass spectrometer (Thermo Scientific, Bremen, Germany) at the Cornell Chemistry Mass Spectrometry Facility.

All solution optical spectra were acquired of samples in quartz cuvettes. Electronic absorbance spectra were acquired with an Evolution 201 UV-visible spectrophotometer in double-beam mode using a solvent-containing cuvette for background subtraction spectra of solution samples. Fluorescence spectra were measured with an Agilent Cary Eclipse G9800A Fluorescence Spectrophotometer.

Fluorescence images were acquired using a Zeiss LSM 980 Airyscan 2 confocal super-resolution microscope (Carl Zeiss, Germany) equipped with a GaAsP detector array. Excitation wavelengths were set to 405 nm for Hoechst 33342, 488 nm for **SIG1_NBD_**, and 633 nm for LysoTracker. Images were collected using a Plan-Apochromat 20×/0.8 NA objective lens. To ensure quantitative comparability, laser power, detector gain, and acquisition parameters were maintained constant across all samples.

**Light Irradiation Setup**: Light irradiation was performed using a 3000 K LED lamp equipped with a 560 nm long-pass optical filter. The LED source was fixed on a tripod stand and positioned vertically above the samples. For cuvette and cellular experiments, quartz cuvettes and 96-well plates were placed directly below the vertically mounted light source under identical illumination conditions. The light intensity at the sample surface was measured using a digital photometer (Industrial Fiber Optics, #IF PM) and determined to be 2.2 mW cm^-2^ for the SOSG measurement setup, and 13.2 mW cm^-2^ for in vitro PDT setup.

**Isothermal Titration Calorimetry** (ITC) measurements were conducted using a Nano ITC Calorimeter (TA Instruments, New Castle, DE) at 25 °C. For titrations in water, CB solutions (1.0 mM) were titrated into 50 µM guest solutions (cell volume = 170 µL) in 42 injections of 1.2 µL each, with a 200 s interval between injections. For titrations in 1× MES buffer (pH 6.5), 0.4 mM CB was titrated into 50 µM **SIG1_OMe_** under otherwise identical conditions, using 25 injections of 2 µL each. The raw thermograms were baseline-corrected, and heats of dilution were subtracted based on the final saturated injections. The corrected data were analyzed using NanoAnalyze software (v 4.0.2, TA Instruments) employing a one-set-of-sites binding model.

For a representative experiment conducted in 1× MES (2-(N-morpholino)ethanesulfonic acid) (pH 6.5) buffer solutions, CB, SIG, and adamantane were dissolved into DI water in the order of the compounds as listed by name (e.g., the sequence of addition for the CB/SIG/Ad group is CB🡪SIG🡪Ad). Into this mixture solution was added 20 v/v % of pH 6.5 5×MES buffer, resulting in a final concentration of 1×MES buffer. Fluorescence-based kinetics measurements were then carried out.

Deuterated phosphate buffer (home-made, pH 6.5 in D_2_O) was used in NMR-monitored self-immolation experiments. For a representative experiment, a DMSO-d_6_ solution of SIG, a D_2_O solution of CB, and a D_2_O solution of Adamantane were sequentially added into D_2_O in the order of the compounds as listed by name (e.g., the sequence of addition for the CB/SIG/Ad group is CB🡪SIG🡪Ad). Into this mixture solution was added pH 6.5 deuterated phosphate buffer, resulting in a final buffer concentration of 100 mM. NMR kinetics measurements were then carried out.

# 2. Supporting Figures


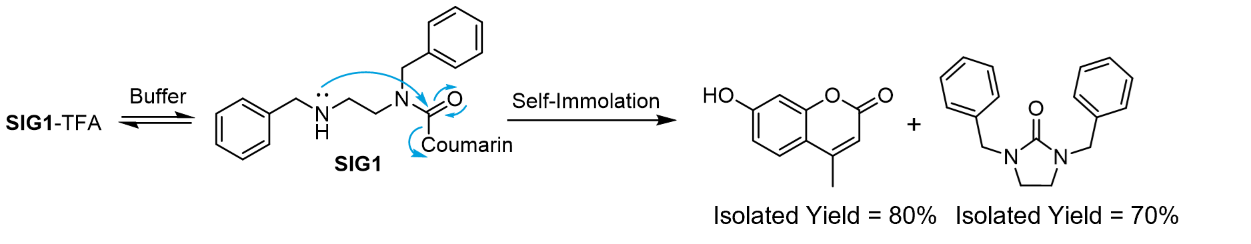


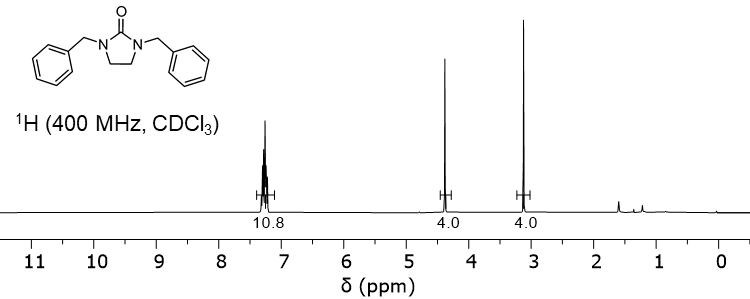


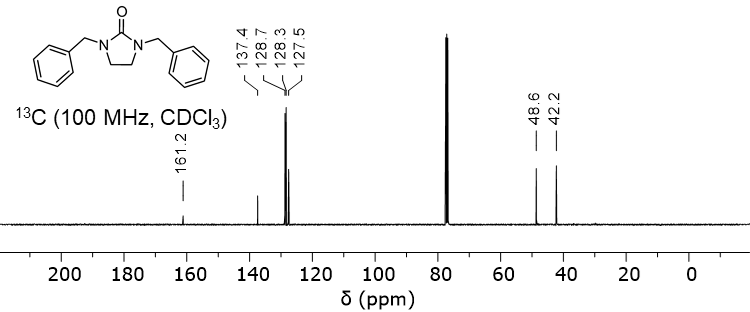


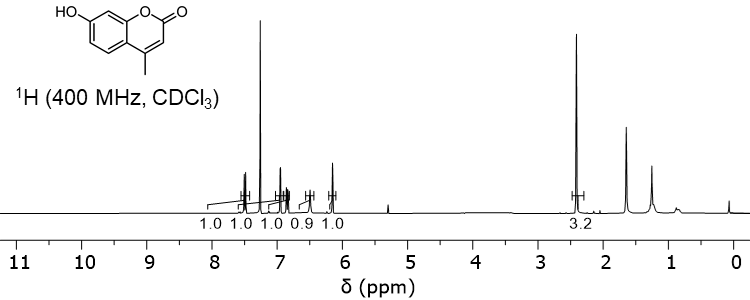


Figure S1. The self-immolation products of **SIG1** were isolated and confirmed by NMR, validating that the SIG scaffold exhibits the expected self-immolative reactivity. Experimental conditions: a solution of **SIG1**-TFA in 2.23 mL DMSO (24.8 mg, 44.5 µmol) was added to 17.84 mL of DI water, followed by the addition of 2.23 mL of 1 M pH 6.5 phosphate buffer. The mixture was incubated overnight at room temperature, then extracted with DCM (50 mL) three times. The combined organic phases were washed with brine (50 mL), dried over Na_2_SO_4_, filtered, and concentrated under reduced pressure. The crude product was separated by column chromatography (hexanes/ethyl acetate) to yield the cyclization product (9.5 mg, 80%) and 4-hydroxy-7-methylcoumarin (5.5 mg, 70%). Additionally, the identity of the cyclization product was further supported by HRMS (DART/Orbitrap, m/z): calcd for [C17H19N2O1]^+^ (M+H)^+^, 267.1482; found, 267.1489.


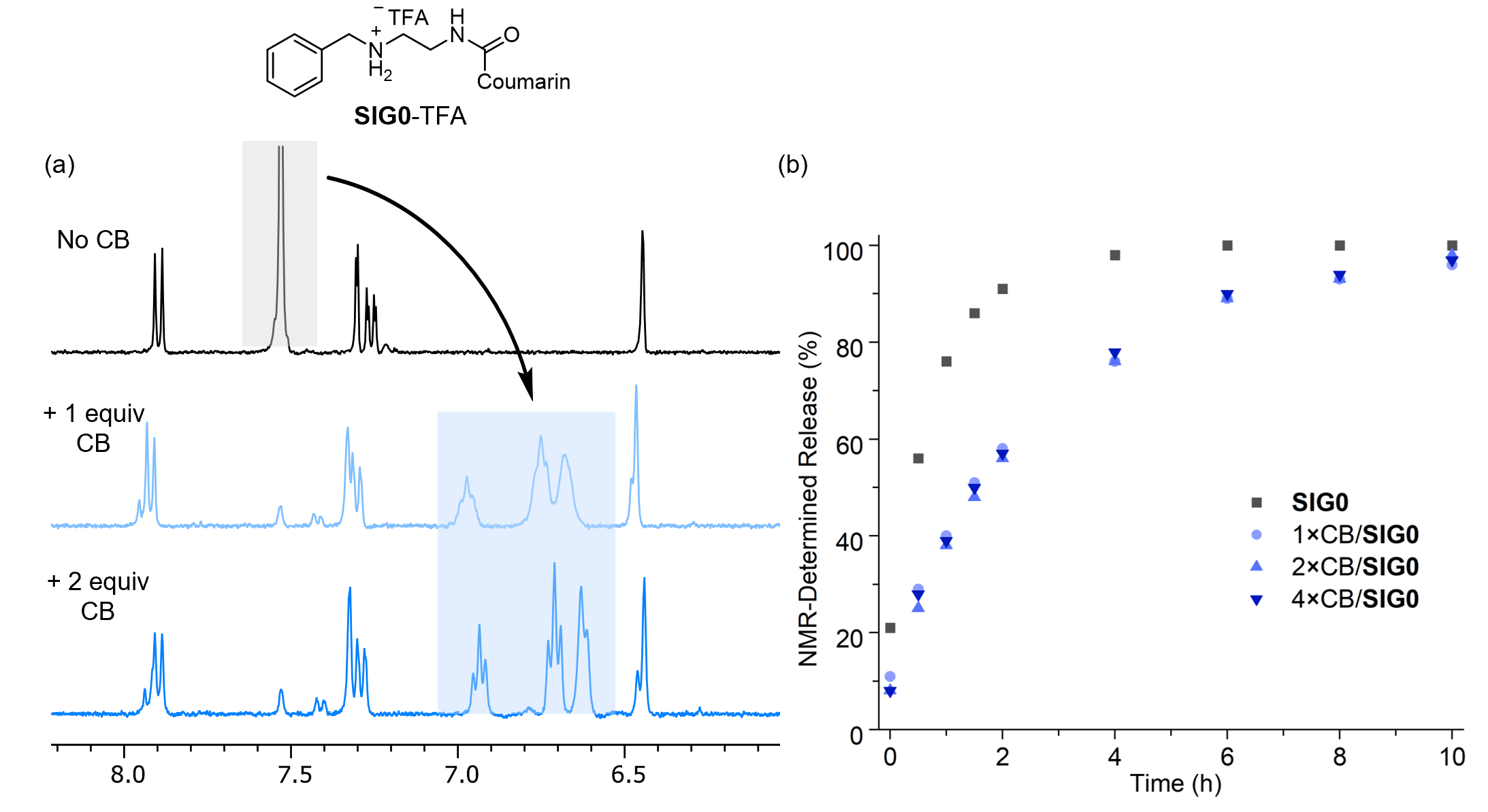


Figure S2. Effect of host–guest interactions on the cyclization–elimination reaction using a preliminary model compound **SIG0**. (a) The structure and ^1^H NMR spectra of **SIG0**-TFA in D_2_O before and after adding CB, confirming host-guest binding interactions ([**SIG0**-TFA]_0_ = 2 mM, D_2_O with 10% DMSO-d_6_). (b) NMR-monitored coumarin release from 2 mM solutions of **SIG0** as a function of the CB stoichiometry. **SIG0**-TFA is stable in pure water before adding the buffer. At time zero, PBS buffering salts were added (see Supporting Information Section 1) to raise the pH to 6.5. **SIG0** underwent rapid self-immolation, releasing over 90% of payloads within 2 h. The introduction of CB slightly yet noticeably slowed the reaction rates. All experiments were conducted at room temperature.


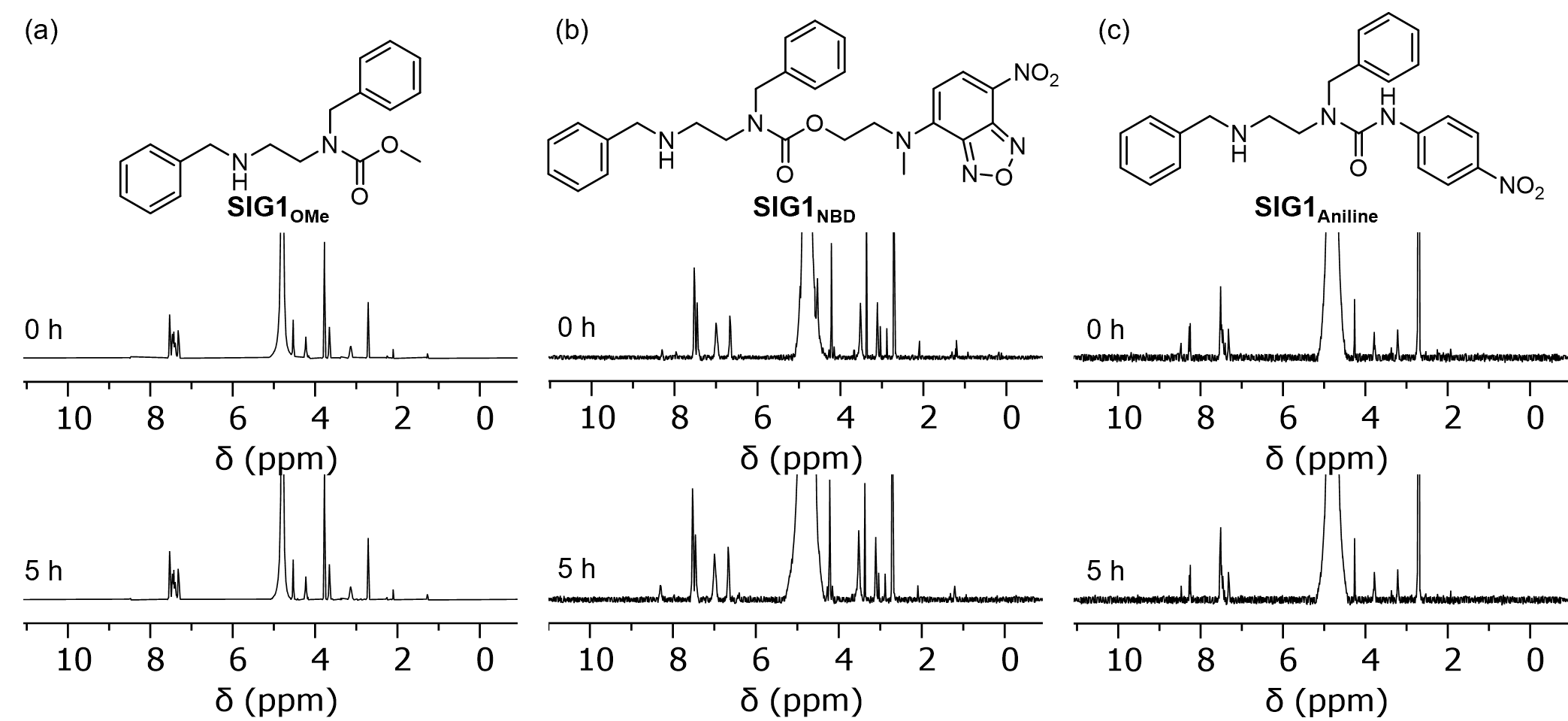


Figure S3. ^1^H NMR spectra of 2 mM solution of **SIG1_OMe_** (a), **SIG1_NBD_** (b) and **SIG1_Aniline_** (c) in 90% pH 6.5 phosphate buffer in D_2_O with 10% DMSO-d_6_ recorded at 0 h and 5 h post incubation at room temperature. All compounds remained stable under these conditions, indicating that the **SIG1** platform can not directly release alcohol or amine payloads.


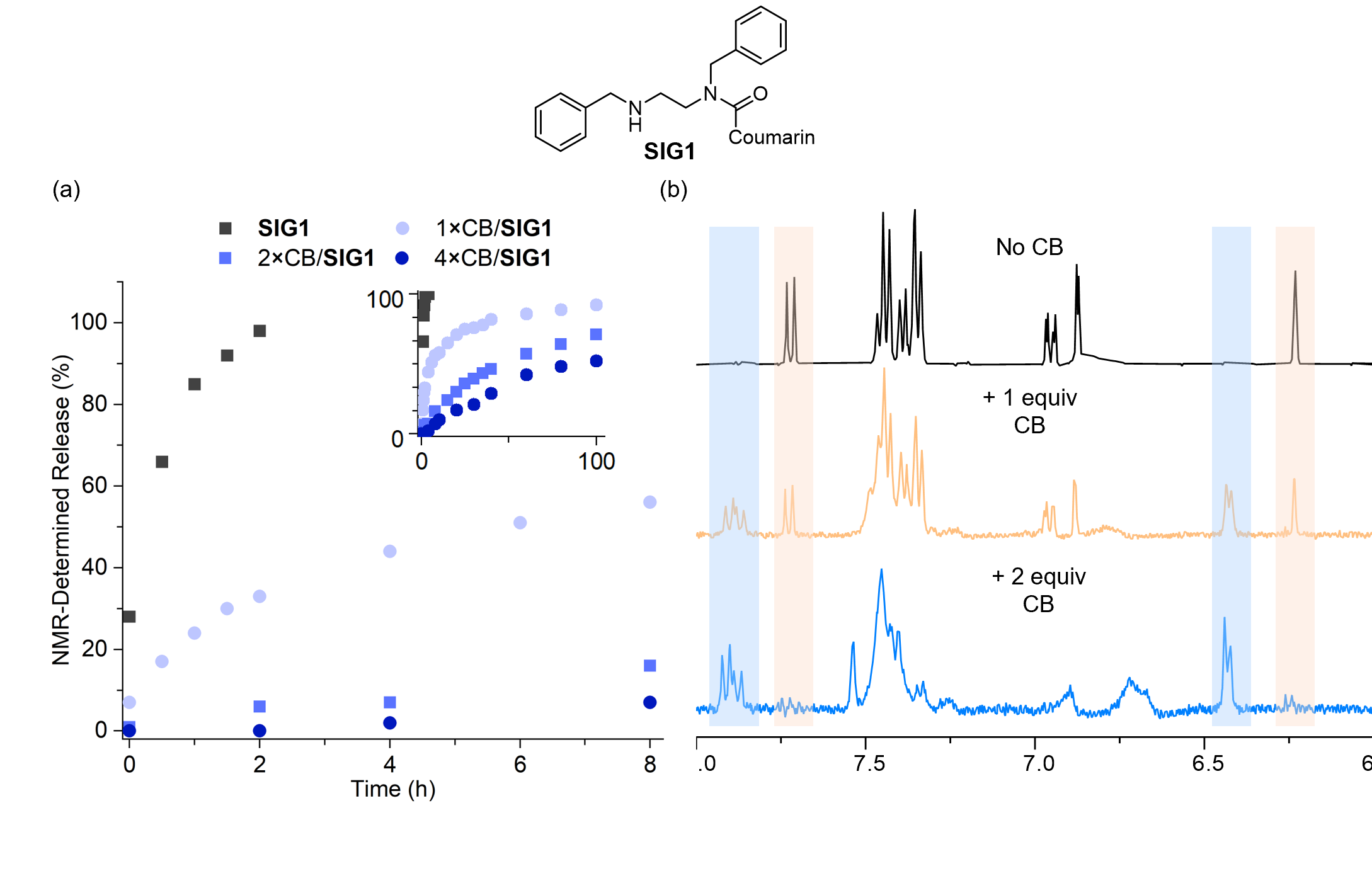


Figure S4. (a) NMR-measured percentage of coumarin released from **SIG1** as a function of the CB stoichiometry (2 mM **SIG1** in 90% pH 6.5 phosphate buffer in D_2_O and 10% DMSO-d_6_). The introduction of 1×, 2×, and 4× CB extended the pseudo-first-order reaction half-life (t_1/2_) of coumarin release from 0.3 to 4.8, 44.5, and 88.4 hours (room temperature). (b) Representative ¹H NMR spectra of 2 mM **SIG1** after 2 h incubation in the presence of 0×/1×/2× CB. The light-orange regions highlight proton signals from the released coumarin, while the light-blue regions correspond to coumarin still conjugated within **SIG1**.


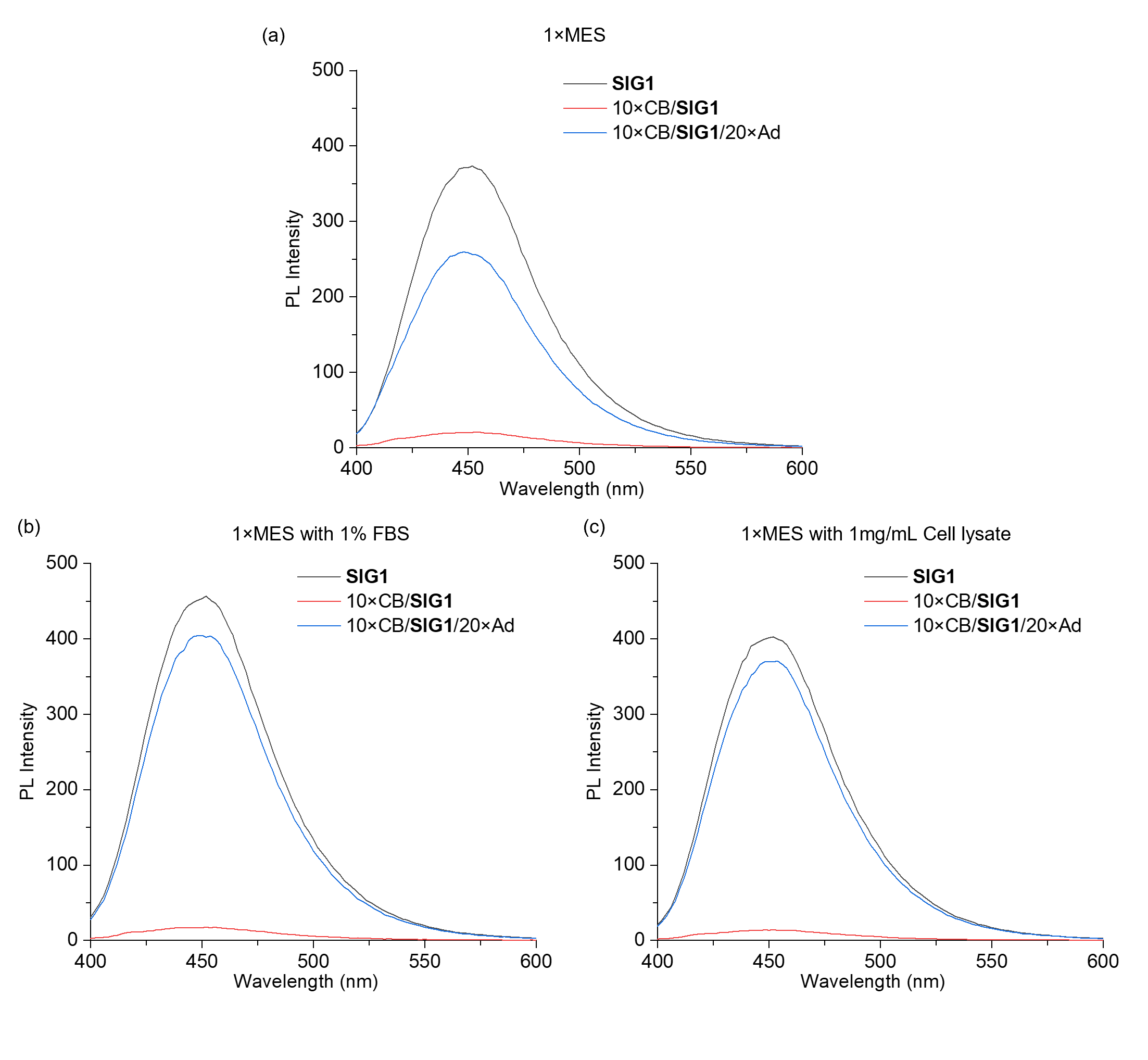


Figure S5. Fluorescence spectra of **SIG1**, 10×CB/**SIG1**, and 10×CB/**SIG1**/20×Ad in (a) 1×MES buffer, (b) 1×MES buffer containing 1% FBS, and (c) 1×MES buffer containing 1 mg/mL cell lysate after 4 hours of incubation. [**SIG1**]_0_ = 10 µM.


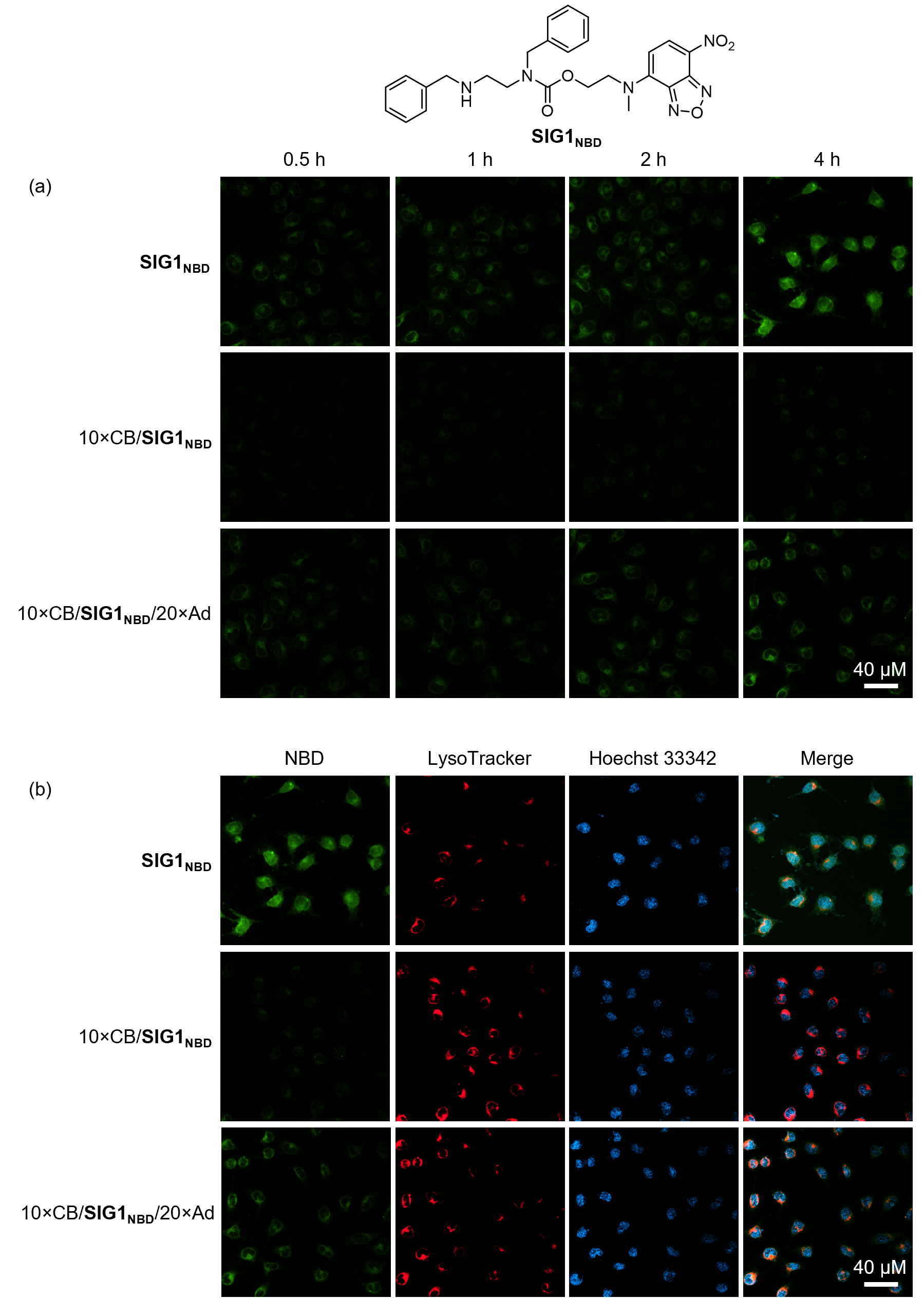


Figure S6. Cellular permeability and subcellular localization studies of **SIG1_NBD_.** (a) Confocal fluorescence images of *HeLa* cells after respective treatments: **SIG1_NBD_** exhibited good cell permeability, with strong intracellular fluorescence within 0.5 h. In contrast, the CB/**SIG1_NBD_** complex showed minor cellular uptake even after 4 h, likely due to CB masking the cationic sites of **SIG1_NBD_**. Addition of Ad partially restored cellular uptake. (b) Confocal fluorescence images elucidate the subcellular localization of *HeLa* cells incubated with **SIG1_NBD_**, CB/**SIG1_NBD_**, and CB/**SIG1_NBD_**/Ad for 4 hours. Green: NBD fluorescence; red: LysoTracker (lysosomes); blue: Hoechst 33342 (nuclei). **SIG1_NBD_** was not found to colocalize with lysosomes or nuclei. Experimental details: *HeLa* cells were incubated in DMEM media, with 10% fetal bovine serum (FBS) at 37 °C and 5% CO_2_. Approximately 5,000 cells suspended in 100 µL of medium were seeded into each well of a 96-well plate and allowed to adhere for 12 h prior to treatment. The culture medium was then replaced with 100 µL of 1×MES buffer containing the corresponding sample solutions— **SIG1_NBD_** (10 µM), CB/**SIG1_NBD_** (100 µM/10 µM), or CB/**SIG1_NBD_**/Ad (100 µM/10 µM/200 µM)—depending on the specific assay. After 0.5, 1, 2, and 4 h of co-incubation, the supernatant was removed, and the cells were stained with LysoTracker and Hoechst 33342 following the manufacturer’s instructions. Confocal laser scanning microscopy (CLSM) images were subsequently acquired under identical imaging settings.


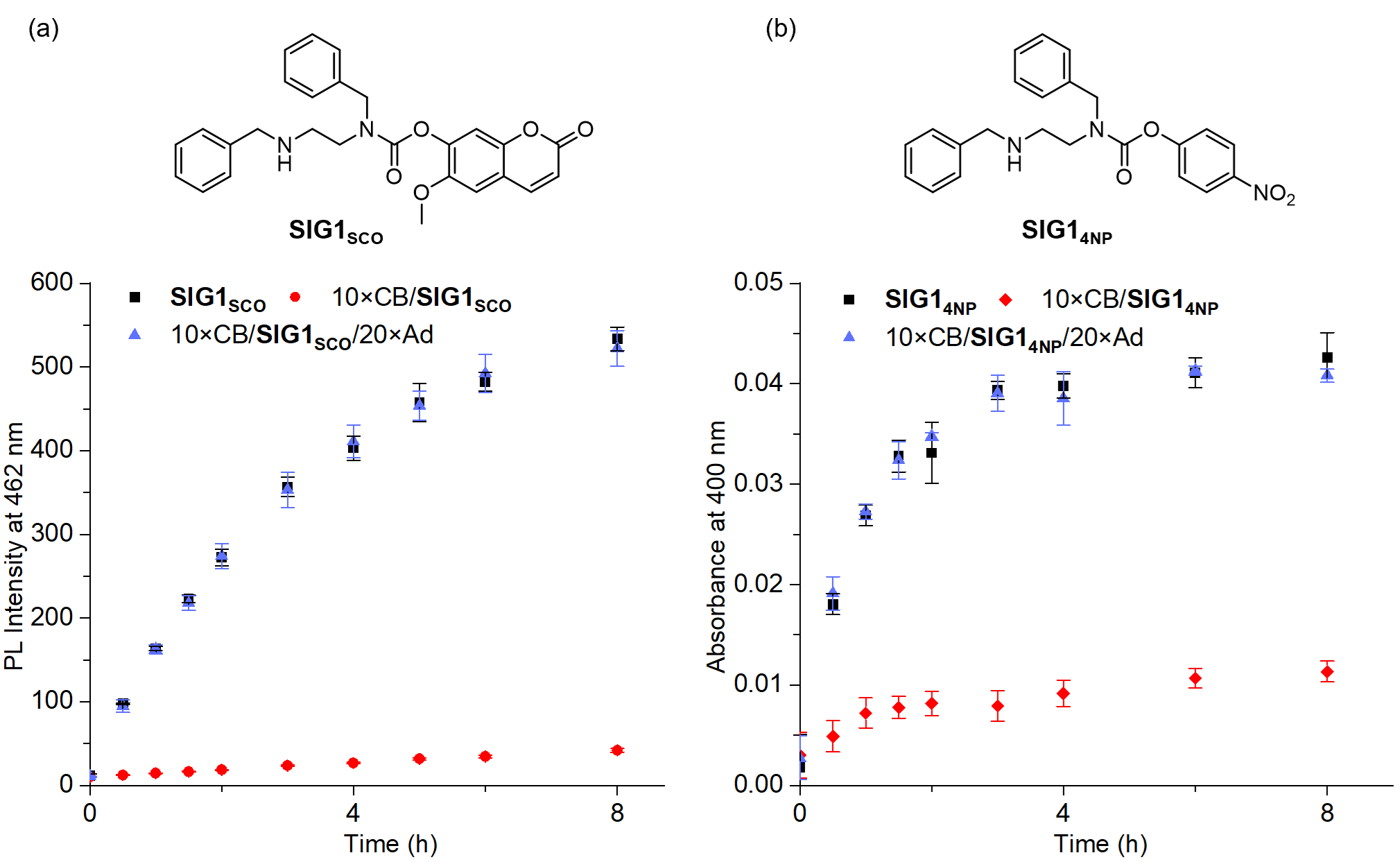


Figure S7. (a) Structure and time-dependent fluorescence changes (λ = 462 nm) of **SIG1_SCO_** (black), 10×CB/**SIG1_SCO_** (red), and 10×CB/**SIG1_SCO_**/20×Ad (blue) in 1×MES buffer. [**SIG1sco**]_0_ = 10 µM. The fluorescence intensity reflects the release of scopoletin. Complexation with CB effectively suppressed the self-immolation of **SIG1_SCO_**, while subsequent addition of Ad restored the release, confirming CB/Ad-regulated control of the phenolic cargo. (b) Structures and time-dependent absorbance changes (λ = 400 nm) of **SIG1_4NP_** (black), 10×CB/**SIG1_4NP_** (red), and 10×CB/**SIG1_4NP_** /20×Ad (blue) in 1×MES buffer. [**SIG1_4NP_**]_0_ = 10 µM. The absorbance at 400 nm reflects the release of nitrophenol. CB complexation efficiently inhibited the self-immolation of **SIG1_4NP_**, while addition of Ad partially restored the release, confirming that the CB/Ad regulatory mechanism is generalizable across different phenolic cargos. Error bars: standard deviation from three repeats.


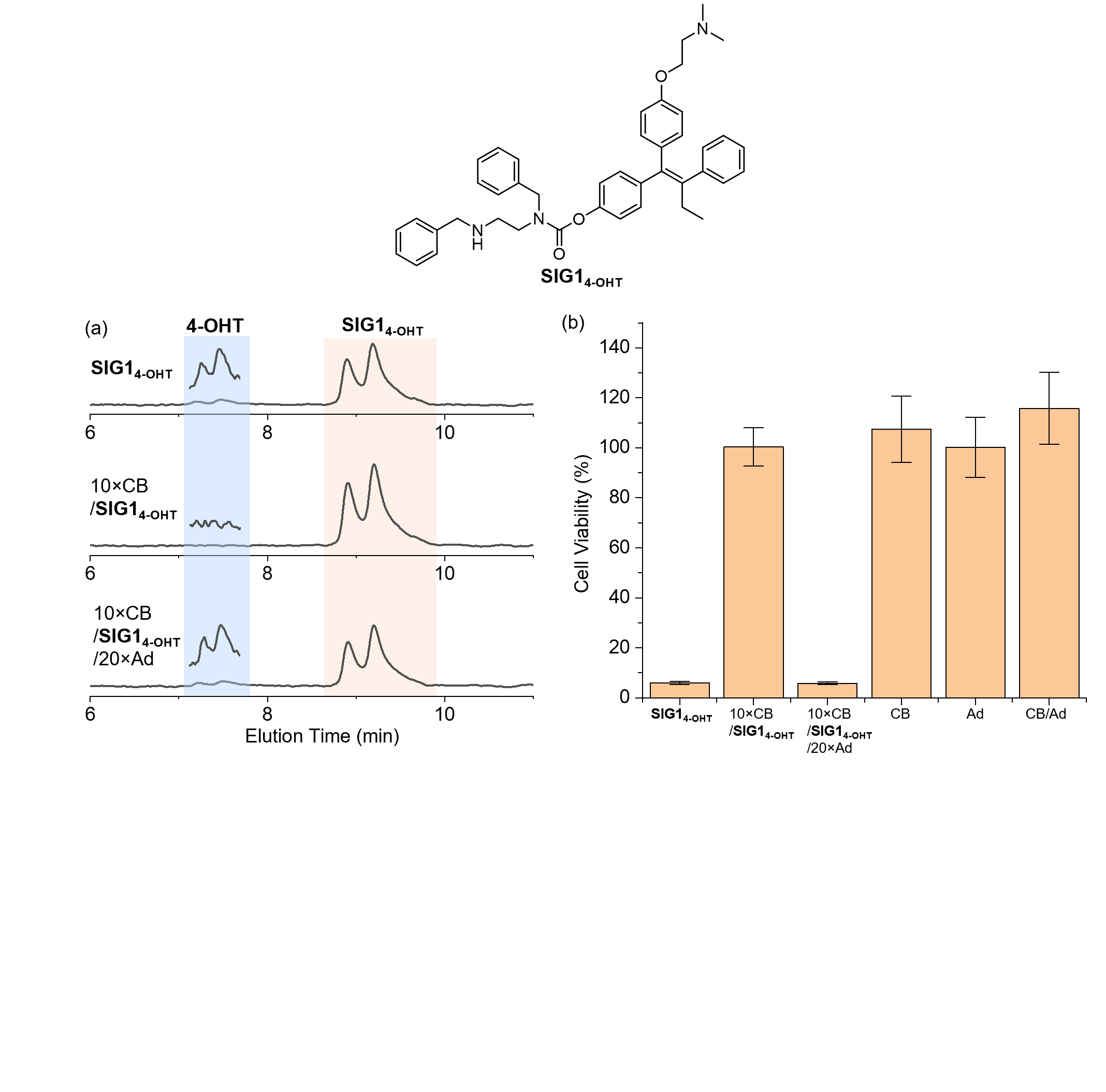


Figure S8. (a) HPLC analysis of **SIG1_4-OHT_**, 10×CB/**SIG1_4-OHT_**, and 10×CB/**SIG1_4-OHT_**/20×Ad after 4 h incubation in 1×MES buffer at room temperature. (E/Z)-4-hydroxy Tamoxifen (4-OHT) is a phenol-based anti-cancer drug that induces apoptosis in cancer cells (Ref: *Sci Rep* **2023**, *13*, 18549). Free 4-OHT drug released from **SIG1_4-OHT_** reached 2.08 μM after 4 h incubation. Upon addition of 10×CB, no detectable 4-OHT release was observed, indicating that CB complexation suppresses self-immolation. After activation with Ad, the 10×CB/**SIG1_4-OHT_**/20×Ad complex released 2.11 μM 4-OHT, a level identical to the **SIG1_4-OHT_** group, confirming that Ad addition fully restores release. [**SIG1_4-OHT_**]_0_ = 16 μM. (b) Cell viability of *HeLa* cells after treatment with the indicated samples. All solutions in 1×MES buffer were incubated with *HeLa* cells for 4 h at room temperature, followed by replacement with fresh medium and an additional 20 h incubation, after which viability was assessed by the MTT assay. The results show that only the **SIG1_4-OHT_** and 10×CB/**SIG1_4-OHT_**/20×Ad groups exhibit pronounced cytotoxicity, consistent with their detectable 4-OHT release in the HPLC analysis. In contrast, 10×CB/**SIG1_4-OHT_** remains non-toxic due to suppressed release. These findings demonstrate that CB complexation inhibits the therapeutic effect of the phenol-type prodrug, while subsequent addition of the “noncovalent click trigger” Ad reactivates the therapeutic effect by restoring its release. Both CB (160 μM), Ad (320 μM), and CB/Ad (160 μM/320 μM) exhibit negligible cytotoxicity. [**SIG1_4-OHT_**]_0_ = 16 μM. Error bars: standard deviation from three repeats.

# 3. Binding Studies

**Overview and Discussion of Supporting Information Section 3**: Although the solution of **SIG1**-TFA salt in pure water is stable, it undergoes spontaneous self-immolation and cargo release once buffer is added. Therefore, we developed **SIG1_OMe_** as an inactive model compound (Figure S3a) to elucidate the binding properties of the **SIG1** platform under buffered solution (Figure 2c and **Supporting Information 3.1**), where **SIG1** would otherwise undergo rapid decomposition.

Additionally, we compared the binding properties of **SIG1** in pure water to those of **SIG1_OMe_** in pure water using NMR and ITC (**Supporting Information 3.2**) methods. The similar behaviors observed for **SIG1** and **SIG1_OMe_** in pure water indicate that **SIG1_OMe_** serves as a reliable structural analogue representing the overall SIG scaffold.

**3.1. NMR Confirmation of CB Binding to the Benzylamine Motif in SIG1_OMe_ in pH 6.5 Buffer Solutions**


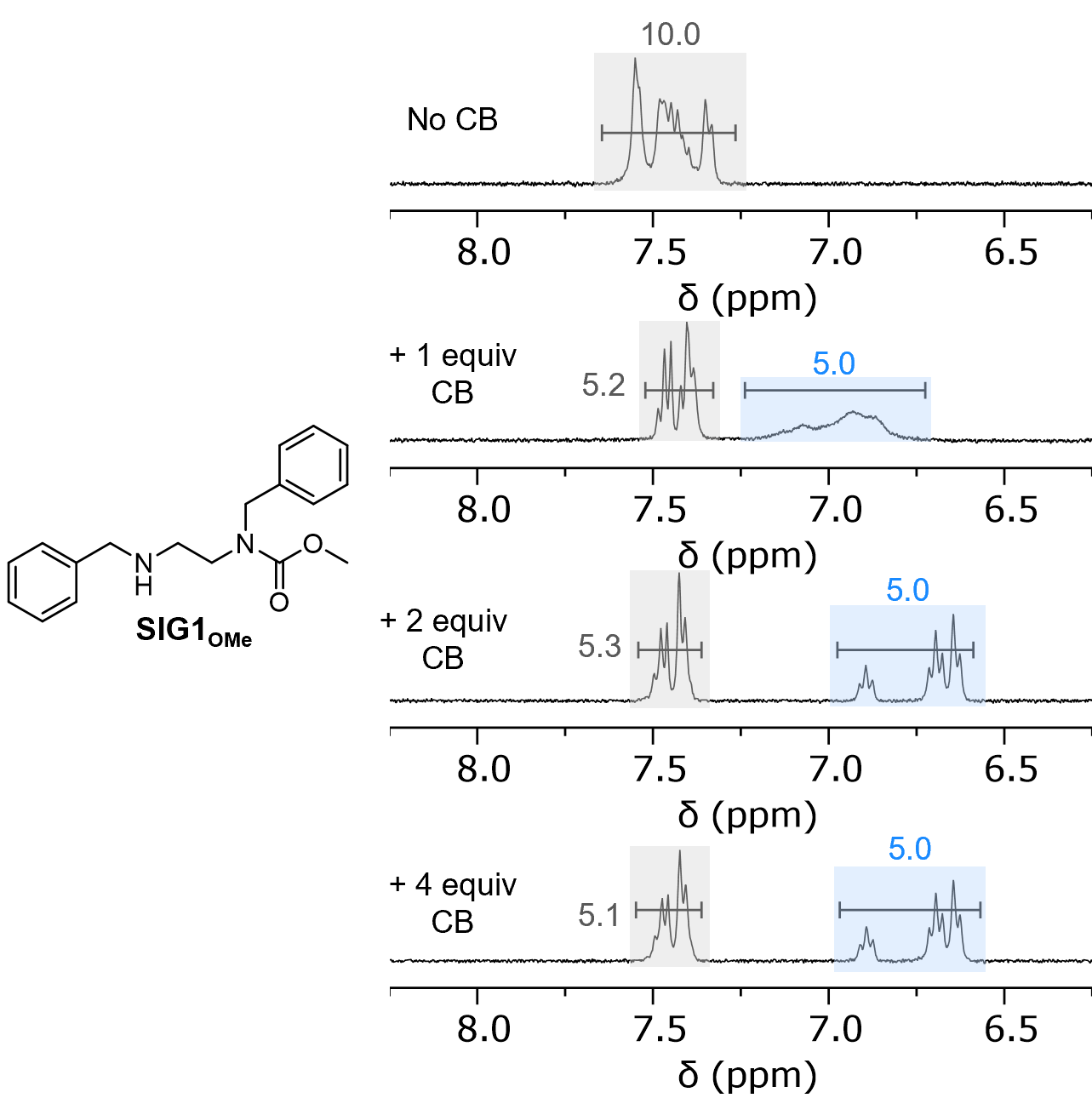


Figure S9. ^1^H NMR spectra of 1 mM **SIG1_OMe_** in 1× deuterated MES buffer as a function of CB equivalents. This result, along with 2-D NMR results in Figure S10 and Figure S11, confirms a single binding event at the benzylamine motif, corroborating the 1:1 binding stoichiometry observed in buffer solution from ITC measurements (Figure 2c).


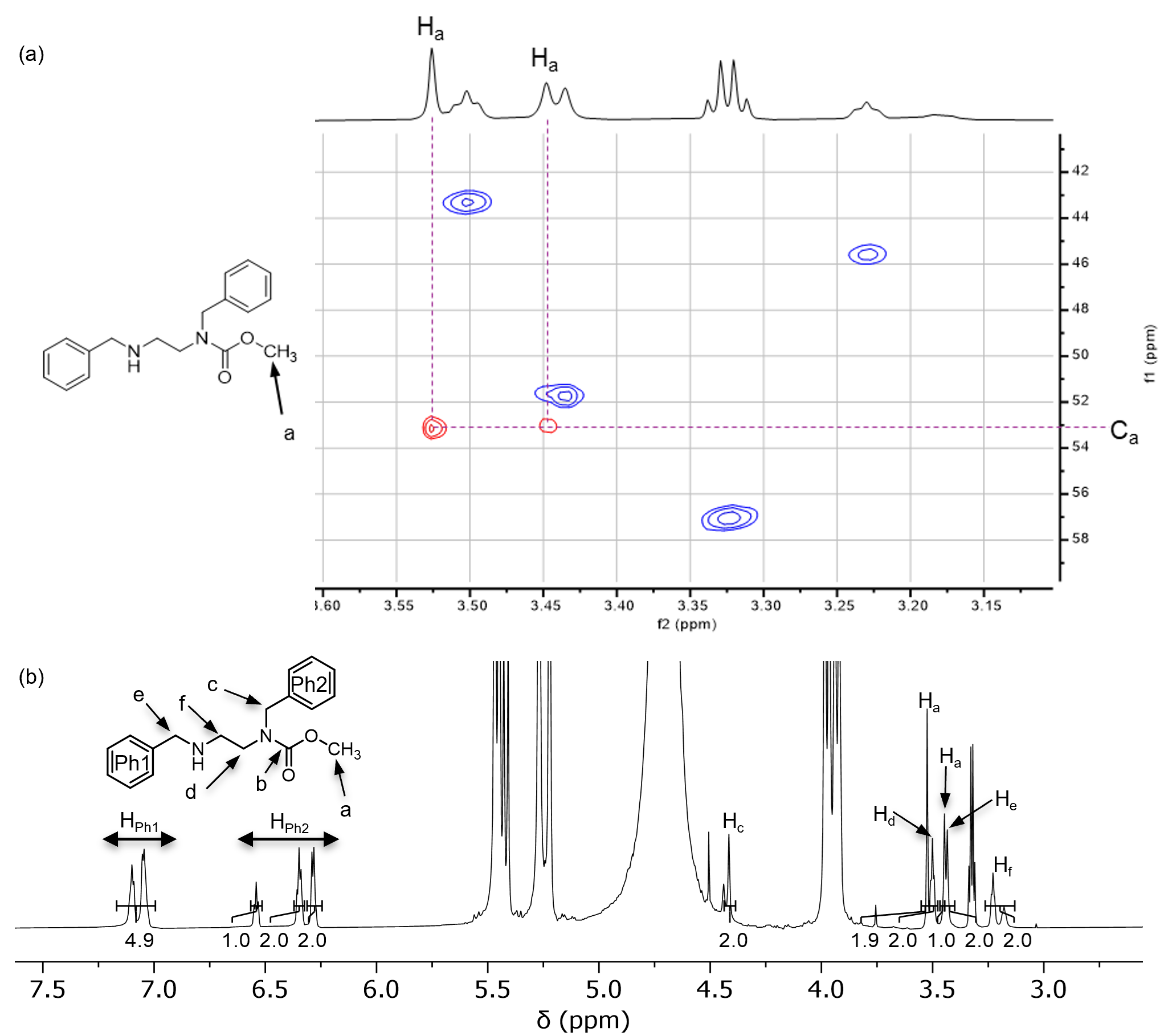


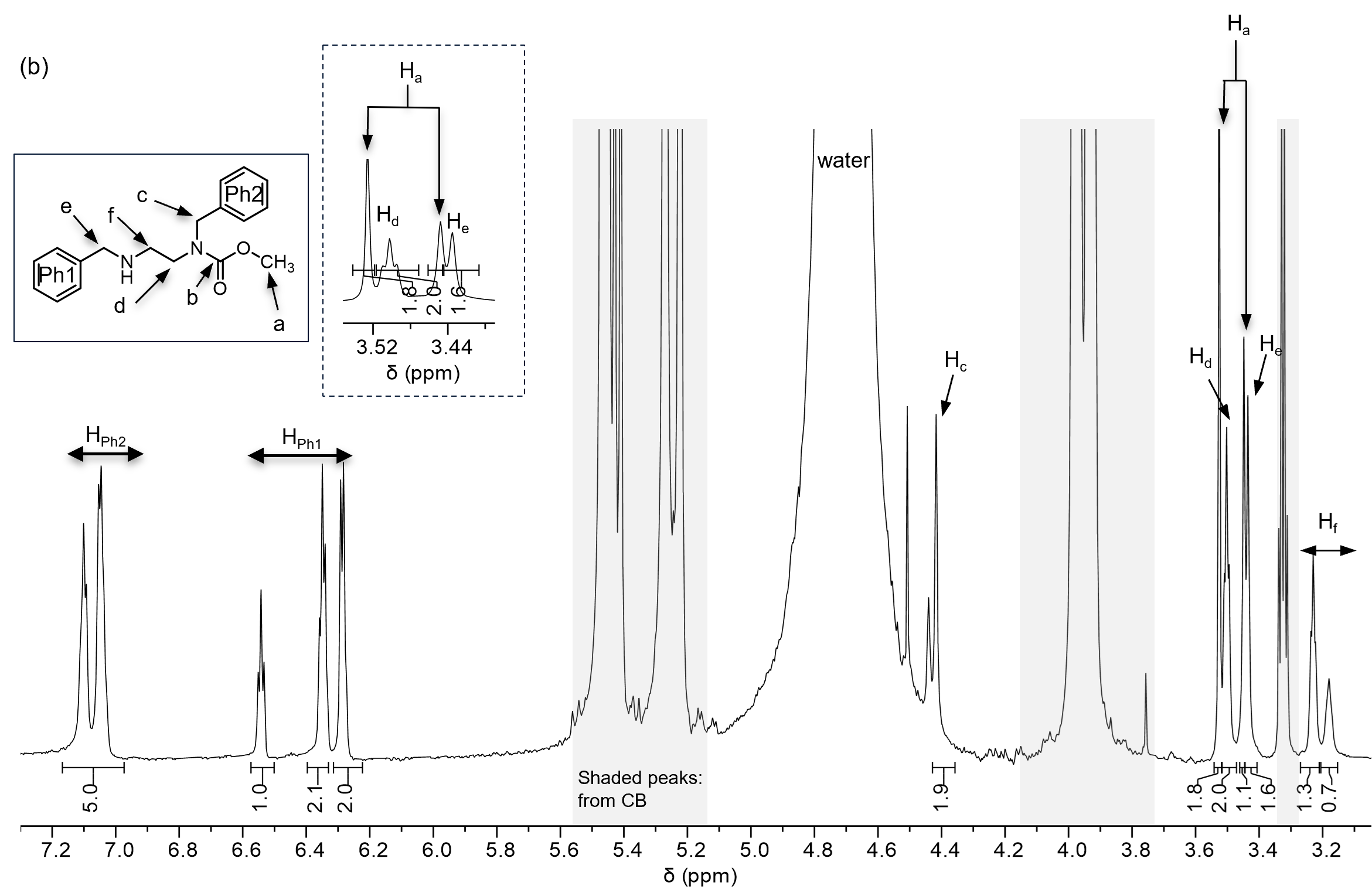


Figure S10. (a) ^1^H–^13^C HSQC spectrum of 1 mM 4×CB/**SIG1_OMe_** in 100 mM phosphate buffer (pH 6.5) at 5 °C (all 2-D NMR experiments were conducted at 5 °C for improved NMR signal quality). The red signal (positive phase) is attributed to the methyl correlations (see arrow). **SIG1_OMe_** shows two sets of rotamer signals (3.53 and 3.45 ppm) due to restricted rotation at the carbamate group. (b) Corresponding integrated ^1^H NMR spectrum of the same solution.


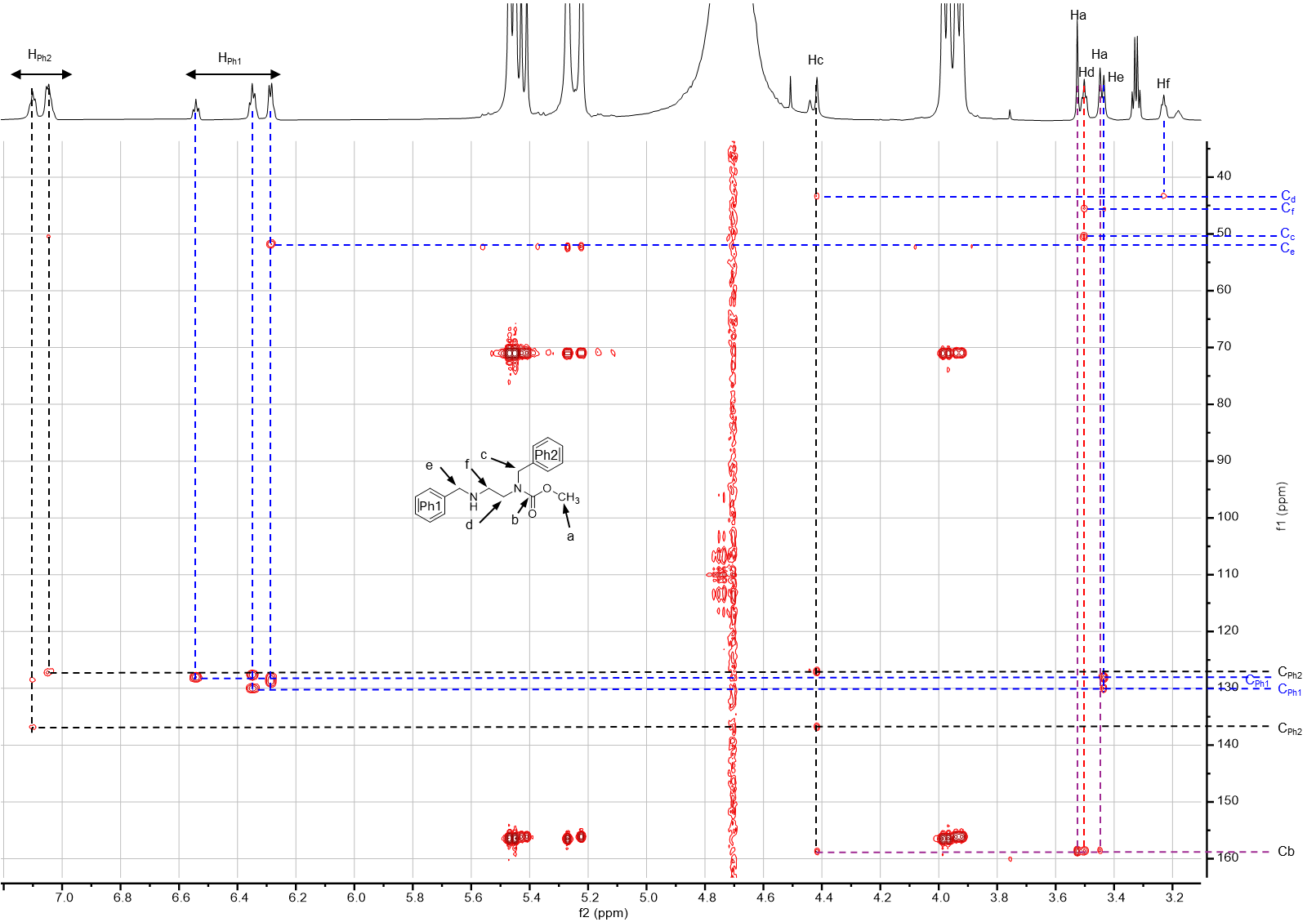
Figure S11. HMBC NMR analysis enabled the assignment of corresponding NMR resonances. The HMBC results show that proton signals observed in the 7.0-7.15 ppm range and the 6.20 ppm-6.6 ppm range correspond to the aromatic protons of the benzylcarbamate (H_ph2_) and benzylamine (H_ph1_) groups, respectively. The 6.25-6.6 ppm peaks (H_ph1_) exhibit changes in chemical shift induced by host-guest interactions, whereas 7.0-7.15 ppm peaks show only minor shifts. Thus, CB-**SIG1_OMe_** host-guest occurs selectively at the H_ph1_ benzylamine motif.

**3.2 Investigation of CB-SIG1 Binding in Pure Water**


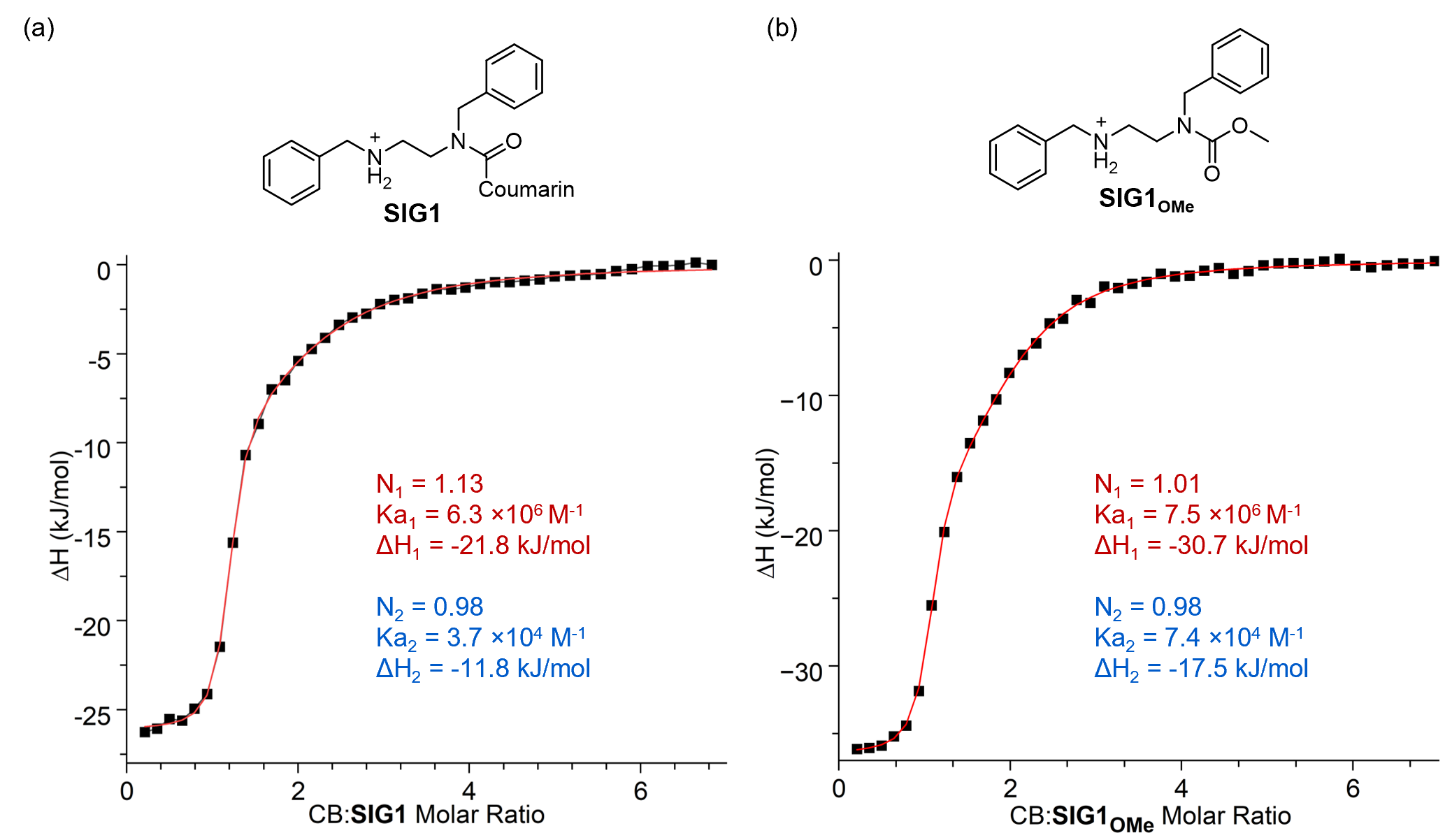


Figure S12. ITC titration of **SIG1** and **SIG1_OMe_** with CB in pure water. Both SIG compounds exhibit two binding events: a strong primary interaction at the benzylamine group (as assigned from 2D NMR experiments in Section 3.1) and a weaker secondary interaction attributed to the benzyl group adjacent to the carbamate group. Compared to the ITC results in pure water described in this figure, the buffered solutions (Figure 2c) show only a single binding event with a Ka value of 1.1 ×10^6^ M^-1^, as the higher ionic strength weakens both interactions and renders the secondary binding too weak to observe.


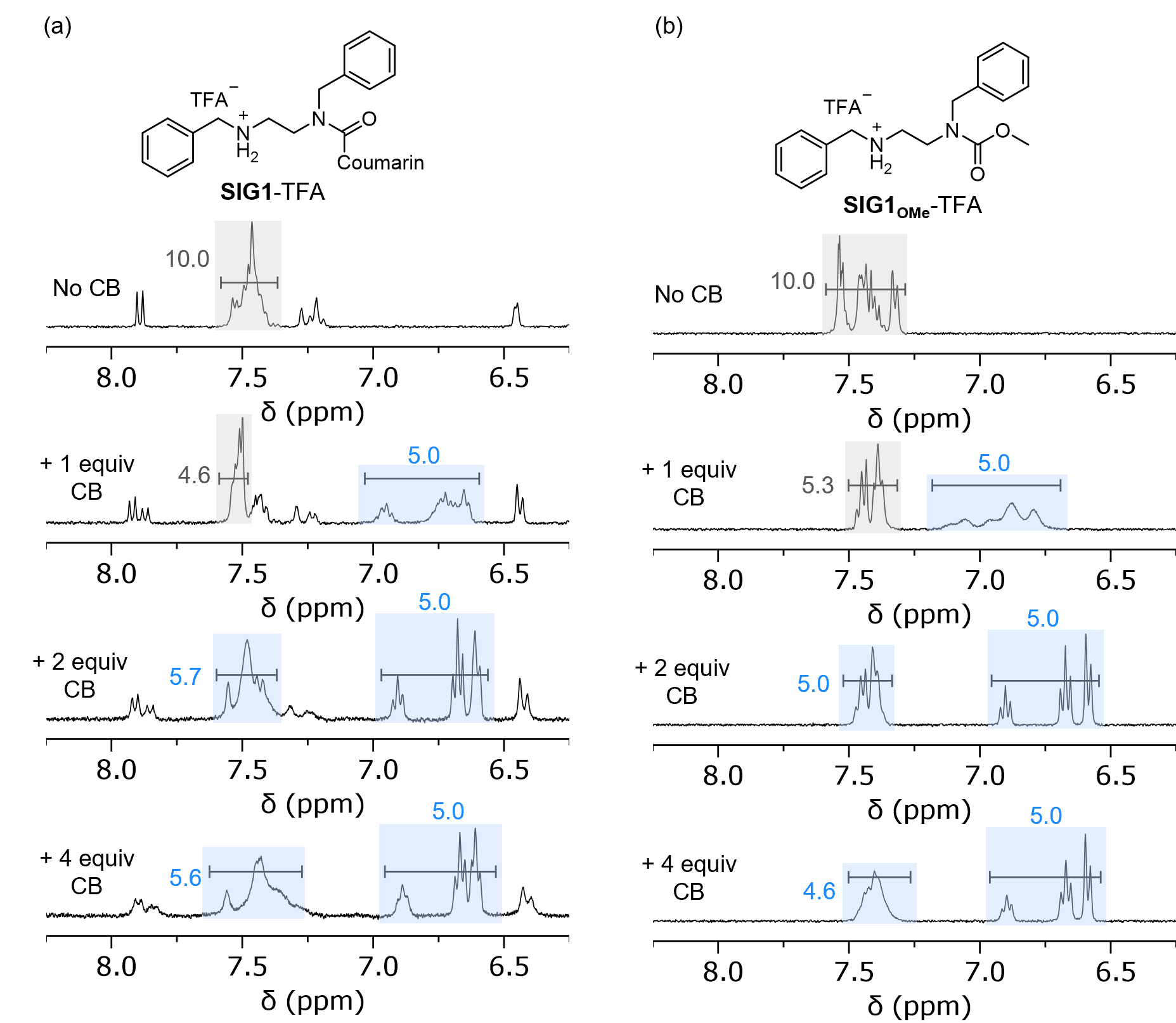


Figure S13. ^1^H NMR spectra of 1 mM **SIG1**-TFA (a) and **SIG1_OMe_**-TFA (b) in D_2_O as a function of CB equivalents. In both systems, (1) the addition of 1×CB led to an upfield shift of approximately five aromatic protons from 7.5 ppm to 6.5–7.0 ppm, suggesting host-guest interactions at one of the two benzyl groups. (2) The remaining five aromatic proton signals retained sharp line shape, indicating minimal binding interactions. (3) Upon further addition of CB, the unshifted peaks gradually broadened, implying weak secondary interactions at this site that now exist in dynamic equilibrium between free and weakly bound states. (4) In contrast, the peaks that initially shifted to 6.5–7.0 ppm became sharper at higher CB concentrations, consistent with near-complete caging of the high-affinity motif, leading to reduced exchange broadening. This progression suggests both SIG molecules possess a strongly binding motif and a weakly binding motif in pure water, corroborating ITC results discussed in Figure S12.

# 4. Equilibrium Calculations

We studied the effect of CB-binding on the self-immolation of **SIG1** at micromolar concentrations using fluorescence spectroscopy, and at millimolar concentrations using NMR. The results are summarized in this section and used to conduct equilibrium calculations.

All calculations were performed under the assumption that release occurs exclusively from uncomplexed **SIG1**. Accordingly, the observed slow-down ratio can be used to determine the fraction of uncomplexed **SIG1**.

*For example, given [****SIG1****]* ***=*** *10 μM, 10×CB extended the t_1/2_ from 1.5 h to 82.8 h (55.2-fold slowdown).*

*🡪This translates to an uncomplexed fraction of:*

*[uncomplexed* ***SIG1****]/[total* ***SIG1****]=1/55.2=1.81%*

*From the equilibrium relationship,*

*Ka​=[Complex]/([host][guest]),*

*Ka is estimated to be 6.01×10^5^ M^−1^.*

Equilibrium Calculations:


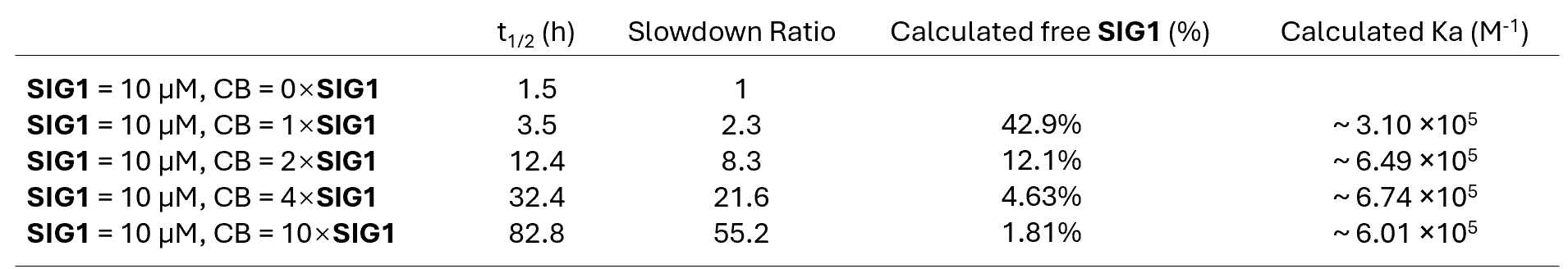


Table S1. Effect of CB-binding on the self-immolation of 10 μM **SIG1** in pH 6.5 1×MES buffer monitored by fluorescence, and the corresponding equilibrium calculations. The Ka value of **SIG1** is estimated to be 6×10^5^ M^−1^, close to but slightly lower than the ITC-measured Ka value for **SIG1_OMe_** (1.1× 10^6^ M^-1^, Figure 2c). This slight difference may be attributed to the bulkier coumarin cargo structure compared to the smaller methanol group in **SIG1_OMe_**.


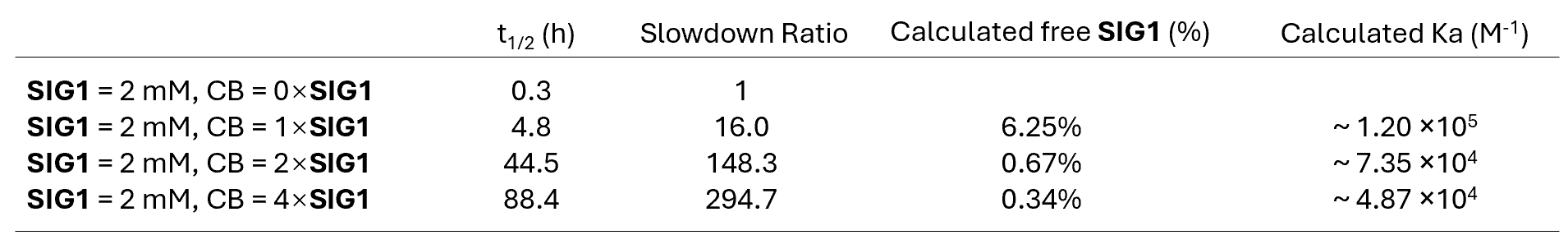


Table S2. Effect of CB-binding on the self-immolation of 2 mM **SIG1** in pH 6.5 phosphate buffer with 10% DMSO-D_6_ (for solubility) monitored by NMR, and the corresponding equilibrium calculations. Generally, the Ka values were estimated to be around 10^5^ M^−1^—the inclusion of 10% DMSO cosolvent is expected to weaken the host-guest binding. Nevertheless, at mM concentrations, binding equilibrium remains close to completion with 4×CB, even with the diminished affinity. More suppression was observed at millimolar than at micromolar levels because complexation is more complete at higher concentrations and the residual release is from uncomplexed **SIG1** molecules.

# 5. Characterizations of SIG2

**5.1. Model Self-Immolative Reaction of SIG2**

The successful release of MB from **SIG2** (16 µM in pH 6.5 MES, 37°C) was confirmed by the emergence of its characteristic absorption peak at approximately 665 nm (Figure S14b). However, the yield of released MB was limited to ~50% of the theoretical maximum, even after prolonged incubation (Figure S14b). To better understand this reaction, we conducted a model self-immolation reaction: A 3.7 mL DMSO solution of **SIG2**-TFA (39.4 mg, 37.4 µmol) was added to 370 mL of 1×MES buffer, yielding a final **SIG2** concentration of 100 µM. The mixture was incubated at 37 °C over three days and then concentrated to 50 mL under reduced pressure. The concentrated solution was extracted with DCM (150 mL) three times, and the organic phase was washed with brine (50 mL), dried over Na_2_SO_4_, filtered, and concentrated under reduced pressure. The crude was separated by column chromatography (hexanes/ethyl acetate) to yield **BP1** (13 mg, 37%) and the cyclization product (5 mg, 51%), the structures of both were confirmed by NMR, with the NMR spectrum of **BP1** shown in Figure S14c.


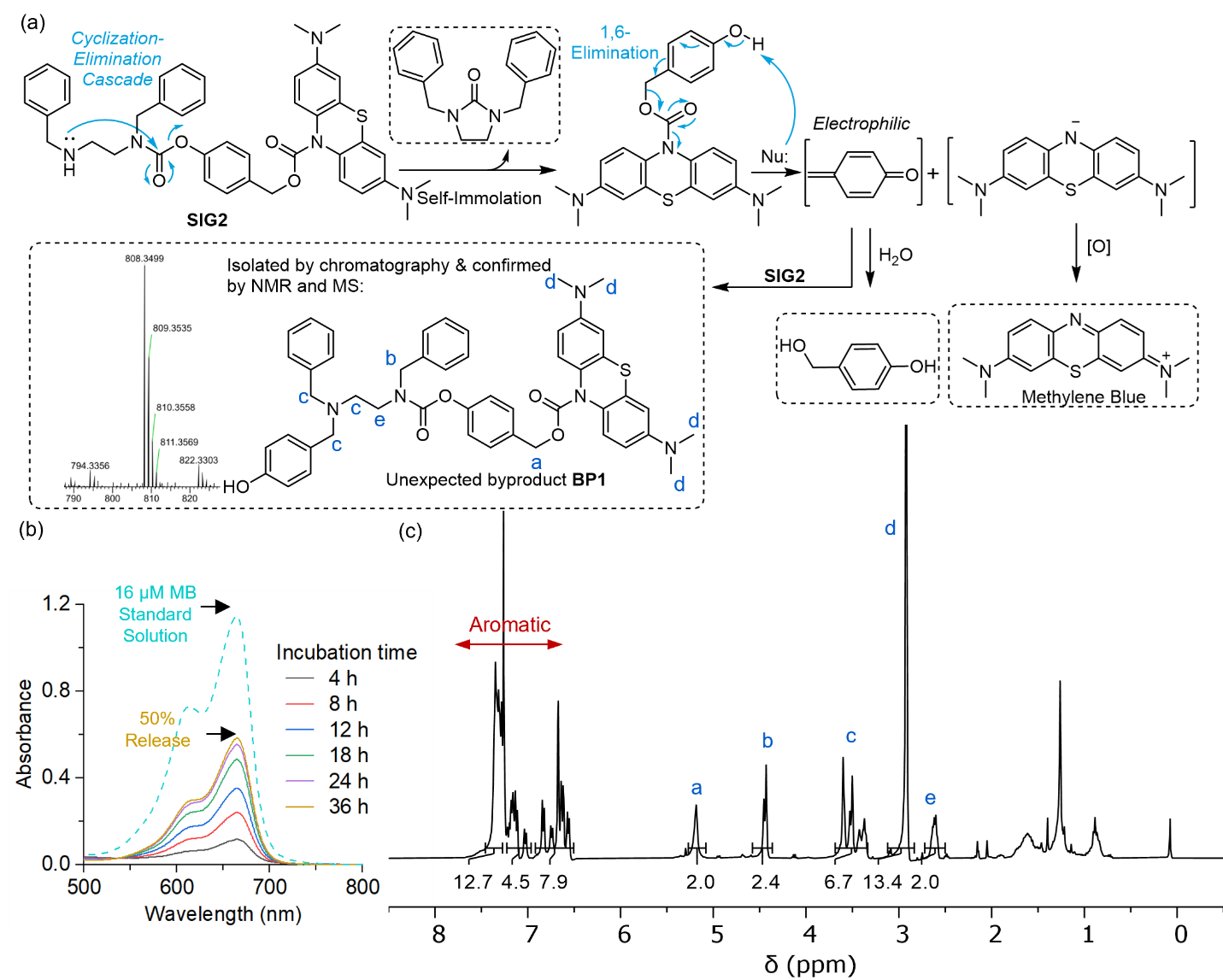


Figure S14. (a) The self-immolation mechanism for **SIG2**, and its decomposition products that were experimentally confirmed. The identity of **BP1** was confirmed by HRMS (ESI, m/z): calcd for [C48H50N5O5S]^+^ (M+H)^+^, 808.3527; found, 808.3499. (b) Time-course UV-vis spectra (solid curves) of a 16 µM solution of **SIG2** incubated at 37 °C. The dashed curve corresponds to the absorbance spectrum of a separately prepared 16 µM solution of MB. (c) ^1^H NMR spectra of an unexpected byproduct (**BP1**) from the self-immolation of **SIG2**.

**5.2. Reactivity of SIG2 as a Function of CB Stoichiometry**

The reactivity of **SIG2** was significantly inhibited by CB-Ad complexation. After 4 hours of incubation, MB release was measured at 1.88 µM for free **SIG2**, 0.43 µM for 10×CB/**SIG2**, and 2.15 µM for 10×CB/**SIG2**/20×Ad (Figure S15). This suppression was sufficient to differentiate the photodynamic cell-killing effects between the gated CB-**SIG2** complex and the active form of **SIG2**—the therapeutically effective MB concentration threshold was determined to be 2 µM (see Figure S17 and SI: **PDT Experiments**).


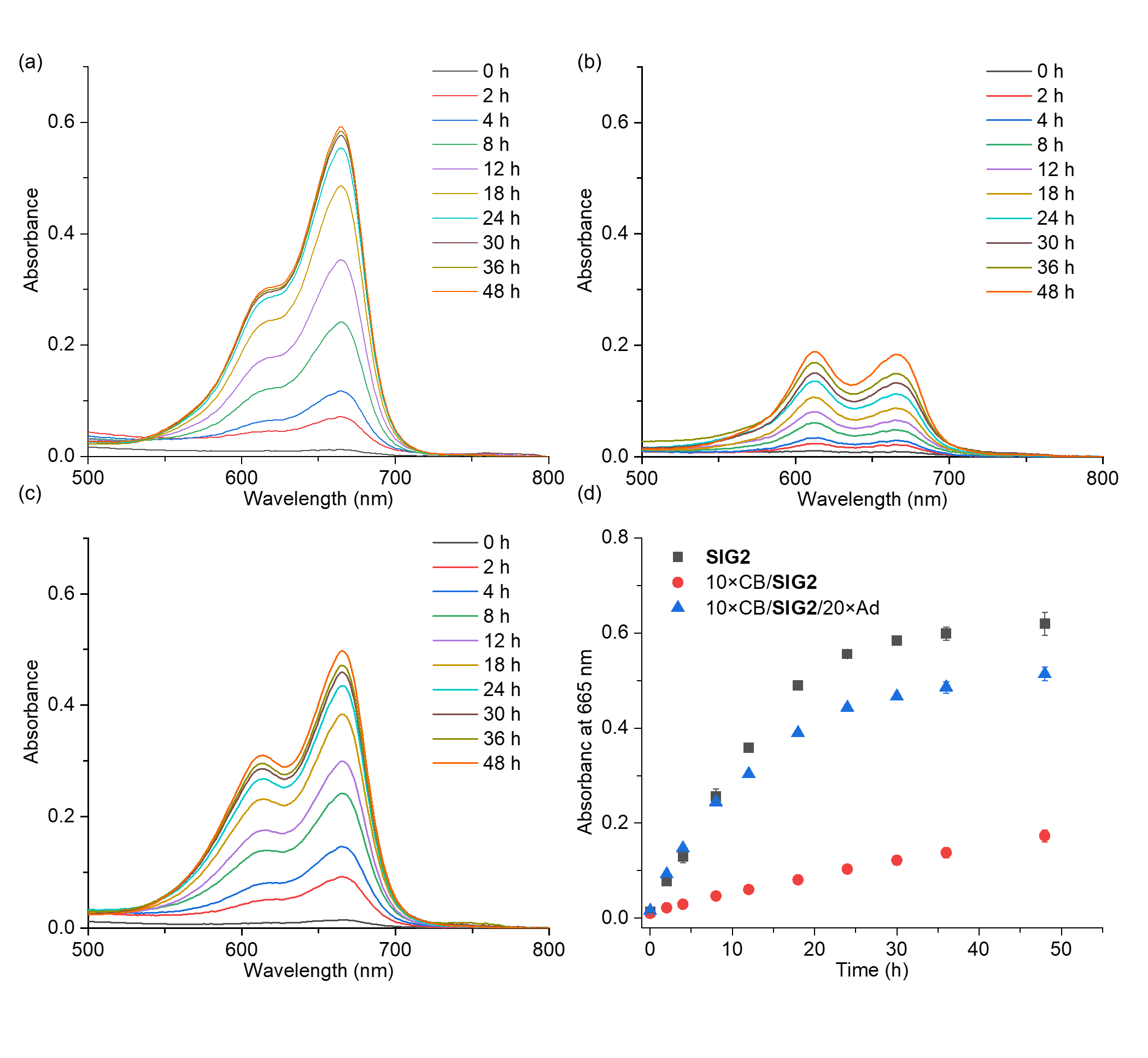


Figure S15. Time-course UV-vis spectra after baseline subtraction (with absorbance at 800 nm set to zero) of (a) **SIG2**, (b) 10×CB/**SIG2**, and (c) 10×CB/**SIG2**/20×Ad. (d) The baseline-subtracted peak absorbance at 665 nm for **SIG2**, 10×CB/**SIG2**, 10×CB/**SIG2**/20×Ad solution as a function of incubation time. [**SIG2**]_0_ = 16 µM, [CB] = 160 µM, [Ad] = 320 µM. All experiments were conducted at 37 °C in pH 6.5 MES buffer solutions. Error bars: standard deviation from three repeats.

**5.3. Quantum Yield of Singlet Oxygen Generation**

The quantum yield of singlet oxygen generation (Φ) of a given system was determined relative to MB (Φ_Δ_ = 0.52, Ref: Bull. Chem. Soc. Jpn. **1978**, *51*, 379.) using Singlet Oxygen Sensor Green (SOSG) as the fluorescent probe. For each experiment, a 2 mL solution in a quartz cuvette was irradiated at 2.2 mW cm^-2^ (> 560 nm) for 1 min before measuring its fluorescence.

The Φ_Δ_ value for each sample was calculated according to the following equation:

Φ = 0.52 × ΔI_530_(sample)/​ΔI_530_(MB)

Where ΔI_530_ is the increase in SOSG fluorescence at 530 nm.


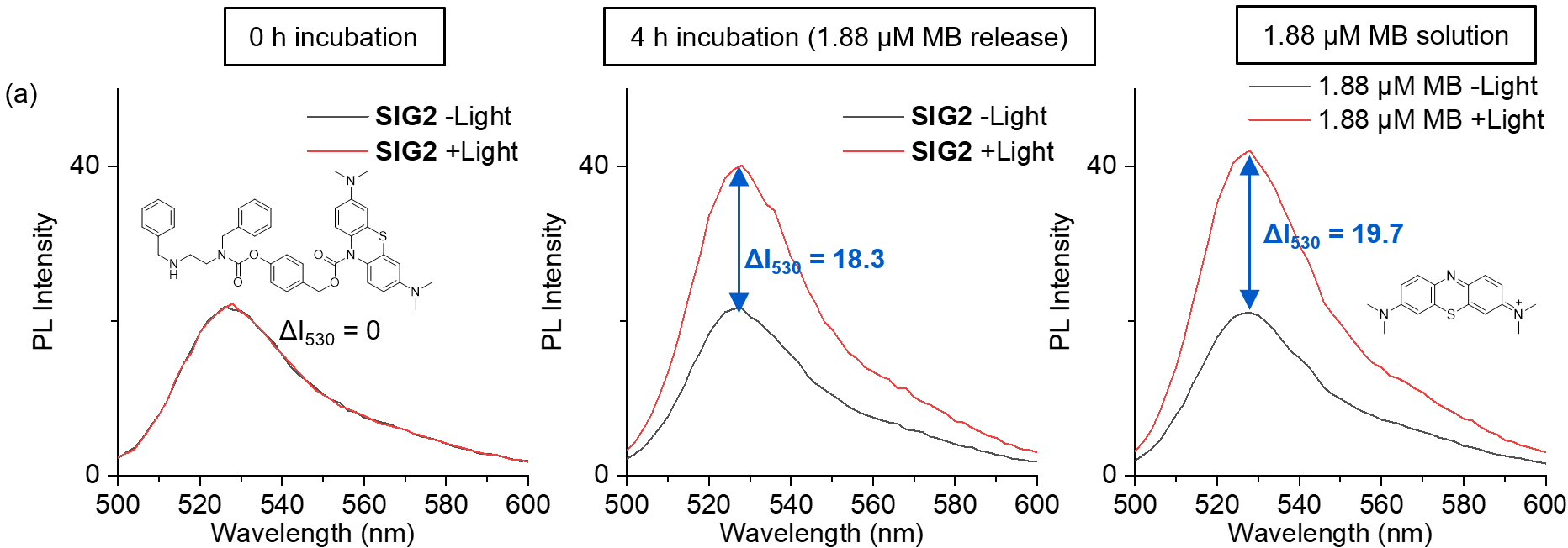


**SIG2**: Φ = 0

Release MB from **SIG2**: Φ = 0.52 × **18.3** /​ **19.7** = 0.48


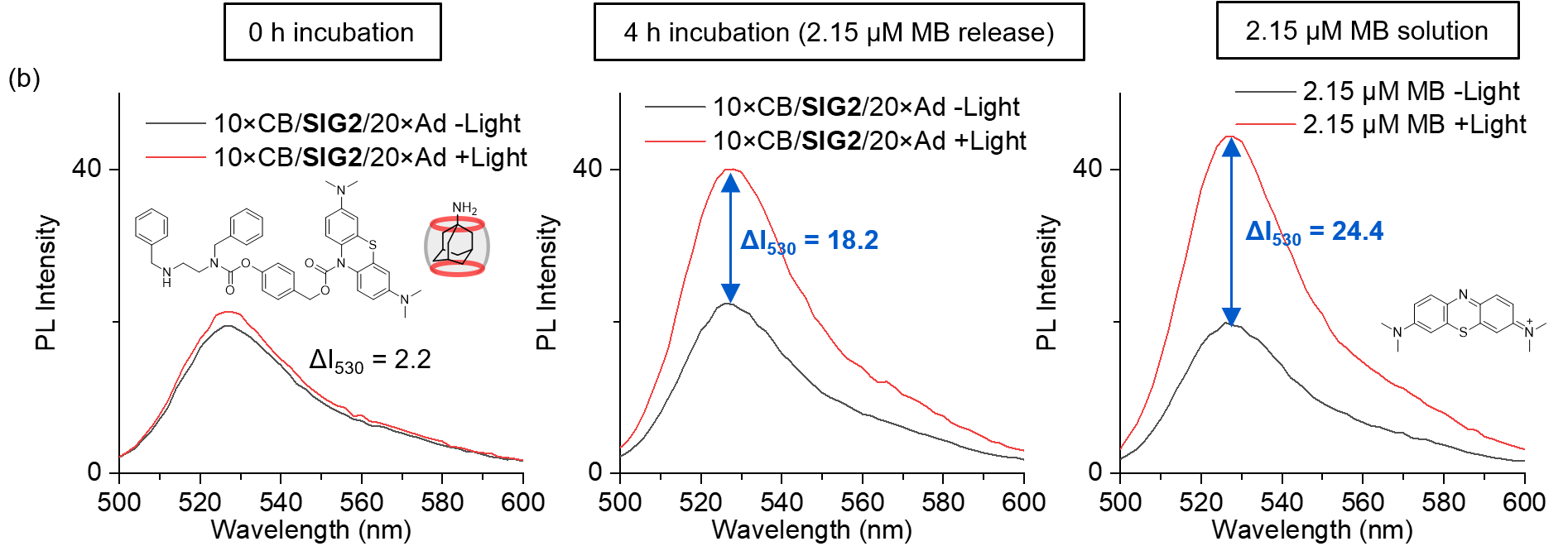


The 10×CB/**SIG2**/20×Ad system, which contains the Ad trigger, is incubated to allow for MB release. The Φ for the released MB is calculated as: Φ = 0.52 × **18.2** /​ **24.4** = 0.39

In summary, the caged MB prodrug exhibits a Φ value close to zero, consistent with the expected loss of sensitizing activity due to its chemical modification. In contrast, the Φ values of MB released from the SIG formulations closely match that of unmodified MB, demonstrating that the MB regains its intrinsic photosensitizing efficacy upon release.

# 6. PDT Experiments

**6.1. SOSG Experiments**


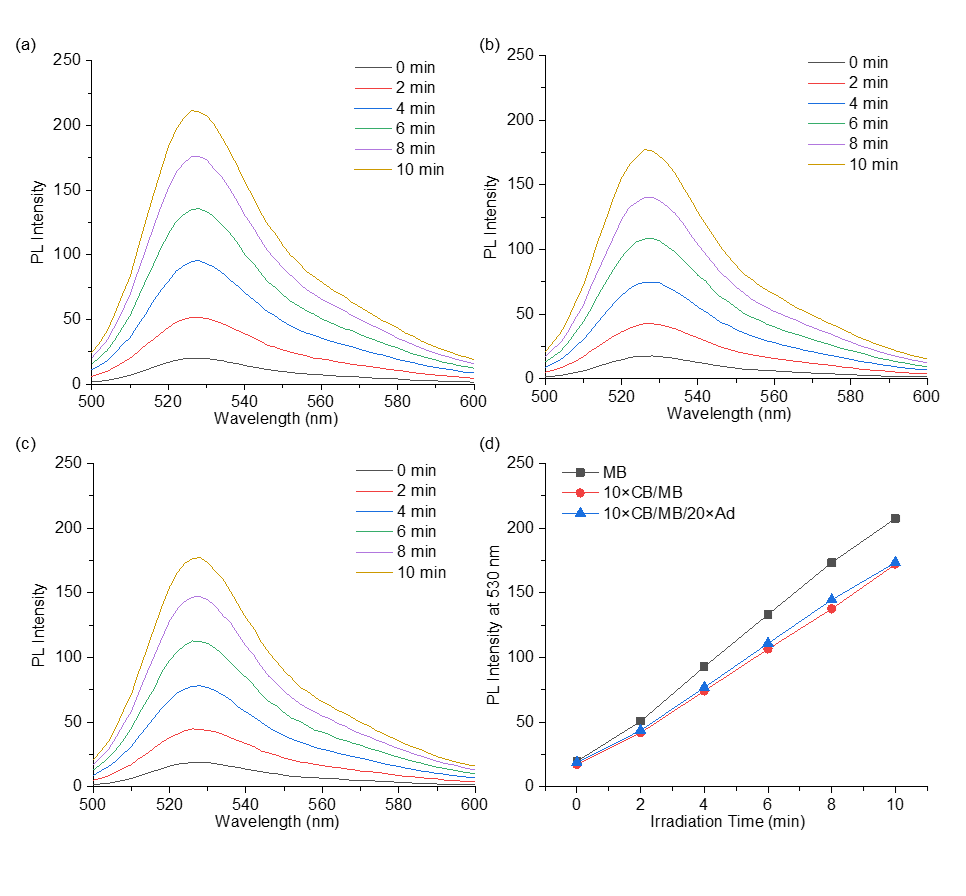


Figure S16. The photo-generation of ^1^O_2_ in (a) MB ([MB] = 1 µM), (b) 10×CB/MB ([MB] = 1 µM, [CB] = 10 µM), and (c) 10×CB/MB/20×Ad ([MB] = 1 µM, [CB] = 10 µM, [Ad] = 20 µM) systems was monitored using the SOSG fluorescence assay. (d) The photoluminescence intensities at 530 nm over irradiation time, as shown in plots a, b, and c, are summarized. All three systems showed a similar irradiation-dependent increase in fluorescence, indicating comparable efficiency in photo-generating ^1^O_2_. This suggests that the addition of CB and adamantane does not affect the PDT effect of MB. [SOSG] = 5 µM in all samples. Excitation wavelength for fluorescence measurements: λ= 488 nm.

**6.2. In-Vitro PDT Assay Using Commercial Methylene Blue**


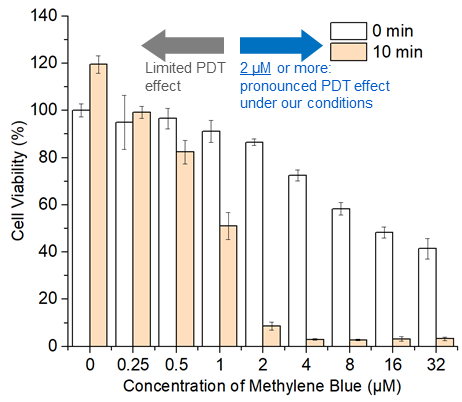


Figure S17. MTT viability assays demonstrated MB concentration-dependent PDT cytotoxicity in cells—1-2 µM or higher concentrations of MB is required for significant PDT effects. 10-min photo-irradiation alone without MB did not induce significant toxicity in *HeLa* cells (MTT viability ≈ 100%). MB at concentrations > 4 µM started to compromise the dark cell viability. Error bars: standard deviation from three repeats.

**6.3. In-Vitro PDT Assay Using the Prototype Model Prodrug SIG2**

*HeLa* cells were incubated in DMEM media, with 10% fetal bovine serum (FBS) at 37 °C and 5% CO_2_. Approximately 5,000 *HeLa* cells suspended in 100 µL of media were seeded into each well of a 96-well plate 12 hours prior to PDT experiments.


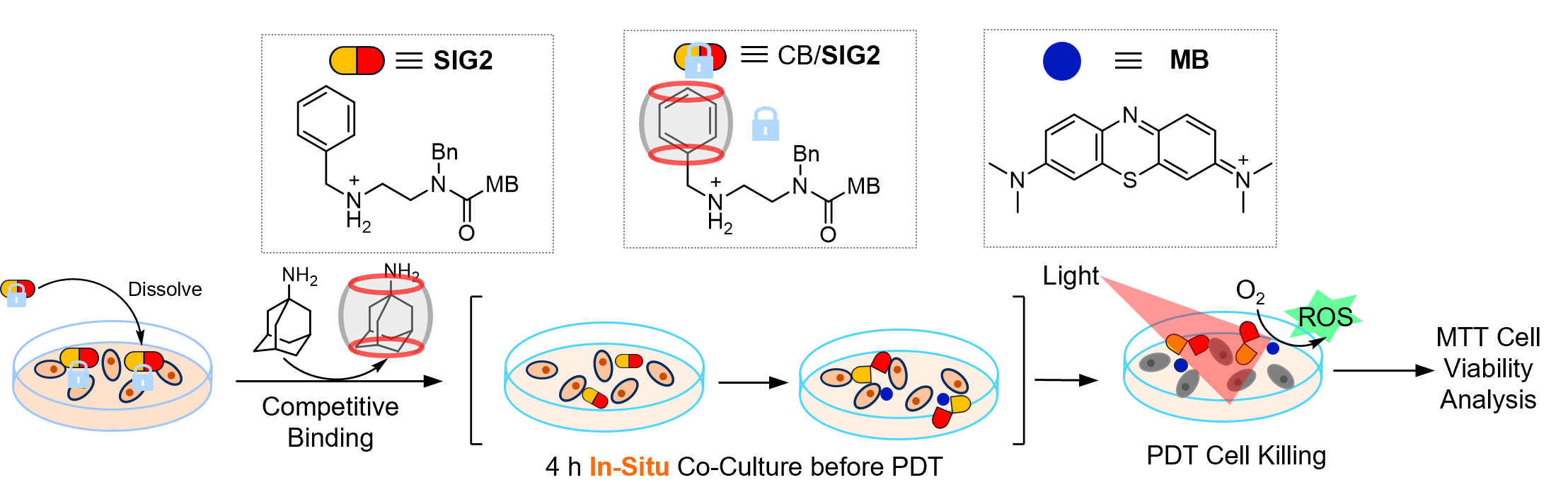


Scheme S1. Schematic illustration of the in-situ cellular experiments conducted with the CB/**SIG2**/Ad group.

**Experiments with SIG2 and CB/SIG2 Groups**: The cell culture supernatant in a 96-well plate was removed and replaced with 100 μL corresponding solutions (**SIG2** (16 µM) or CB/**SIG2** (160 µM/16 µM) solutions, depending on the specific assay). After 4 hours of co-incubation, cells were irradiated for 10 minutes. After PDT treatment, the supernatant in the 96-well plate was replaced with fresh DMEM containing 10% FBS, and cells were incubated for 24 hours at 37 °C in an atmosphere containing 5% CO_2_. Then, the supernatant was replaced with MTT (0.5 mg·mL^-1^ in DMEM media containing 10% FBS, 100 μL/well). After 4 hours of incubation at 37 °C, the supernatant was removed, followed by the addition of 100 μL DMSO to each well to dissolve the formazan product. The plates were shaken for 60 s and the absorbance at 490 nm in each well was recorded by a microplate reader.

**Experiments with CB/SIG2/Ad Groups (Scheme S1)**: The cell culture media in a 96-well plate was replaced with 100 μL of an MES solution of CB/**SIG2** (160 µM/16 µM). Subsequently, an MES solution of 1-adamantylamine (1.6 μL, 20 mM) was added to each well, resulting in a final adamantane concentration of 320 μM. Cells were incubated in this solution for 4 hours at 37 °C in an atmosphere containing 5% CO_2_, followed by 10 minutes of photo-irradiation. After PDT treatment, the cell culture media was replaced with fresh DMEM containing 10% FBS, and cells were incubated for 24 h at 37 °C in an atmosphere containing 5% CO_2_. Then, the supernatant was replaced with MTT (0.5 mg·mL^-1^ in DMEM media containing 10% FBS, 100 μL/well). After 4 hours of incubation at 37 °C, the supernatant was removed, followed by the addition of 100 μL DMSO to each well to dissolve the formazan product. The plates were shaken for 60 s and the absorbance at 490 nm in each well was recorded by a microplate reader.

# 7. Synthetic Details

Scheme S2. Synthesis of **SIG1** in the form of a TFA salt.

**Compound 1.** A flame-dried round bottom flask equipped with a stir bar was charged with tert-butyl benzyl(2-(benzylamino)ethyl)carbamate (0.1 g, 0.294 mmol) and 1 mL ethyl acetate. The solution was cooled to 0 °C in an ice bath. Coumarin chloroformate (0.140 g, 0.587 mmol) (Ref : *J. Am. Chem. Soc.* **2019**, *141*, 15018–15023) was dissolved into another 1 mL of ethyl acetate followed by the dropwise addition into round bottom flask. Potassium carbonate (0.1 g, 0.735 mmol) was dissolved into 2 mL of water and added into the mixture dropwise. After stirring the mixture for 40 min at room temperature, the mixture was extracted with DCM (50 mL). The organic fraction was washed with 10% NH_4_Cl (50 mL), water (50 mL), and brine (50 mL). The organic phase was dried over Na_2_SO_4_, filtered, and concentrated under reduced pressure. The product was separated by column chromatography (5-15 % ether/DCM) to provide the title compound as a light-yellow liquid (60 mg, 38%). R_f_ = 0.33 (DCM : ether 10 : 1). ^1^H NMR (400 MHz, Chloroform-d) δ 7.59 (t, J = 9.1 Hz, 1H), 7.44 – 6.97 (m, 12H), 6.26 (d, J = 6.8 Hz, 1H), 4.75 – 4.31 (m, 4H), 3.66 – 3.21 (m, 4H), 2.51 – 2.35 (m, 3H), 1.47 (s, 9H). ^13^C NMR (100 MHz, Chloroform-d) δ 160.71, 155.87, 154.25, 153.92, 153.82, 152.08, 138.32, 137.89, 128.89, 128.75, 128.66, 128.41, 128.23, 128.03, 127.90, 127.61, 127.40, 126.43, 125.31, 118.44, 114.33, 110.62, 110.43, 80.45, 51.71, 51.35, 45.66, 44.93, 28.54, 18.82.

HRMS (DART/Orbitrap, m/z): calcd for [C32H35N2O6]^+^ (M+H)^+^, 543.2490; found, 543.2492.

**TFA salt of SIG1**. A 20 mL vial equipped with a stir bar was charged with **1** (60 mg, 0.111 mmol) and DCM (1 mL) at room temperature. 1 mL TFA was added into the mixture dropwise. After 1 h reaction at room temperature, the product was obtained as a light-yellow liquid by removing all solvent under reduced pressure (>99%, 62 mg). ^1^H NMR (400 MHz, Methanol-d_4_) δ 7.67 (d, J = 8.6 Hz, 1H), 7.45 – 7.20 (m, 10H), 7.18 – 7.01 (m, 2H), 6.19 (d, J = 1.5 Hz, 1H), 4.69 – 4.46 (m, 2H), 4.16 (s, 2H), 3.75 – 3.57 (m, 2H), 3.34 – 3.06 (m, 4H), 2.36 (d, J = 1.3 Hz, 3H). ^13^C NMR (100 MHz, Methanol-d_4_) δ 162.65, 156.33, 155.20, 155.07, 154.99, 138.03, 137.89, 132.33, 131.05, 130.82, 130.37, 130.11, 129.11, 128.57, 127.13, 119.68, 119.43, 118.99, 114.79, 111.39, 111.35, 111.22, 52.60, 52.44, 46.70, 45.04, 18.71.

HRMS (DART/Orbitrap, m/z): calcd for [C27H27O4N2]^+^ (M)^+^, 443.1965; found, 443.1963.

Scheme S3. Synthesis of **SIG0** in the form of a TFA salt.

**Compound 2.** A round bottom flask with a stir bar was charged with tert-butyl (2-aminoethyl)(benzyl)carbamate (0.2 g, 0.8 mmol) and 1 mL ethyl acetate. The solution was cooled to 0 °C in an ice bath. Coumarin chloroformate (0.381 g, 1.6 mmol) was dissolved into another 1 mL of ethyl acetate followed by the dropwise addition into a round bottom flask. Potassium carbonate (0.28 g, 2 mmol) was dissolved into 2 mL water and added into the mixture dropwise. After stirring the mixture for 40 min at room temperature, the mixture was extracted with DCM (50 mL). The organic fraction was washed with 10% NH_4_Cl (50 mL), water (50 mL), and brine (50 mL). The organic phase was dried over Na_2_SO_4_, filtered, and concentrated under reduced pressure. The product was separated by column chromatography (5-25 % ether/DCM) to provide the title compound as a light-yellow liquid (220 mg, 61%). R_f_ = 0.18 (DCM : ether 10 : 1). ^1^H NMR (400 MHz, Chloroform-d) δ 7.57 (d, J = 8.4 Hz, 1H), 7.47 – 7.20 (m, 6H), 7.18 – 7.07 (m, 2H), 6.24 (d, J = 1.3 Hz, 1H), 4.48 (s, 2H), 3.75 – 3.23 (m, 4H), 2.42 (d, J = 1.3 Hz, 3H), 1.52 (s, 9H). ^13^C NMR (100 MHz, CDCl_3_) δ 160.80, 154.19, 153.78, 153.53, 152.18, 151.7, 137.99, 128.76, 127.87, 127.56, 127.35, 126.73, 125.27, 118.05, 117.27, 114.14, 110.13, 80.76, 51.22, 45.77, 40.54, 28.46, 18.77.

HRMS (DART/Orbitrap, m/z): calcd for [C25H32N3O6]^+^ (M+NH_4_)^+^, 470.2286; found, 470.2288.

**TFA salt of SIG0.** A 20 mL vial equipped with a stir bar was charged with **2** (0.1 g, 0.8 mmol) and DCM (2 mL) at room temperature. 2 mL Trifluoroacetic acid (TFA) was added into the mixture dropwise. After 1 h reaction at room temperature, the product was obtained as a light-yellow liquid by removing all solvent under reduced pressure (>99%, 106 mg). ^1^H NMR (400 MHz, Chloroform-d) δ 7.52 (d, J = 9.4 Hz, 1H), 7.45 – 7.30 (m, 5H), 7.09 – 7.02 (m, 2H), 6.68 (t, J = 5.9 Hz, 1H), 6.21 (d, J = 1.3 Hz, 1H), 4.22 – 3.84 (m, 4H), 3.76 – 3.42 (m, 2H), 3.34 – 3.00 (m, 2H), 2.39 (d, J = 1.3 Hz, 3H). ^13^C NMR (100 MHz, Chloroform-d) δ 161.04, 154.45, 154.05, 153.42, 152.49, 130.11, 130.07, 129.84, 129.50, 125.42, 118.13, 117.55, 114.24, 110.26, 51.74, 46.96, 37.87, 18.83.

HRMS (DART/Orbitrap, m/z): calcd for [C20H21O4N2]^+^ (M)^+^, 353.1496; found, 353.1471.

Scheme S4. Synthesis of **SIG1_OMe_** in the form of a TFA salt.

**Compound 3.** A round bottom flask equipped with a stir bar was charged with tert-butyl benzyl(2-(benzylamino)ethyl)carbamate (1 g, 2.938 mmol), 4-nitrophenyl chloroformate (0.533 g, 2.643 mmol), and anhydrous THF (20 mL). The mixture was cooled to 0 °C in an ice bath before adding triethylamine (TEA, 0.297 g, 2.97 mmol) dropwise. After overnight reaction at room temperature, the mixture was extracted with EtOAc (150 mL). The organic fraction was washed with 10% NH_4_Cl (150 mL), water (150 mL), and brine (150 mL). The organic phase was dried over Na_2_SO_4_, filtered, and concentrated under reduced pressure. The product was separated by column chromatography (5-15 % EtOAc/hexanes) to provide the title compound as a light-yellow oil (1.06 g, 72%). R_f_ = 0.50 (hexanes : EtOAc 5 : 1). ^1^H NMR (400 MHz, Chloroform-d) δ 8.25 (t, J = 9.4 Hz, 2H), 7.46 – 7.06 (m, 12H), 4.79 – 4.30 (m, 4H), 3.61 – 3.33 (m, 4H), 1.57 – 1.36 (m, 9H). ^13^C NMR (100 MHz, Chloroform-d) δ 157.02, 154.67, 154.60, 145.74, 137.76, 137.68, 128.98, 128.82, 128.80, 128.73, 128.44, 128.28, 128.07, 127.69, 127.50, 127.36, 125.54, 125.24, 122.34, 79.70, 53.56, 51.45, 46.52. 46.45, 28.58.

HRMS (DART/Orbitrap, m/z): calcd for [C28H32N3O6]^+^ (M+H)^+^, 506.2286; found, 506.2285.

**Compound 4**. A 20 mL vial equipped with a stir bar was charged with NaH (60 % dispersion in mineral oil, 21 mg, 0.52 mmol) and anhydrous DMF (2 mL) at room temperature. The methanol (21 mg, 0.59 mmol) was added to the mixture dropwise. After stirring at room temperature for 30 min, compound **3** (0.2g, 0.396 mmol) dissolved in anhydrous DMF (1 mL) was dropwise added into the mixture. After reacting at room temperature for 2 h, 1 mL water was added in to quench the reaction. The mixture was extracted with EtOAc (150 mL). The organic fraction washed with brine (150 mL). The organic fraction was dried over Na_2_SO_4_, filtered, and concentrated under reduced pressure to yield a crude mixture. The crude product was purified by column chromatography (5–25% EtOAC/hexanes) to provide the title compound as a colorless liquid (70 mg, 45%). Rf = 0.3 (hexanes : EtOAc 10 : 1). ^1^H NMR (400 MHz, Chloroform-d) δ 7.52 – 6.92 (m, 10H), 4.43 (dd, J = 21.3, 14.2 Hz, 4H), 3.73 (s, 3H), 3.31 (t, J = 37.6 Hz, 4H), 1.47 (d, J = 12.9 Hz, 9H). ^13^C NMR (100 MHz, Chloroform-d) δ 157.27, 155.86, 137.74, 137.72, 128.72, 128.67, 128.27, 128.25, 128.23, 128.07, 127.91, 127.45, 79.69, 52.91, 51.12, 50.49, 44.38, 44.08, 28.54.

HRMS (DART/Orbitrap) m/z: [M+H]^+^ Calcd for C23H31N2O4 399.2278; Found 399.2278.

**TFA salt of SIG1_OMe_.** A 20 mL vial equipped with a stir bar was charged with **4** (58 mg, 0.147 mmol) and CHCl_3_ (2 mL) at room temperature. 2 mL TFA was added into the mixture dropwise. After 1 h reaction at room temperature, the product was obtained as a white solid by removing all solvent under reduced pressure. (>99%, 57 mg). ^1^H NMR (400 MHz, Methanol-d_4_) δ 7.46 (s, 5H), 7.38 – 7.18 (m, 5H), 4.52 (s, 2H), 4.21 (s, 2H), 3.78 (s, 3H), 3.59 (d, J = 7.8 Hz, 2H), 3.16 (s, 2H). ^13^C NMR (100 MHz, Methanol-d_4_) δ 138.39, 130.98, 130.79, 130.34, 129.89, 128.86, 128.61, 128.54, 128.38, 53.85, 52.43, 51.73, 47.89, 45.33.

HRMS (DART/Orbitrap) m/z: [M]^+^ Calcd for C18H23N2O2 299.1754; Found 299.1753.

Scheme S5. Synthesis of **SIG1_NBD_** in the form of a TFA salt.

**Compound 5**. A round bottom flask equipped with a stir bar was charged with 2-[Methyl(7-nitro-2,1,3-benzoxadiazol-4-yl)amino]ethanol (Ref: *Polym. Chem.*, **2015**, 6, 4966-4971) (0.1 g, 0.418 mmol), 4-nitrophenyl chloroformate (0.211 g, 0.418 mmol), and anhydrous THF (20 mL). The mixture was cooled to 0 °C in an ice bath before adding DIPEA (0.054 g, 0.418 mmol) dropwise. After overnight reaction at room temperature, the mixture was extracted with EtOAc (150 mL). The organic fraction was washed with 10% NH_4_Cl (150 mL), water (150 mL), and brine (150 mL). The organic phase was dried over Na_2_SO_4_, filtered, and concentrated under reduced pressure. The product was separated by column chromatography (1-10 % MeOH/CHCl_3_) to provide the title compound as a light-yellow oil (0.056 g, 33%). R_f_ = 0.30 (MeOH : CHCl_3_ 20 : 1). ^1^H NMR (400 MHz, Acetone-d_6_) δ 8.50 (d, J = 9.0 Hz, 1H), 8.33 – 8.25 (m, 2H), 7.49 – 7.40 (m, 2H), 6.52 (d, J = 9.0 Hz, 1H), 4.76 (dd, J = 5.8, 4.9 Hz, 2H), 4.66 (t, J = 5.1 Hz, 2H), 3.63 (s, 3H). ^13^C NMR (101 MHz, Acetone-d_6_) δ 156.47, 153.03, 146.78, 146.50, 145.87, 145.83, 136.42, 126.08, 123.07, 122.10, 103.25, 67.51, 54.45, 42.22.

HRMS (DART/Orbitrap) m/z: [M+H]^+^ Calcd for C16H14N5O8 404.0837; Found 404.0837.

**Compound 6**. A round bottom flask equipped with a stir bar was charged with compound **5** (0.05 g, 0.124 mmol), tert-butyl benzyl(2-(benzylamino)ethyl)carbamate (0.1 g, 0.298 mmol), and anhydrous THF (5 mL). The mixture was cooled to 0 °C in an ice bath before adding N,N-Diisopropylethylamine (DIPEA, 0.031 g, 0.24 mmol) dropwise. After overnight reaction at 60 °C, the mixture was extracted with EtOAc (150 mL). The organic fraction was washed with 10% NH_4_Cl (150 mL), water (150 mL), and brine (150 mL). The organic phase was dried over Na_2_SO_4_, filtered, and concentrated under reduced pressure. The product was separated by column chromatography (10-40 % acetone/hexanes) to provide the title compound as a yellow solid (0.065 g, 89%). Rf = 0.3 (hexanes : acetone 3 : 1). ^1^H NMR (400 MHz, Chloroform-d) δ 8.40 – 8.12 (m, 1H), 7.51 – 6.71 (m, 10H), 6.19 – 5.86 (m, 1H), 4.73 – 3.99 (m, 8H), 3.63 – 2.90 (m, 7H), 1.44 (d, J = 6.8 Hz, 9H). ^13^C NMR (100 MHz, Chloroform-d) δ 156.05, 154.76, 145.40, 144.57, 140.25, 135.32, 135.16, 128.70, 128.62, 128.57, 128.45, 128.12, 127.82, 127.54, 127.39, 127.24, 127.02, 126.10, 122.98, 101.79, 101.68, 54.57, 53.66, 51.17, 47.38, 46.63, 44.06, 28.46.

HRMS (DART/Orbitrap) m/z: [M+H]^+^ Calcd for C31H37N6O7 605.2718; Found 605.2715.

**TFA salt of SIG1_NBD_.** A 20 mL vial equipped with a stir bar was charged with **6** (40 mg, 0.066 mmol) and CHCl_3_ (2 mL) at room temperature. 2 mL TFA was added into the mixture dropwise. After 1 h reaction at room temperature, the product was obtained as a yellow solid by removing all solvent under reduced pressure. (>99%, 39 mg). ^1^H NMR (400 MHz, Methanol-d_4_) δ 8.28 – 7.99 (m, 1H), 7.39 – 7.16 (m, 5H), 7.11 – 6.51 (m, 5H), 6.25 – 5.99 (m, 1H), 4.46 – 4.15 (m, 4H), 4.13 – 3.94 (m, 4H), 3.40 – 3.15 (m, 6H), 3.01 – 2.83 (m, 2H). ^13^C NMR (100 MHz, Methanol-d_4_) δ 161.98, 158.59, 147.53, 145.99, 137.59, 136.91, 132.33, 130.99, 130.90, 130.53, 130.29, 129.65, 128.39, 127.25, 123.09, 103.42, 64.72, 54.60, 52.70, 52.38, 51.37, 46.95, 44.00.

HRMS (DART/Orbitrap) m/z: [M]^+^ Calcd for C26H29N6O5 505.2194; Found 505.2193.

Scheme S6. Synthesis of **SIG1_Aniline_** in the form of a TFA salt.

**Compound 7**. A round bottom flask equipped with a stir bar was charged with tert-butyl benzyl(2-(benzylamino)ethyl)carbamate (0.2 g, 0.587 mmol), 4-nitrophenyl isocyanate (0.116 g, 0.705 mmol), DMAP (0.086 g, 0.705 mmol), and anhydrous DMF (2 mL). After reacting at 60 °C overnight, the mixture was extracted with ether (150 mL). The organic fraction was washed with 10% NH_4_Cl (50 mL), water (50 mL), and brine (50 mL). The organic fraction was dried over Na_2_SO_4_, filtered, and concentrated under reduced pressure to yield a crude mixture. The crude product was purified by column chromatography (10–30% acetone/hexanes) to provide the title compound as a yellow solid (0.196 g, 66%). Rf = 0.30 (hexanes : acetone 4 : 1). ^1^H NMR (400 MHz, Chloroform-d) δ 9.38 (s, 1H), 8.22 – 8.12 (m, 2H), 7.93 (d, J = 8.8 Hz, 2H), 7.40 – 7.23 (m, 6H), 7.14 (ddd, J = 29.2, 7.3, 2.2 Hz, 4H), 4.47 (s, 2H), 4.29 (s, 2H), 3.25 – 2.86 (m, 4H), 1.54 (s, 9H). ^13^C NMR (100 MHz, Chloroform-d) δ 156.60, 154.99, 147.26, 142.03, 138.07, 137.63, 128.95, 128.82, 128.45, 128.30, 128.23, 128.05, 127.87, 127.82, 125.02, 118.41, 81.84, 52.61, 51.09, 47.03, 44.63, 28.59.

HRMS (DART/Orbitrap) m/z: [M+H]^+^ Calcd for C28H33N4O5 505.2445; Found 505.2443.

**TFA salt of SIG1_Aniline_.** A 20 mL vial equipped with a stir bar was charged with **7** (70 mg, 0.139 mmol) and CHCl_3_ (2 mL) at room temperature. 2 mL TFA was added into the mixture dropwise. After 1 h reaction at room temperature, the product was obtained as a yellow liquid by removing all solvent under reduced pressure. (>99%, 70 mg). ^1^H NMR (400 MHz, Methanol-d_4_) δ 8.20 – 8.12 (m, 2H), 7.75 – 7.67 (m, 2H), 7.53 – 7.42 (m, 5H), 7.41 – 7.34 (m, 2H), 7.35 – 7.21 (m, 3H), 4.75 (s, 2H), 4.24 (s, 2H), 3.72 (t, J = 6.0 Hz, 2H), 3.22 (t, J = 6.0 Hz, 2H). ^13^C NMR (100 MHz, Methanol-d_4_) δ 158.18, 147.32, 143.95, 137.78, 132.42, 130.97, 130.75, 130.33, 130.09, 128.95, 128.13, 125.49, 120.82, 118.36, 52.38, 52.11, 47.39, 44.96.

HRMS (DART/Orbitrap) m/z: [M]^+^ Calcd for C23H25N4O3 405.1921; Found 405.1918.

Scheme S7. Synthesis of **SIG1_SCO_** in the form of a TFA salt.

**Compound 8**. A flame-dried round bottom flask equipped with a stir bar was charged with triphosgene (0.071 g, 0.238 mmol) and 2 mL anhydrous THF. The solution was cooled to 0 °C in an ice bath. Scopoletin (0.137 g, 0.714 mmol) was dissolved into another 1 mL of anhydrous THF followed by the dropwise addition into round bottom flask. Et_3_N (0.072 g, 0.714 mmol) was added into round bottom flask dropwise. After stirring the mixture for 30 min at room temperature, tert-butyl benzyl(2-(benzylamino)ethyl)carbamate (0.316 g, 0.928 mmol) was dissolved into 1 mL of anhydrous THF followed by the dropwise addition into round bottom flask. Et_3_N (0.068 g, 0.857 mmol) was added into round bottom flask dropwise. After stirring the mixture for 1 hours at room temperature, the mixture was extracted with DCM (50 mL). The organic fraction was washed with 10% NH_4_Cl (50 mL), water (50 mL), and brine (50 mL). The organic phase was dried over Na_2_SO_4_, filtered, and concentrated under reduced pressure. The product was separated as a light-yellow liquid by column chromatography (5-25 % ether/DCM) to provide the title compound as a light-yellow oil (130 mg, 23%). Rf = 0.3 (DCM : ether 5 : 1). ^1^H NMR (400 MHz, Chloroform-d) δ 7.64 (dd, J = 9.6, 4.8 Hz, 1H), 7.46 – 7.05 (m, 11H), 6.95 (d, J = 2.5 Hz, 1H), 6.38 (dd, J = 9.6, 5.3 Hz, 1H), 4.82 – 4.38 (m, 4H), 3.88 – 3.70 (m, 3H), 3.62 – 3.20 (m, 4H), 1.47 (d, J = 12.0 Hz, 9H). ^13^C NMR (101 MHz, Chloroform-d) δ 160.79, 155.79, 154.07, 149.02, 148.91, 148.44, 148.41, 137.77, 137.72, 128.74, 128.62, 128.25, 128.00, 127.92, 127.84, 127.68, 127.45, 116.60, 116.24, 112.36, 109.41, 79.69, 56.40, 51.60, 50.60, 45.16, 44.85, 28.53.

HRMS (DART/Orbitrap) m/z: [M+H]^+^ Calcd for C32H35N2O7 559.2439; Found 559.2435.

**TFA salt of SIG1_SCO_.** A 20 mL vial equipped with a stir bar was charged with **8** (70 mg, 0.125 mmol) and DCM (2 mL) at room temperature. 2 mL TFA was added into the mixture dropwise. After 1 h reaction at room temperature, the product was obtained as a white solid by removing all solvent under reduced pressure. (>99%, 70 mg). ^1^H NMR (400 MHz, Methanol-d_4_) δ 7.95 (d, J = 9.6 Hz, 1H), 7.53 – 7.28 (m, 11H), 7.23 (s, 1H), 6.43 (d, J = 9.6 Hz, 1H), 4.68 (d, J = 53.6 Hz, 2H), 4.24 (d, J = 5.6 Hz, 2H), 3.96 – 3.60 (m, 4H), 3.39 – 3.20 (m, 5H). ^13^C NMR (100 MHz, Methanol-d_4_) δ 161.31, 159.91, 149.36, 147.99, 143.80, 143.07, 137.75, 136.44, 130.87, 129.68, 129.58, 129.44, 128.97, 128.56, 127.74, 117.16, 115.45, 109.84, 57.23, 55.54, 51.01, 47.90, 43.28.

HRMS (DART/Orbitrap) m/z: [M]^+^ Calcd for C27H27N2O5 459.1914; Found 459.1914.

Scheme S8. Synthesis of **SIG1_4NP_** in the form of a TFA salt.

**TFA salt of SIG1_4NP_.** A 20 mL vial equipped with a stir bar was charged with **3** (105 mg, 0.208 mmol) and CHCl_3_ (2 mL) at room temperature. 2 mL TFA was added into the mixture dropwise. After 1 h reaction at room temperature, the product was obtained as a white solid by removing all solvent under reduced pressure. (>99%, 104 mg). ^1^H NMR (400 MHz, Methanol-d_4_) δ 8.36 – 8.23 (m, 2H), 7.56 – 7.22 (m, 12H), 4.68 (m, 2H), 4.25 (s, 2H), 3.76 (m, 2H), 3.29 (m, 2H). ^13^C NMR (101 MHz, Methanol-d_4_) δ 161.53, 157.28, 155.96, 146.64, 137.92, 132.27, 131.01, 130.79, 130.33, 129.09, 128.52, 126.06, 123.91, 52.45, 52.24, 46.60, 45.05.

HRMS (DART/Orbitrap) m/z: [M]^+^ Calcd for C23H24N3O4 406.1761; Found 406.1760.

Scheme S9. Synthesis of **SIG1_4-OHT_** in the form of a TFA salt.

**Compound 9**. A 20 mL vial equipped with a stir bar was charged with NaH (60 % dispersion in mineral oil, 12 mg, 0.31 mmol) and anhydrous THF (2 mL) at room temperature. The (E/Z)-4-hydroxy Tamoxifen (4-OHT, 100 mg, 0.26 mmol) was added to the mixture dropwise. After stirring at room temperature for 30 min, compound **3** (0.13g, 0.26 mmol) dissolved in anhydrous THF (1 mL) was dropwise added into the mixture. After reacting at 60 ^o^C overnight, 1 mL water was added in to quench the reaction. The mixture was extracted with EtOAc (50 mL). The organic fraction washed with brine (50 mL). The organic fraction was dried over Na_2_SO_4_, filtered, and concentrated under reduced pressure to yield a crude mixture. The crude product was purified by column chromatography (1–5% MeOH/CHCl_3_) to provide the title compound as a white solid (120 mg, 62%). Rf = 0.3 (CHCl3 : MeOH 20 : 1). ^1^H NMR (400 MHz, Chloroform-d) δ7.43–6.51 (m, 24H),4.87–4.29 (m, 4H),4.17–3.87 (m, 2H),3.64–3.17 (m, 4H),2.81–2.61 (m, 2H),2.57–2.43 (m, 2H),2.43–2.24 (m, 6H),1.56–1.38 (m, 9H),1.00–0.87 (m, 3H). ^13^C NMR (100 MHz, Chloroform-d) δ 157.64, 156.83, 155.79, 155.12, 154.96, 142.36, 142.07, 141.65, 140.93, 137.48, 135.39, 131.94, 131.71, 130.67, 130.38, 129.69, 128.73, 128.61, 127.93, 127.89, 127.35, 126.15, 126.05, 121.36, 121.26, 120.52, 120.38, 114.14, 113.41, 80.17, 65.93, 65.69, 58.36, 58.30, 45.94, 45.90, 29.15, 29.01, 28.45, 13.60.

HRMS (DART/Orbitrap) m/z: [M+H]^+^ Calcd for C48H56N3O5 754.4214; Found 754.4199.

**TFA salt of SIG1_4-OHT_.** A 20 mL vial equipped with a stir bar was charged with **9** (35 mg, 0.046 mmol) and CHCl_3_ (2 mL) at room temperature. 2 mL TFA was added into the mixture dropwise. After 1 h reaction at room temperature, the product was obtained as a white solid by removing all solvent under reduced pressure. (>99%, 35 mg). ^1^H NMR (400 MHz, Methanol-d_4_) δ7.59–6.60 (m, 23H),4.79–4.49 (m, 2H),4.42–4.14 (m, 4H),3.86–3.44 (m, 4H),3.30–3.16 (m, 2H),3.04–2.87 (m, 6H),2.56–2.41 (m, 2H),0.96–0.86 (m, 3H).^13^C NMR (100 MHz, Methanol-d_4_) δ 156.65, 155.80, 142.35, 142.06, 137.35, 137.29, 136.56, 131.74, 131.32, 130.43, 130.03, 129.64, 129.60, 129.42, 129.37, 128.93, 128.58, 127.64, 127.58, 127.21, 126.01, 125.94, 121.17, 120.23, 114.15, 113.30, 61.74, 61.49, 56.35, 56.31, 51.00, 42.46, 42.41, 28.57, 28.51, 12.36, 12.34.

HRMS (DART/Orbitrap) m/z: [M+H]^+^ Calcd for C43H49N3O3 655.3768; Found 655.3750.

Scheme S10. Synthesis of **SIG2** in the form of a TFA salt.

**Compound 10.** A 20 mL vial equipped with a stir bar was charged with NaH (60 % dispersion in mineral oil, 48 mg, 1.19 mmol) and anhydrous DMF (2 mL) at room temperature. The 4-(((tert-butyldimethylsilyl)oxy)methyl)phenol (283 mg, 1.19 mmol) dissolved in anhydrous DMF was added to the mixture dropwise. After stirring at room temperature for 30 min, compound **3** (0.5g, 0.989 mmol) dissolved in anhydrous DMF (4 mL) was dropwise added into the mixture. After reacting at 110 °C for 2 h, 1 mL water was added in to quench the reaction. The mixture was extracted with EtOAc (150 mL). The organic fraction washed with brine (150 mL). The organic fraction was dried over Na_2_SO_4_, filtered, and concentrated under reduced pressure to yield a crude mixture. The crude product was purified by column chromatography (5–25% EtOAC/hexanes) to provide the title compound as a colorless liquid (0.38 g, 64%). R_f_ = 0.15 (hexanes : EtOAc 10 : 1). ^1^H NMR (400 MHz, Chloroform-d) δ 7.40 – 7.13 (m, 12H), 7.10 – 6.99 (m, 2H), 4.81 – 4.37 (m, 6H), 3.56 – 3.27 (m, 4H), 1.53 – 1.42 (m, 9H), 0.99 – 0.88 (m, 9H), 0.14 – 0.05 (m, 6H). ^13^C NMR (100 MHz, Chloroform-d) δ 155.91, 155.34, 150.25, 138.70, 138.59, 133.49, 128.82, 128.72, 128.47, 128.29, 128.19, 128.13, 128.05, 127.90, 127.87, 127.52, 127.35, 127.04, 121.65, 121.50, 80.15, 64.62, 50.51, 50.43, 44.71, 44.66, 28.57, 26.07, 18.53, -5.11. HRMS (DART/Orbitrap, m/z): calcd for [C35H49N2O5Si]^+^ (M+H)^+^, 605.3405; found, 605.3408.

**Compound 11.** A flame-dried round bottom flask equipped with a stir bar was charged with **10** (0.38 g, 0.628 mmol) and 4 mL anhydrous THF. The mixture was cooled to 0 °C in an ice bath, followed by the dropwise addition of TBAF (1 M in THF, 0.754 mL, 0.754 mmol). The stirred reaction was allowed to warm up to room temperature. After one hour, the reaction was quenched with NH_4_Cl (10 mL) and extracted with EtOAc (100 mL). The organic fraction was washed with water (50 mL) and brine (50 mL). The organic fraction was dried over Na_2_SO_4_, filtered, and concentrated under reduced pressure. The crude product was purified by column chromatography (15–35% EtOAc/hexanes) to yield the title compound as a white solid (204 mg, 66%). R_f_ = 0.23 (hexanes : EtOAc 5 : 1). ^1^H NMR (400 MHz, Chloroform-d) δ 7.41 – 7.14 (m, 12H), 7.15 – 7.04 (m, 2H), 4.76 – 4.34 (m, 6H), 3.56 – 3.25 (m, 4H), 1.52 – 1.38 (m, 9H). ^13^C NMR (100 MHz, Chloroform-d) δ 155.93, 155.24, 150.88, 138.20, 137.10, 136.95, 128.85, 128.74, 128.50, 128.46, 128.35, 128.28, 128.15, 128.11, 127.84, 127.77, 127.64, 127.51, 122.06, 121.91, 79.73, 64.95, 53.56, 50.33, 44.65, 44.43, 28.58.

HRMS (DART/Orbitrap, m/z): calcd for [C29H35N2O5]^+^ (M+H)^+^, 491.2541; found, 491.2540.

**Compound 12.** A flame-dried round bottom flask equipped with a stir bar was charged with **11** (0.111 g, 0.226 mmol), MB-chloroformate (0.095g, 0.294 mmol) (Ref : *J. Am. Chem. Soc.* **2022***, 144,* 37, 16799–16807) and 3 mL anhydrous THF. DMAP (35.9 mg, 0.294 mmol) dissolved in anhydrous THF (1 mL) was dropwise added into the mixture. After reaction at 55 °C for 1 day, the mixture was extracted with EtOAc (50 mL). The organic fraction was washed with brine (50 mL), dried over Na_2_SO_4_, filtered and concentrated under reduced pressure to yield a crude mixture. The crude product was purified by column chromatography (50–100% Ether/hexanes) to provide the title compound as a colorless liquid (0.105 g, 58%). R_f_ = 0.15 (hexanes : ether 1 : 1). ^1^H NMR (400 MHz, Chloroform-d) δ 7.56 – 7.15 (m, 18H), 7.15 – 7.04 (m, 2H), 5.29 – 5.14 (m, 2H), 4.75 – 4.35 (m, 4H), 3.58 – 3.25 (m, 4H), 3.00 (s, 12H), 1.54 – 1.39 (m, 9H). ^13^C NMR (100 MHz, Chloroform-d) δ 155.92, 155.14, 154.52, 151.04, 148.95, 148.70, 137.84, 137.63, 133.77, 132.94, 130.05, 129.10, 129.07, 129.05, 128.85, 128.83, 128.74, 128.56, 128.37, 128.25, 128.13, 128.09, 127.53, 127.11, 123.90, 123.86, 121.90, 121.77, 111.23, 110.49, 79.64, 67.16, 50.50, 50.34, 44.73, 44.47, 40.84, 28.58.

HRMS (DART/Orbitrap, m/z): calcd for [C46H52N5O6S]^+^ (M+H)^+^, 802.3633; found, 802.3617.

**TFA salt of SIG2.** A 20 mL vial equipped with a stir bar was charged with **11** (30 mg, 0.037 mmol) and DCM (1 mL) at room temperature. 1 mL TFA was added into the mixture dropwise. After 1 h reaction at room temperature, the product was obtained as a light-blue liquid by removing all solvent under reduced pressure. (>99%, 30 mg). ^1^H NMR (400 MHz, Chloroform-d) δ 7.53 – 7.44 (m, 2H), 7.41 – 7.28 (m, 10H), 7.25 – 7.20 (m, 2H), 7.17 – 7.02 (m, 6H), 5.22 (s, 2H), 4.61 (s, 2H), 4.15 (s, 2H), 3.65 – 3.57 (m, 2H), 3.23 – 3.13 (m, 2H), 3.00 (s, 12H). ^13^C NMR (100 MHz, Chloroform-d) δ 161.46, 161.07, 160.69, 151.12, 142.35, 137.46, 133.95, 130.09, 130.03, 129.65, 129.46, 129.26, 128.74, 128.44, 127.66, 122.19, 118.61, 117.20, 114.33, 87.04, 68.67, 53.57, 51.79, 46.41, 46.19, 45.81, 27.72.

HRMS (DART/Orbitrap, m/z): calcd for [C41H44O4N5S]^+^ (M)^+^, 702.3109; found, 702.3100.

# 8. NMR Spectrum


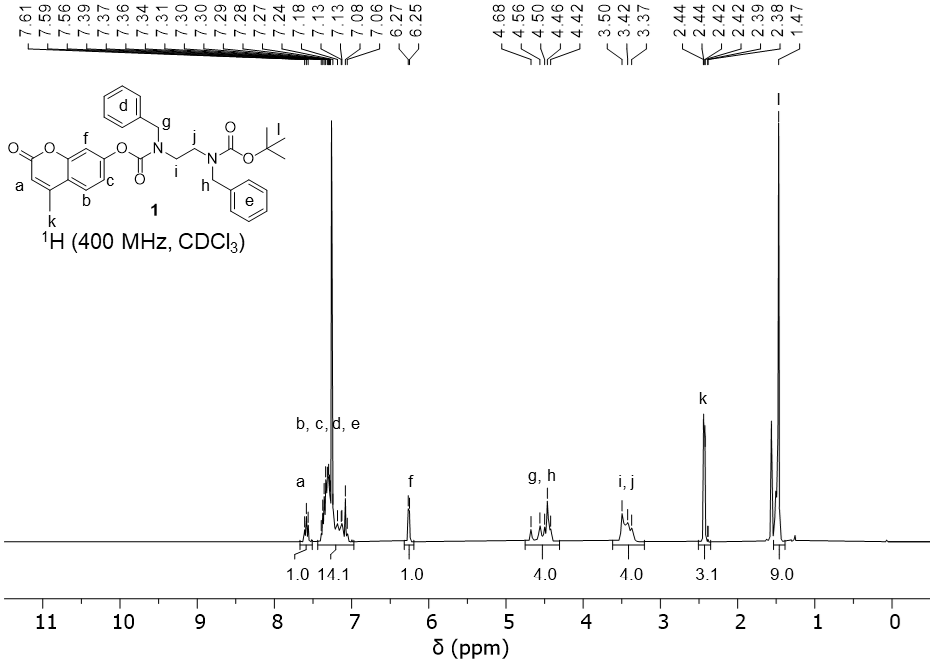


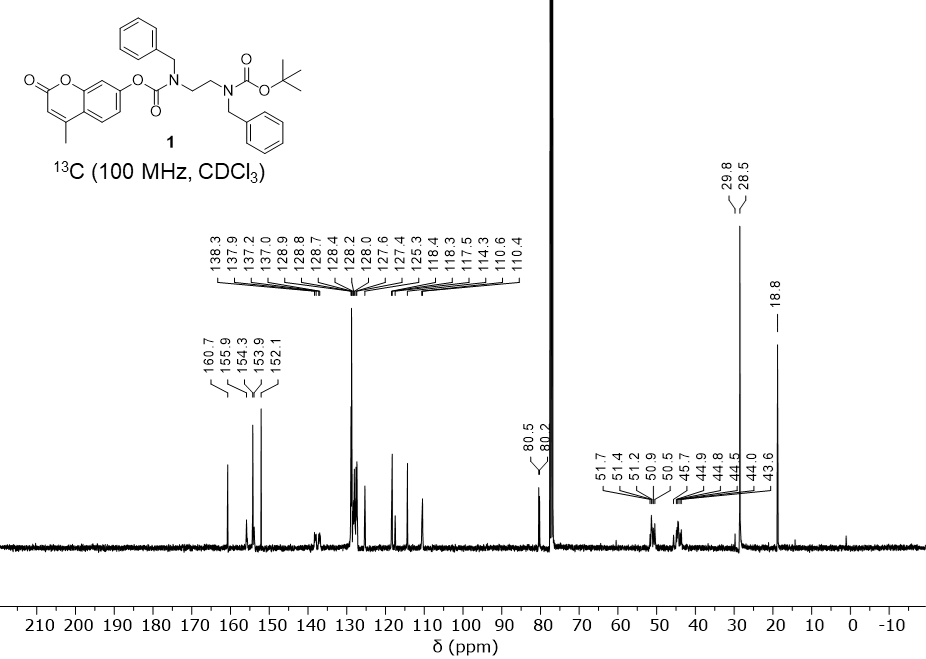


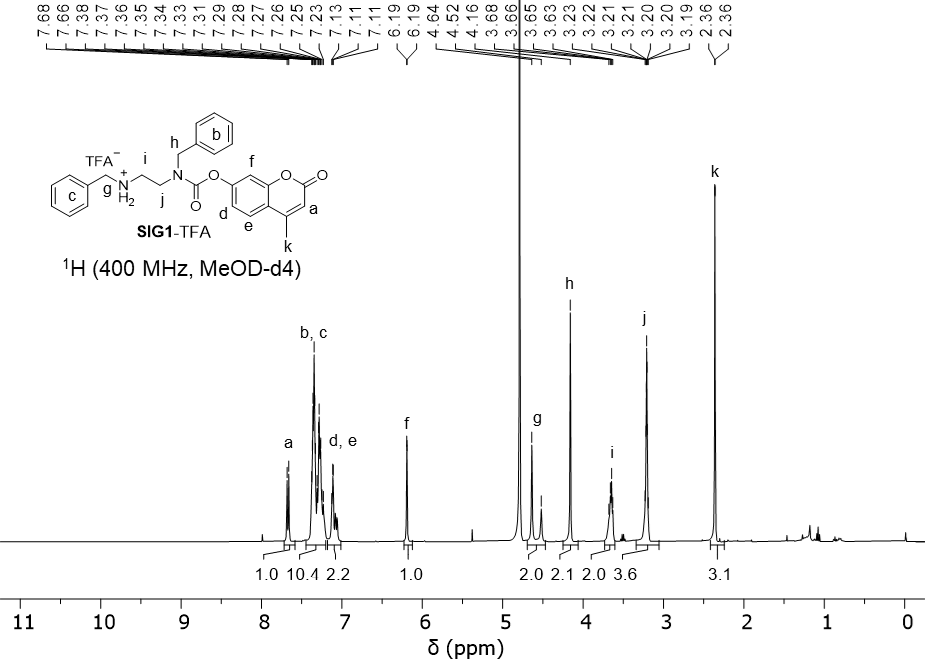


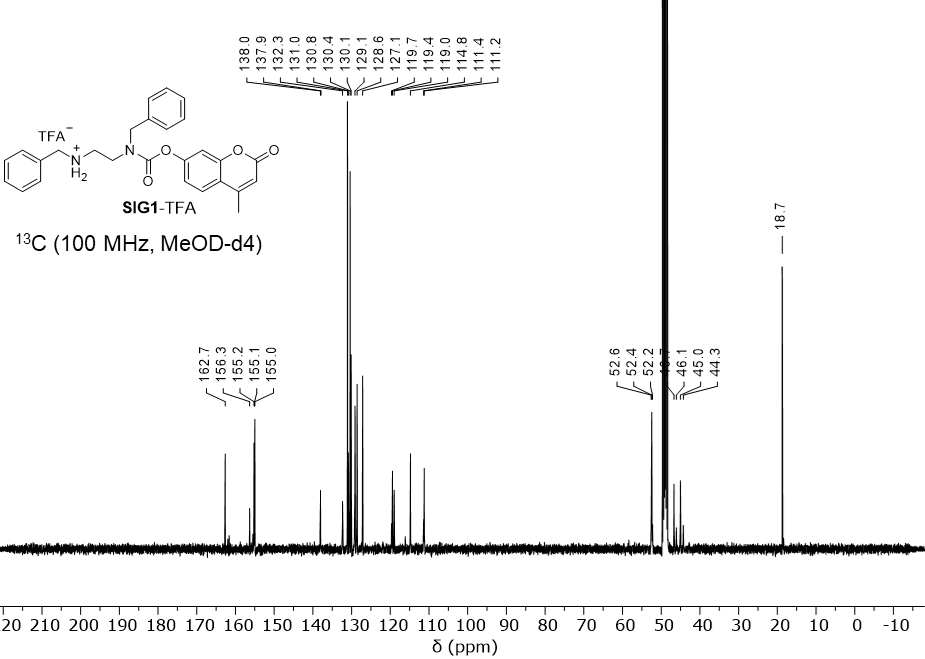


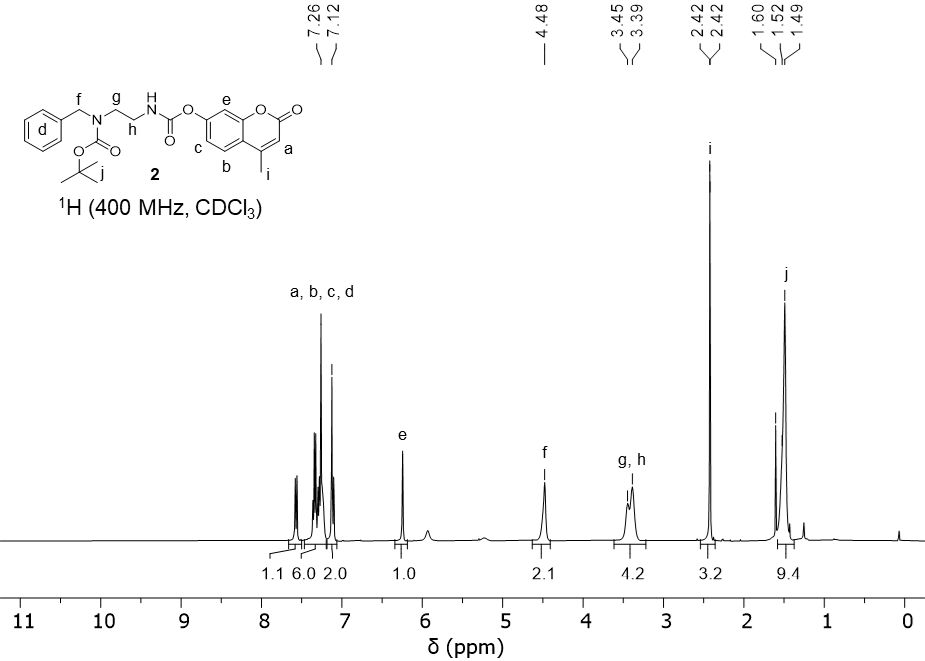


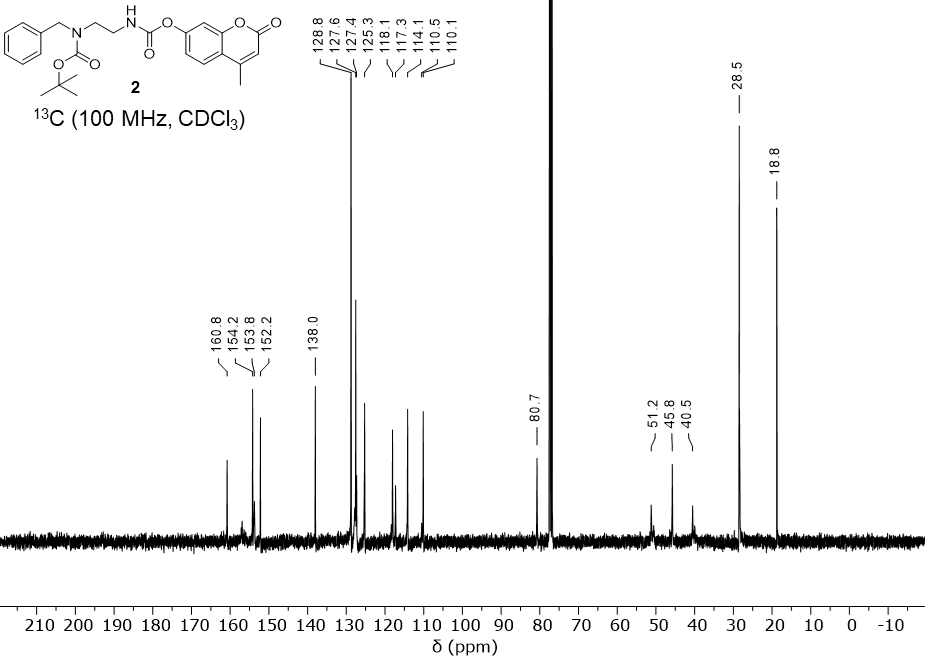


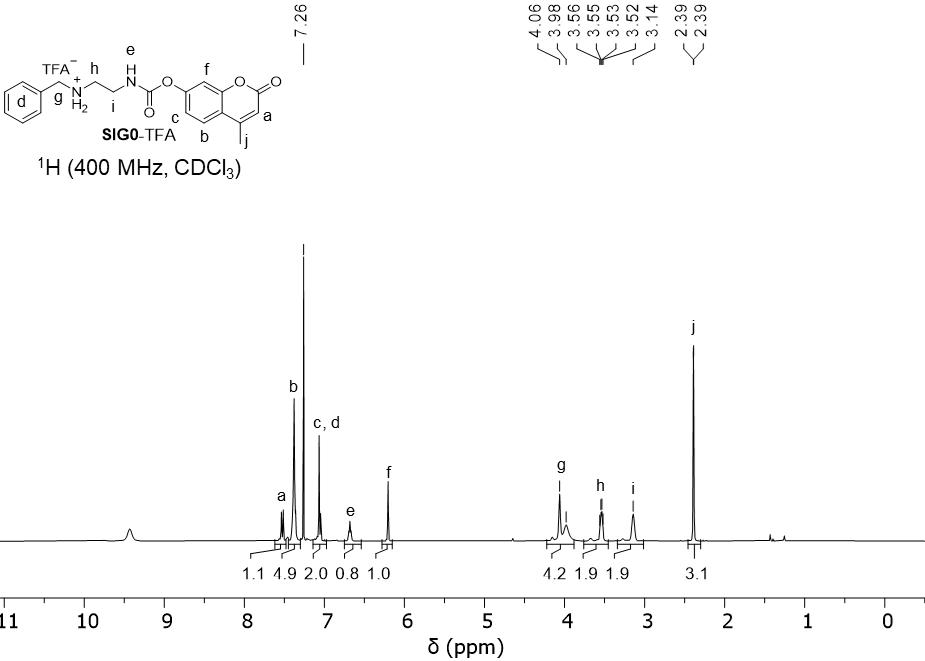


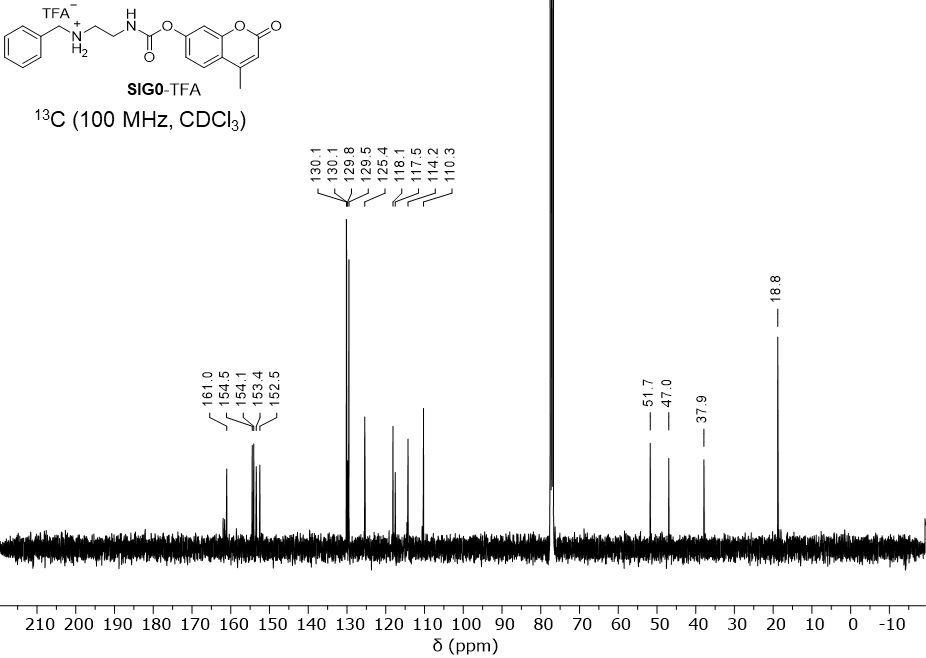


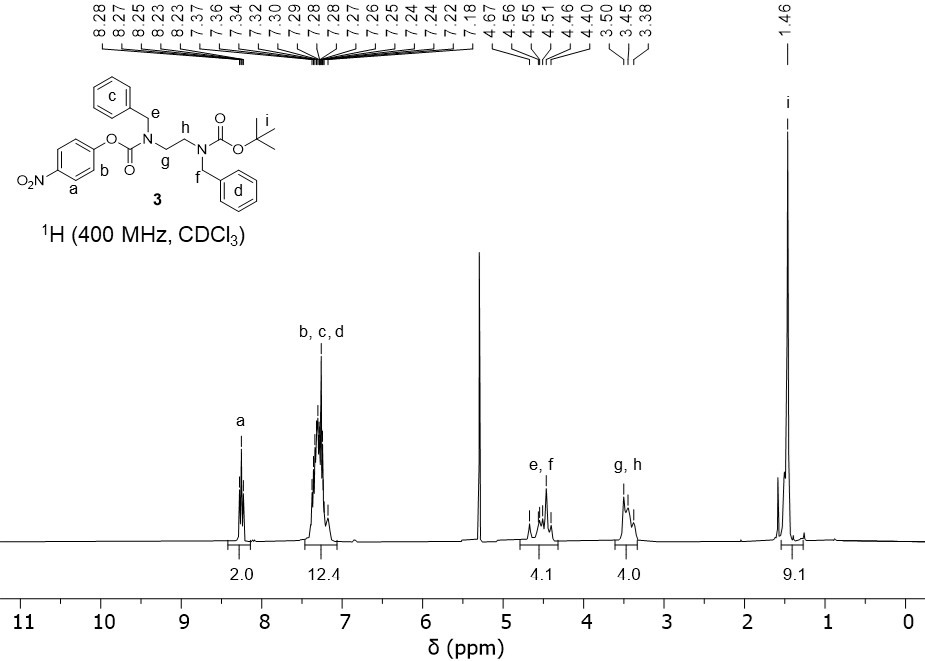


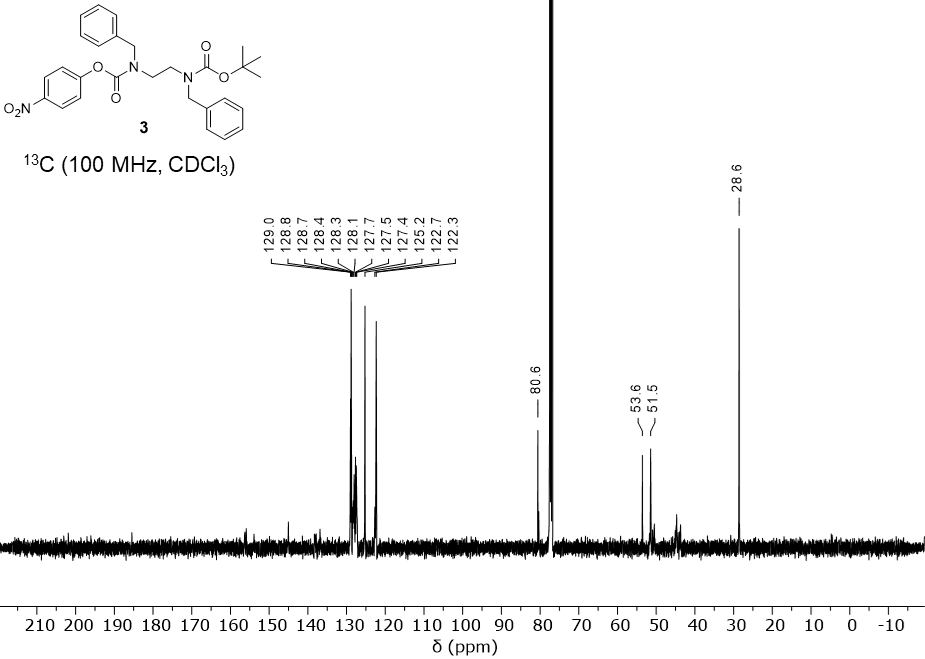


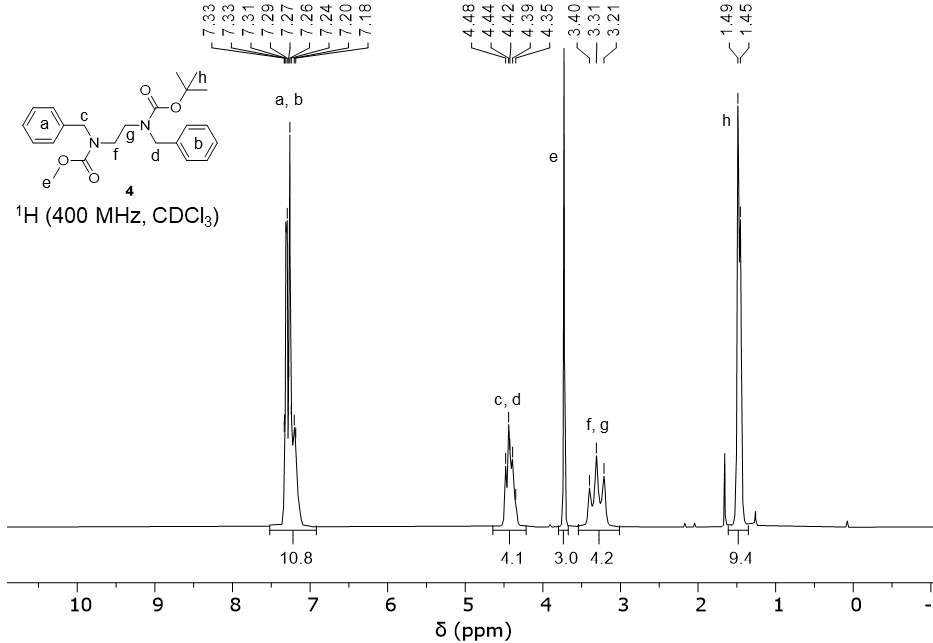


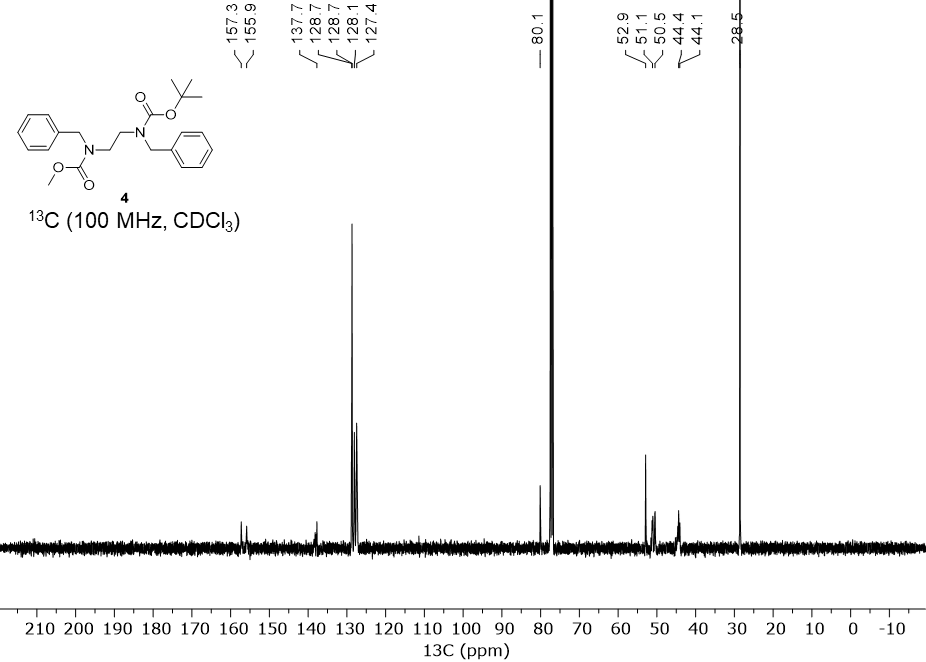


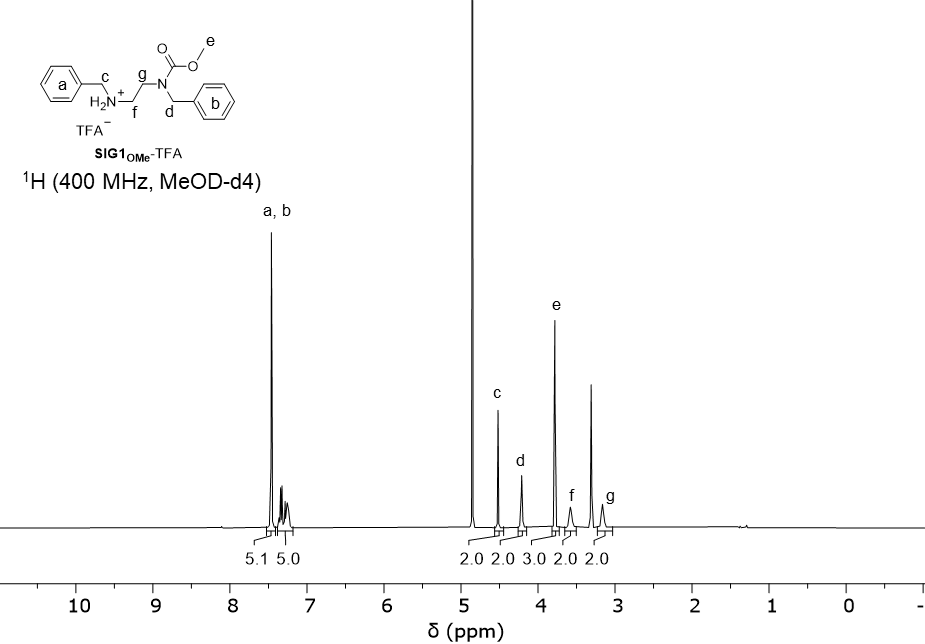


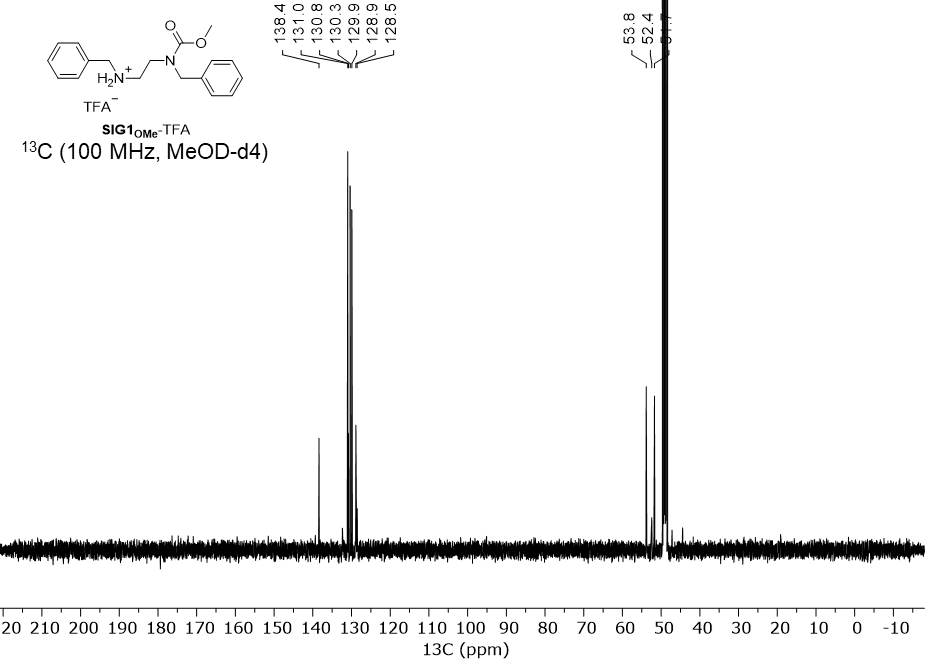


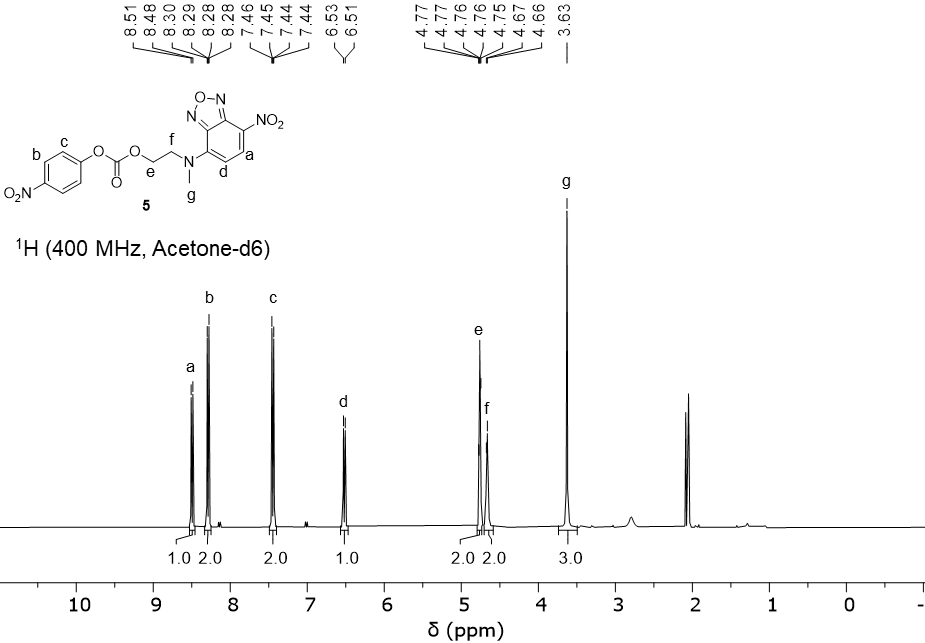


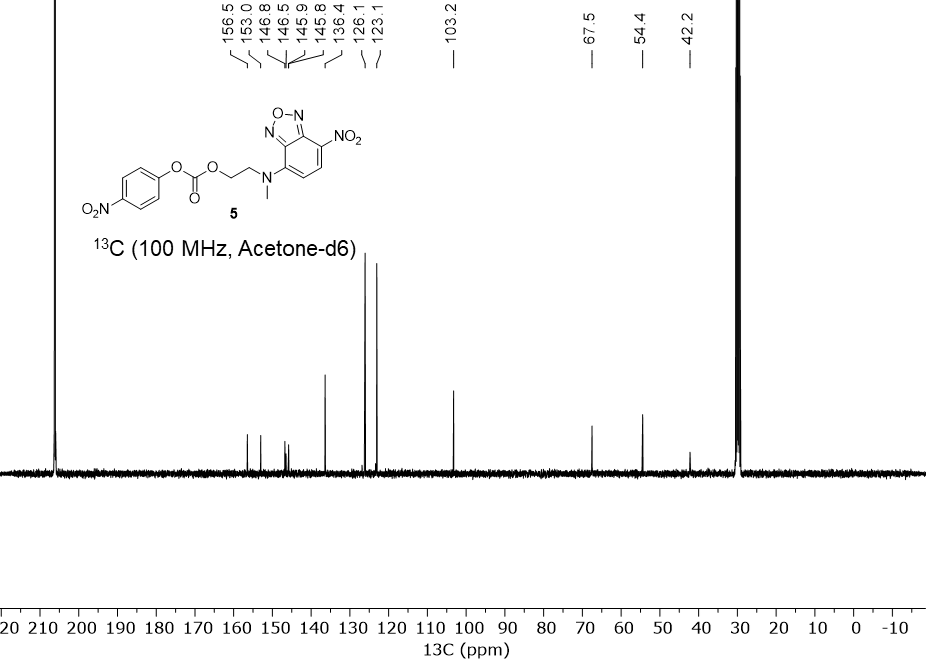


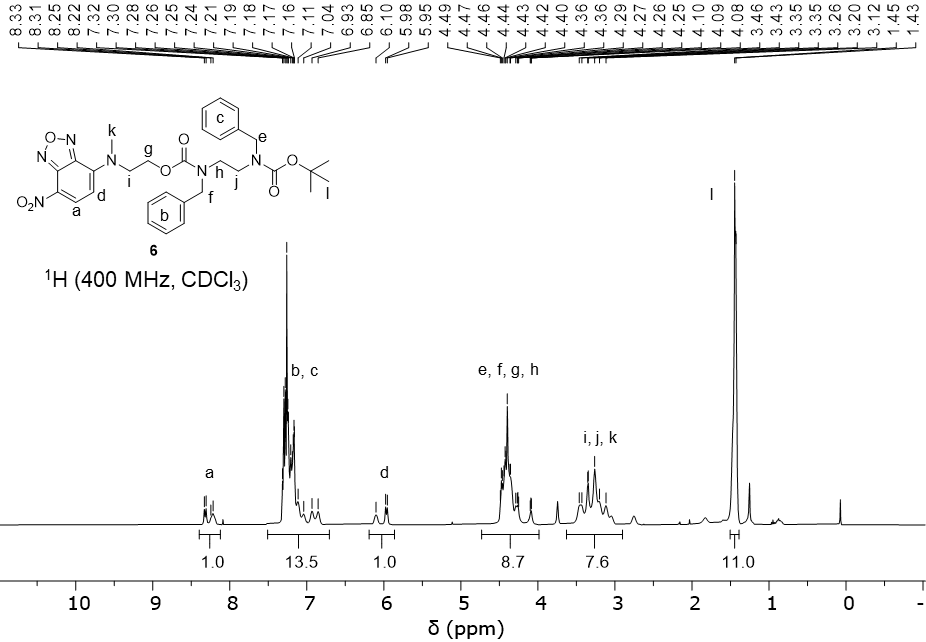


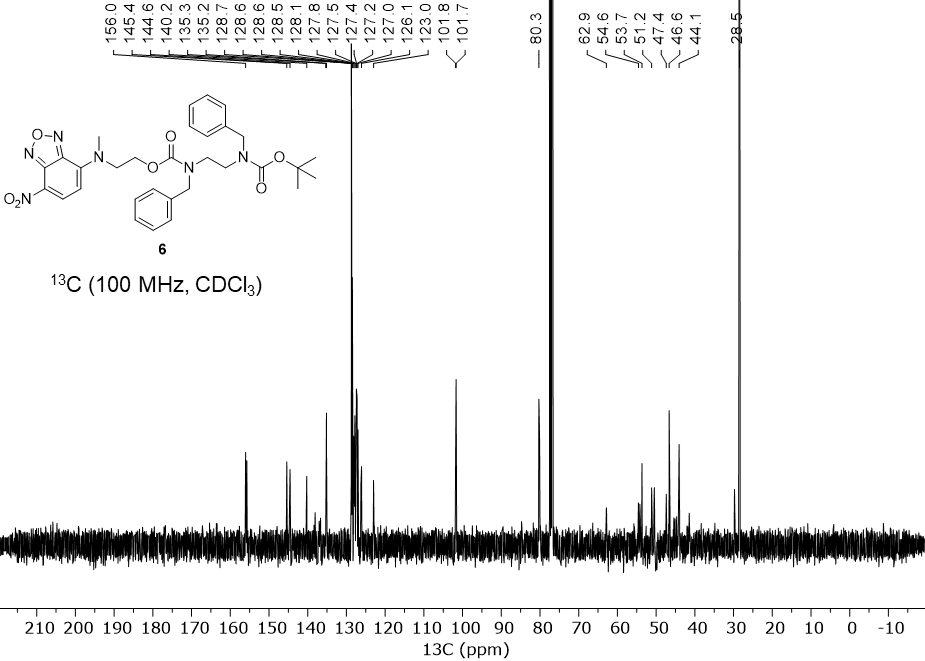


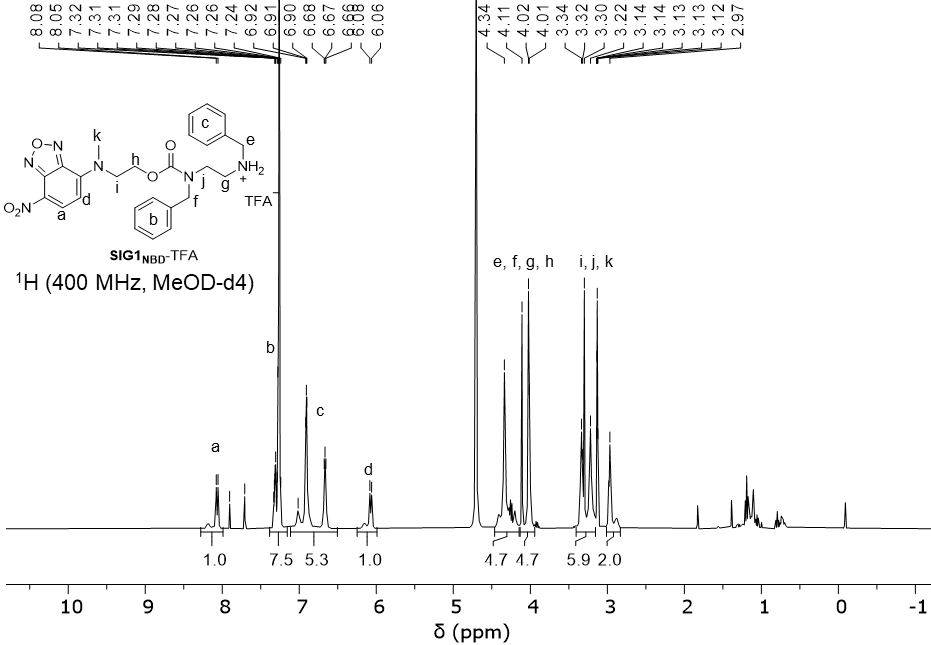


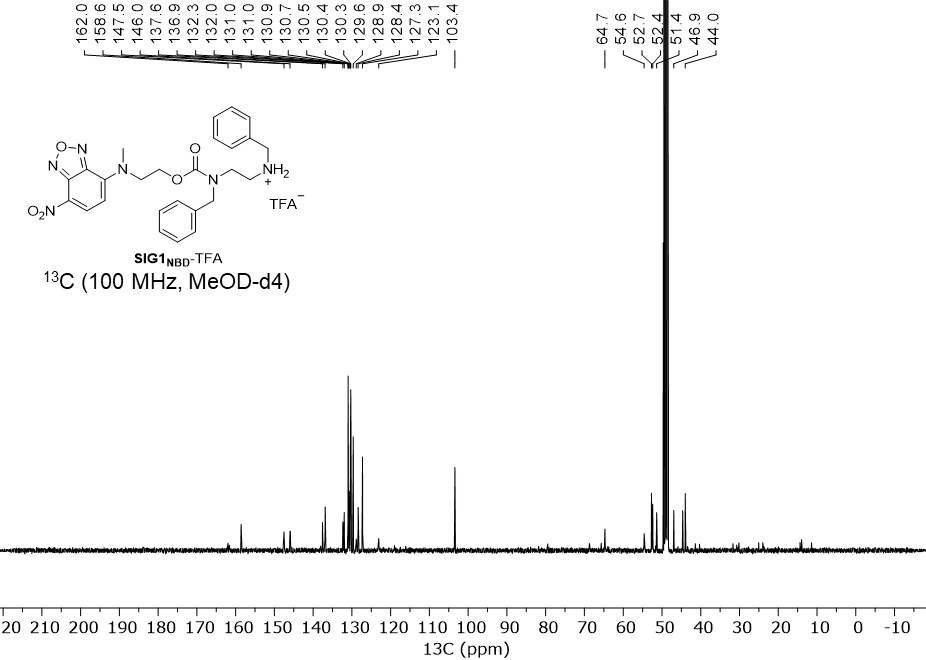


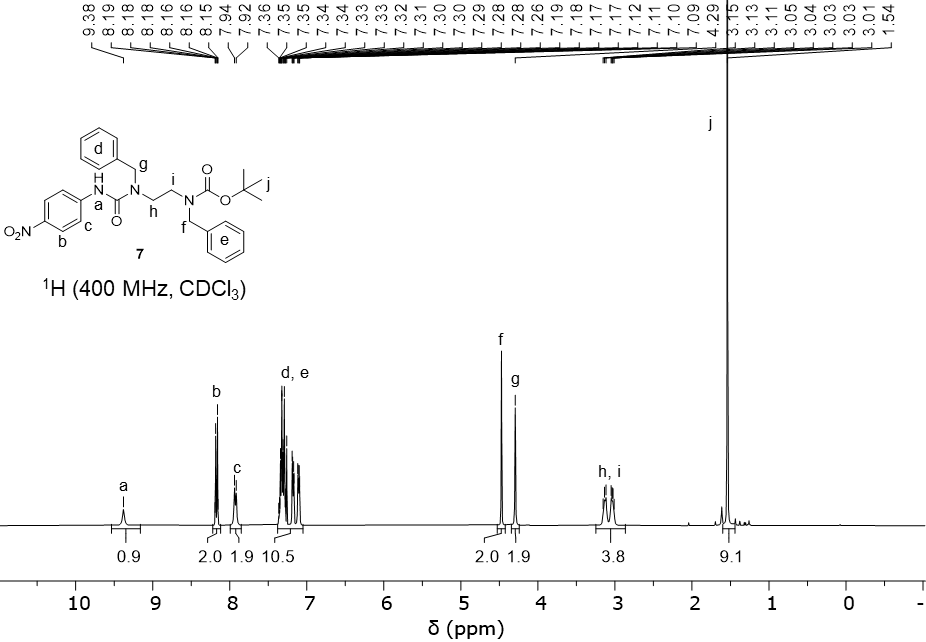


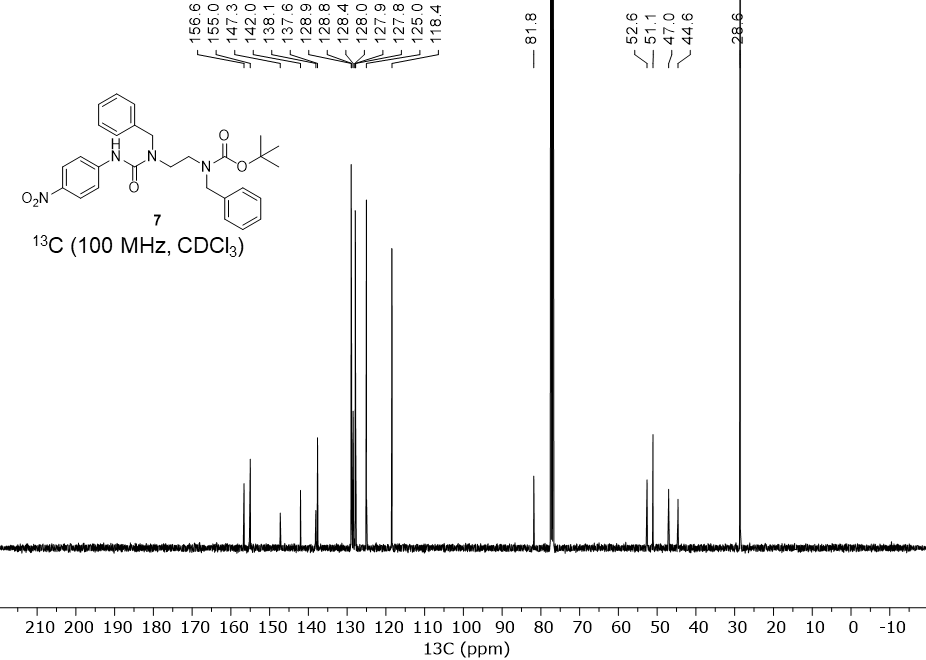


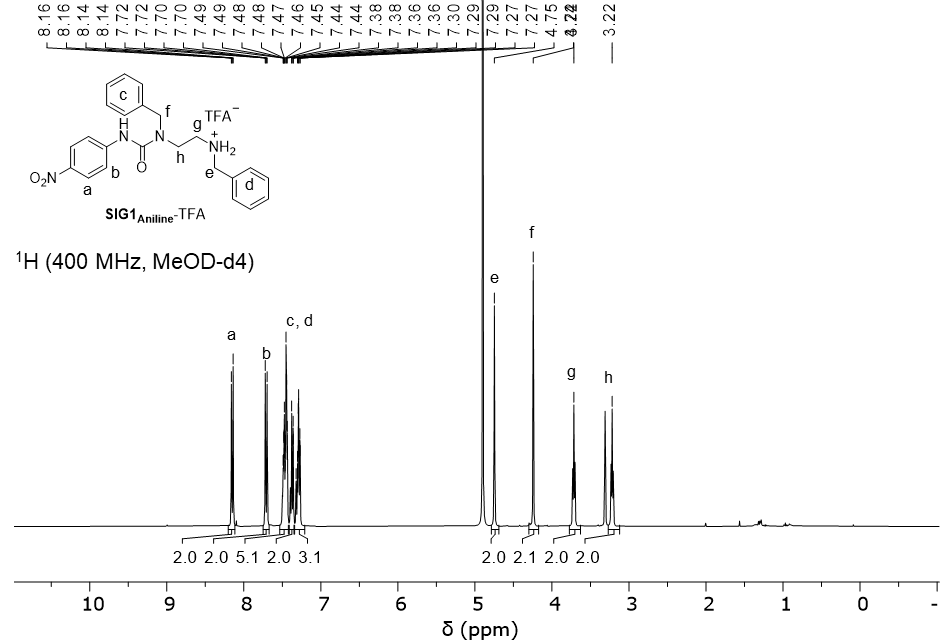


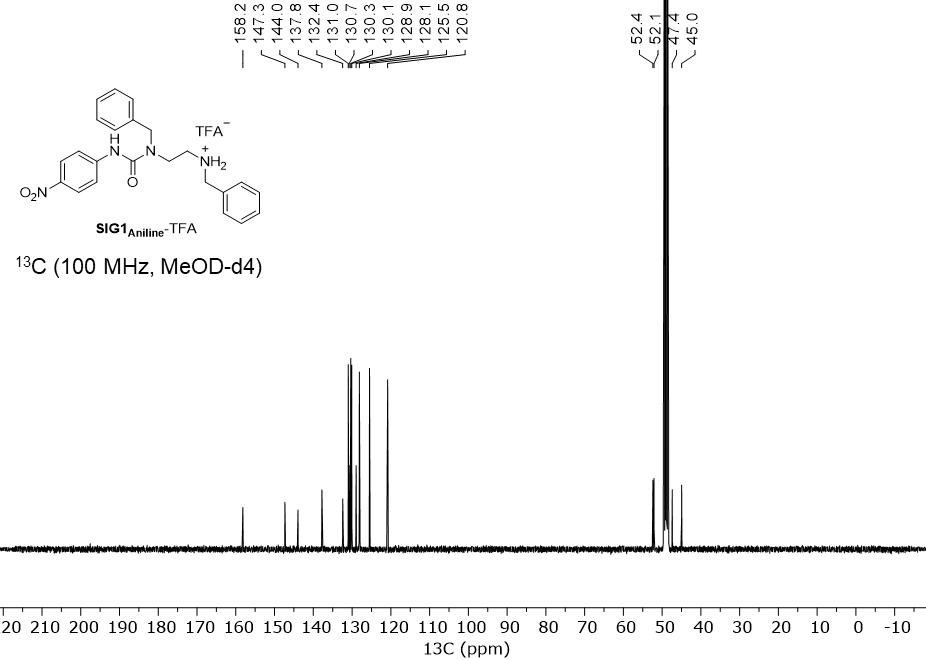


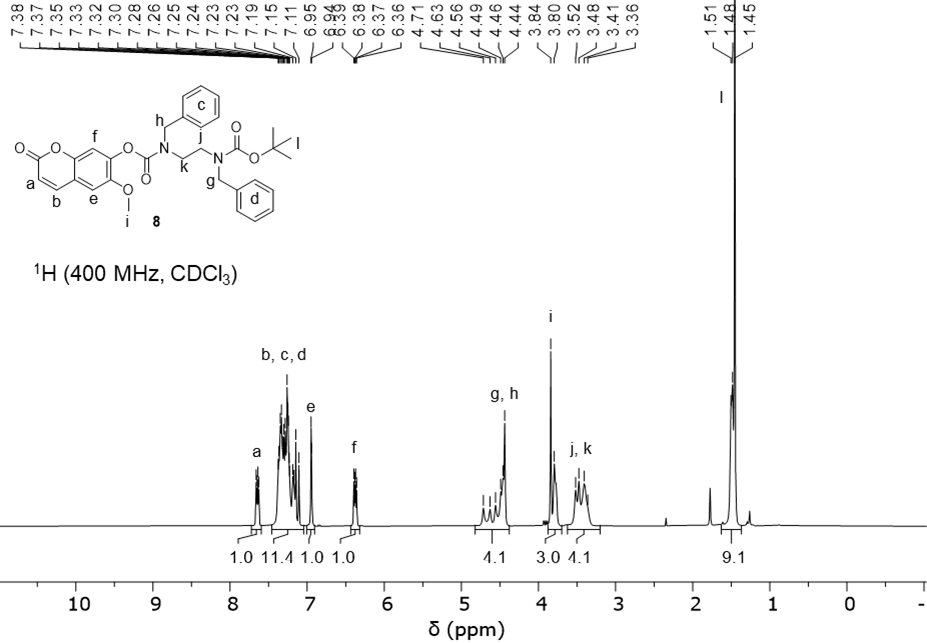


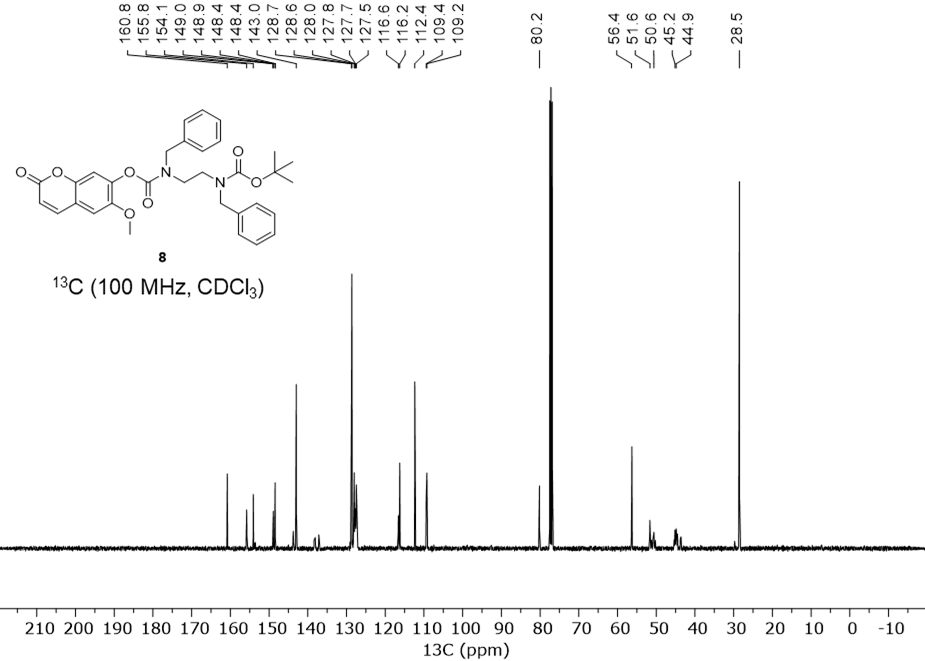


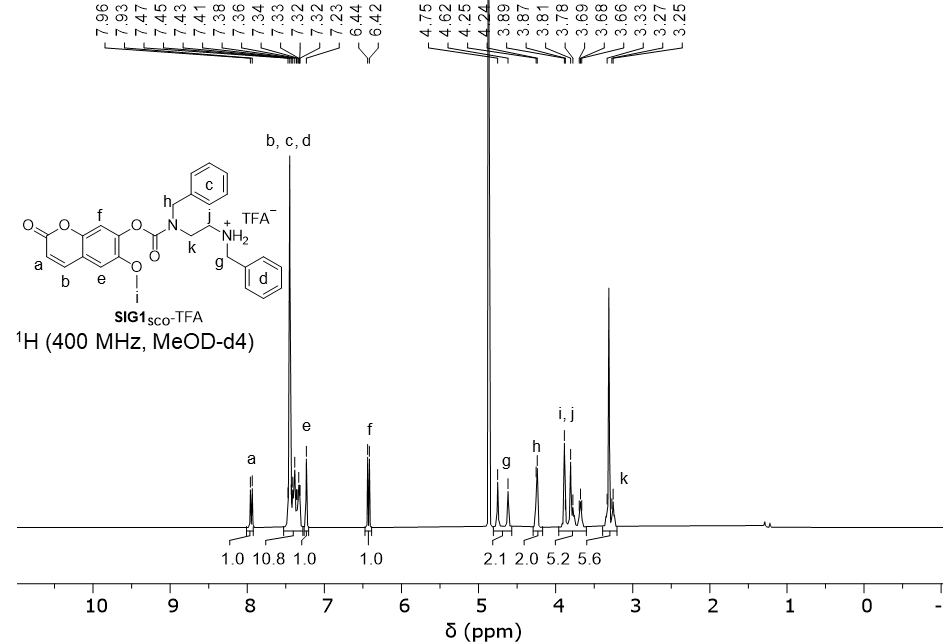


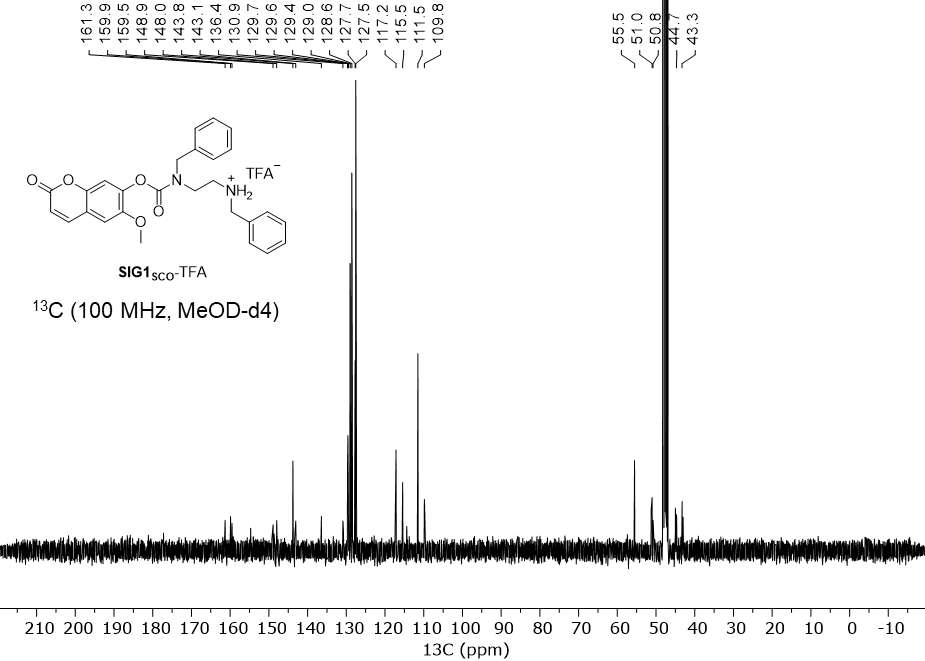


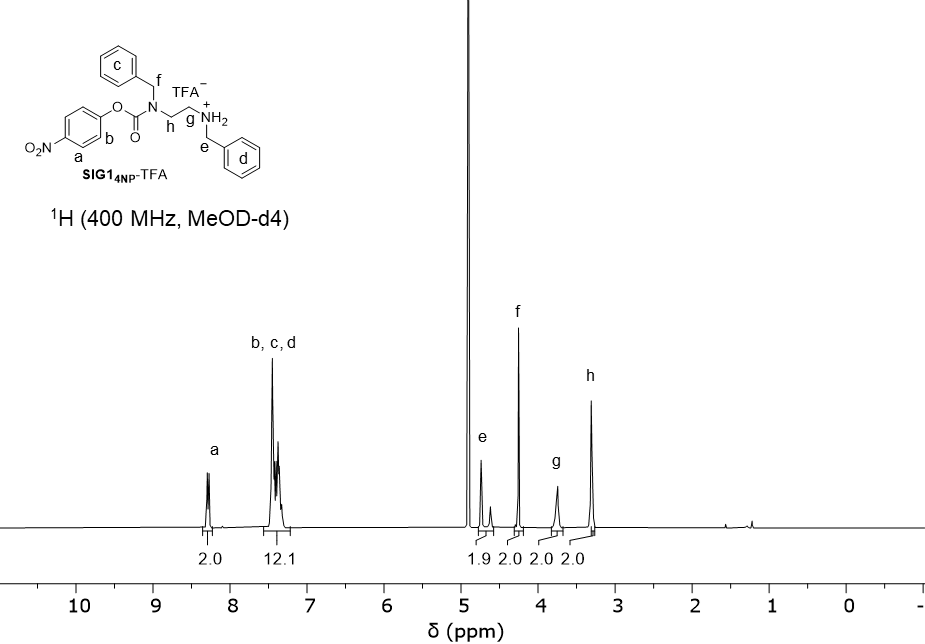

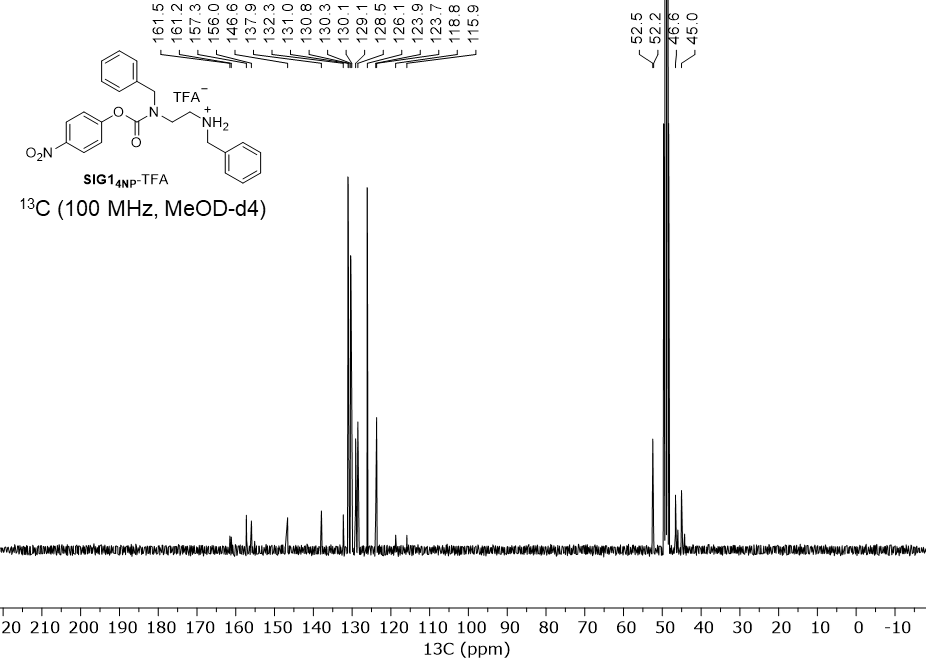


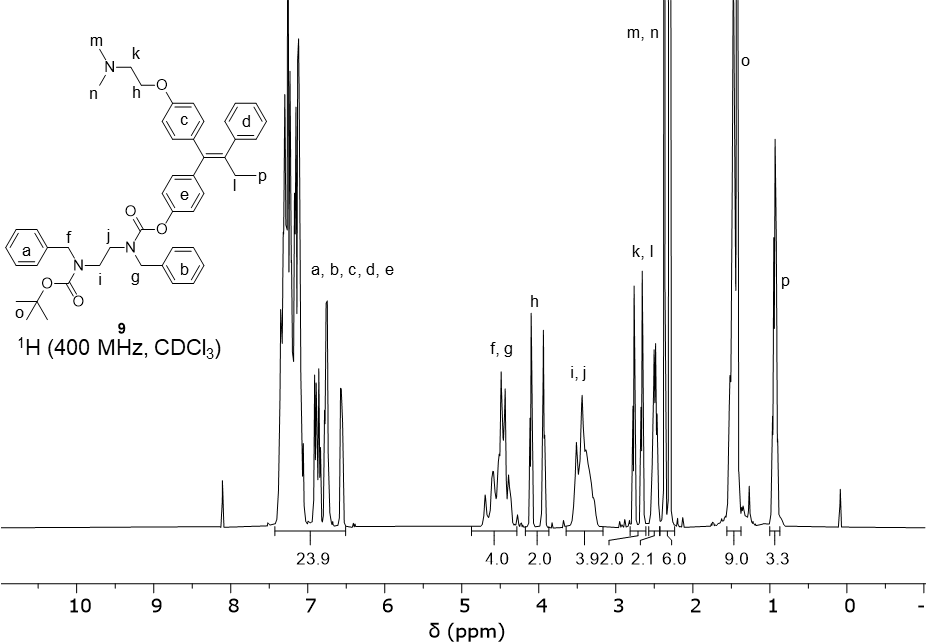


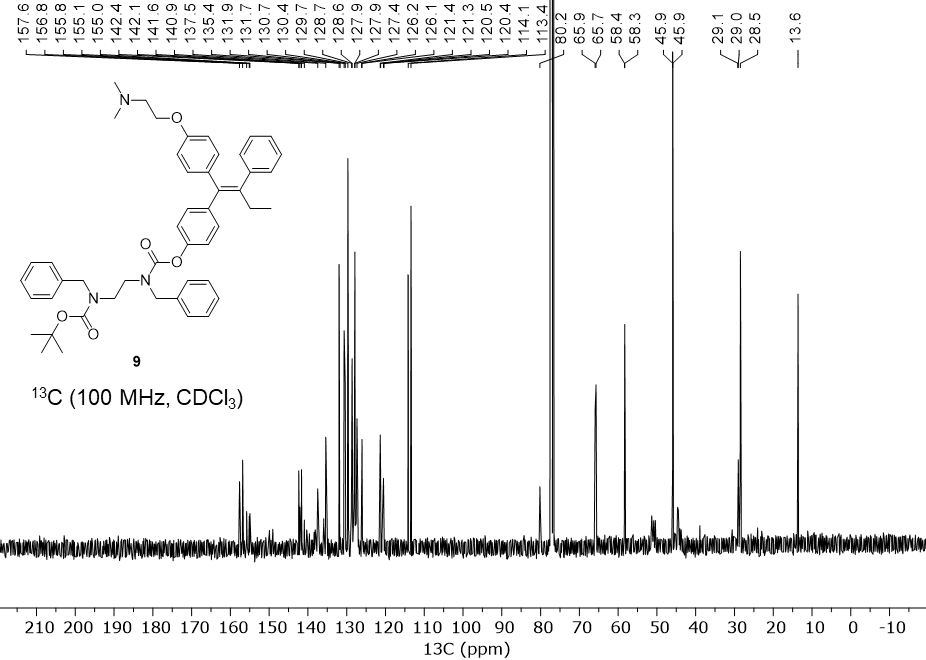


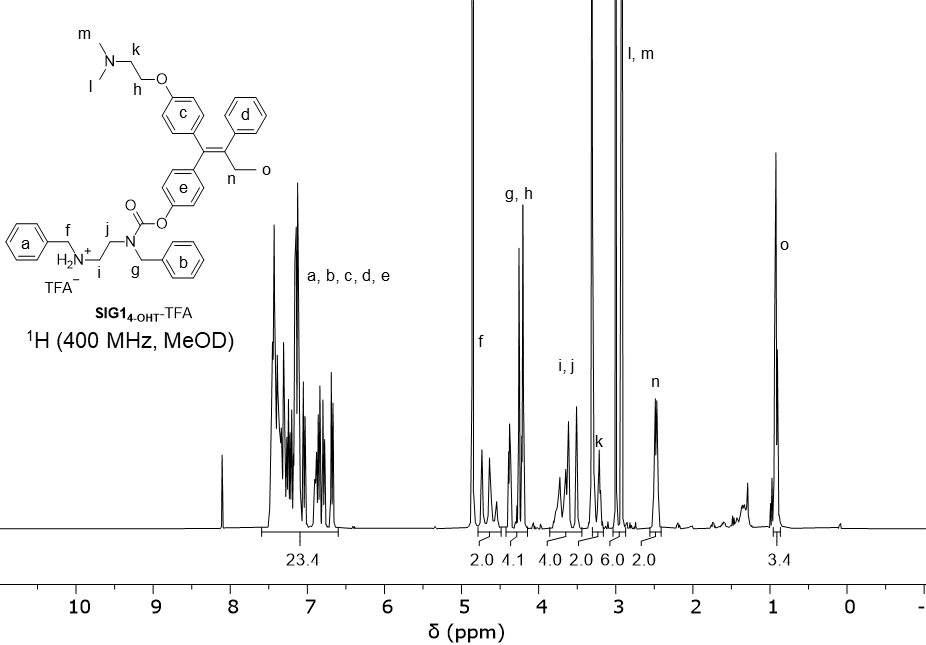


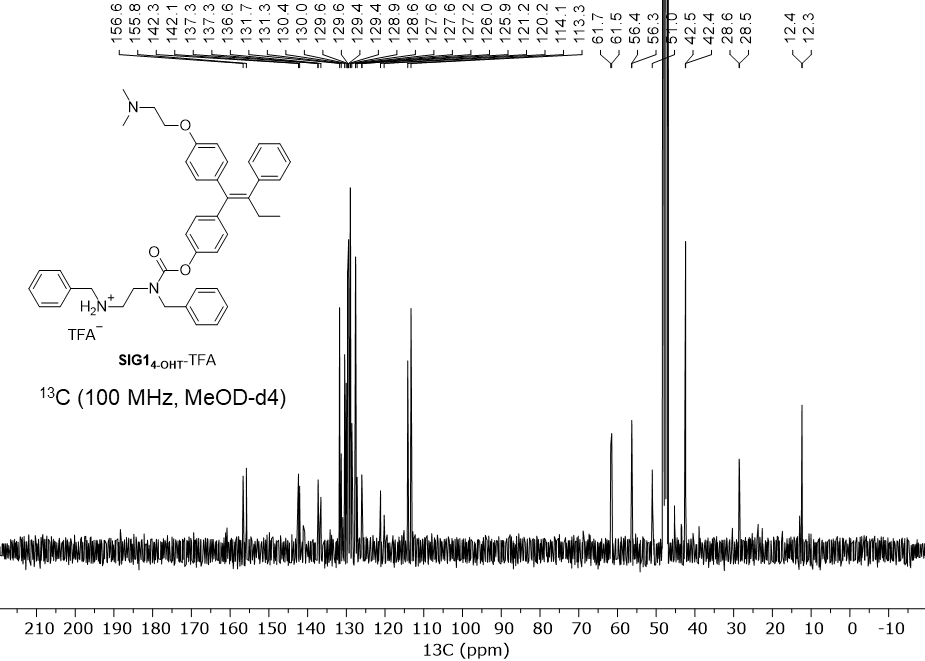


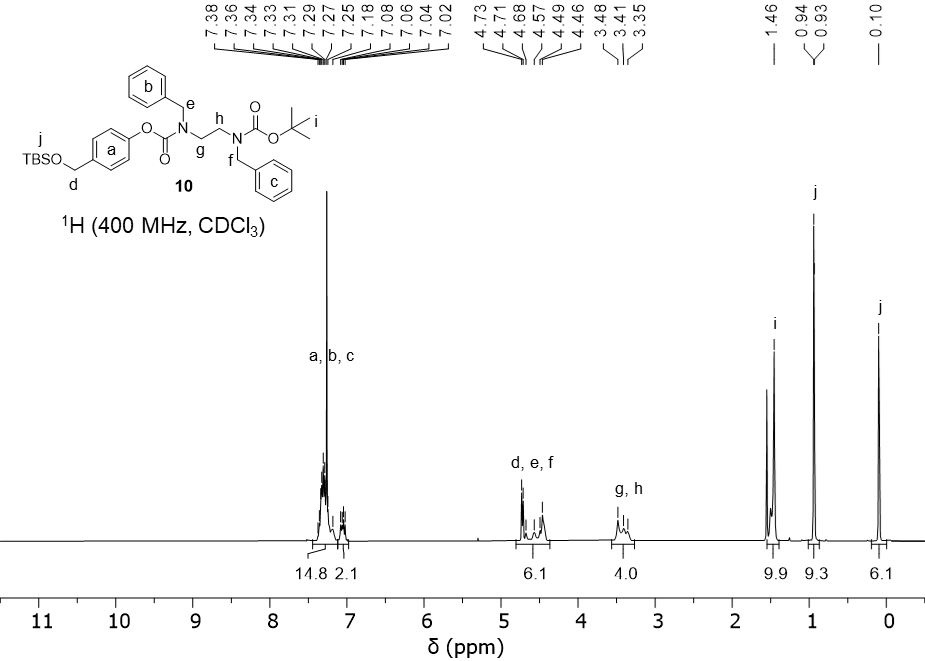


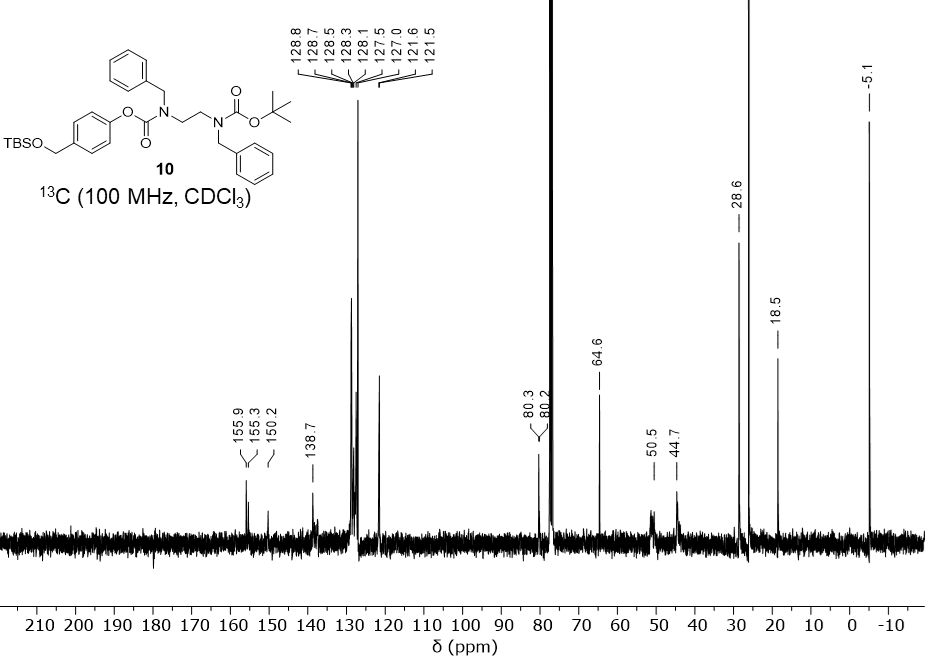


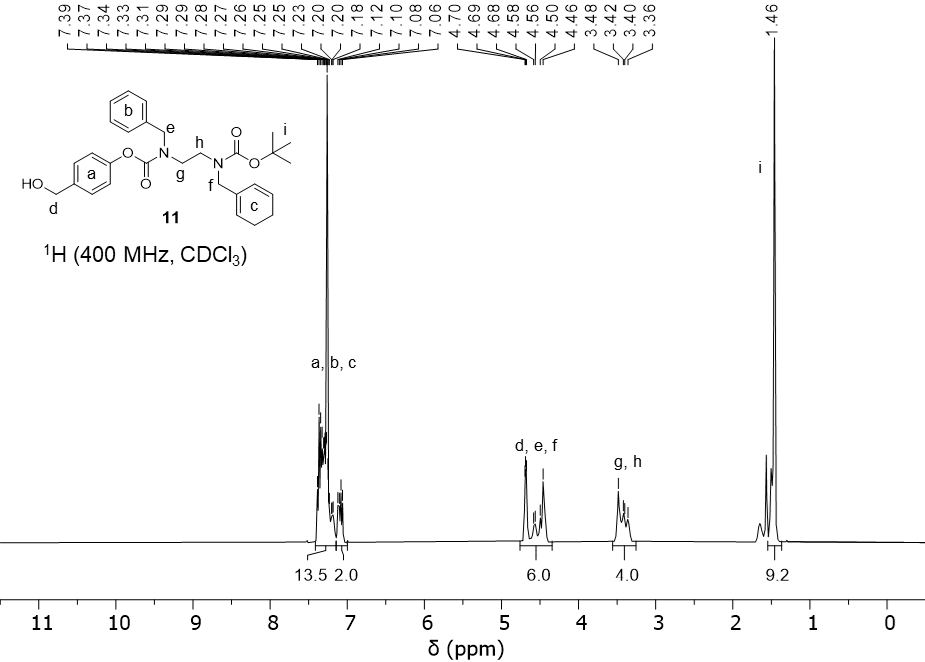


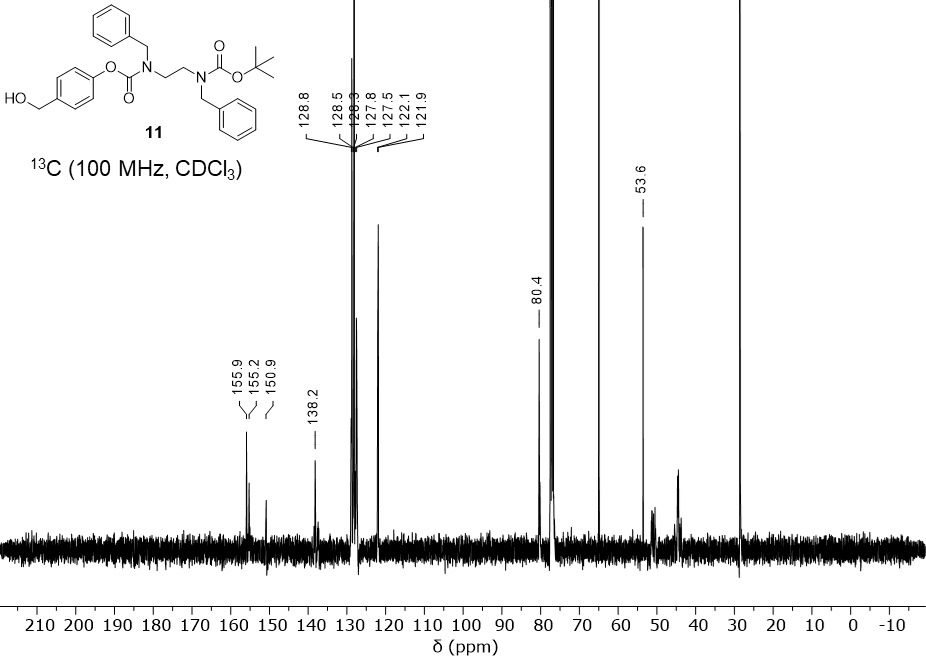


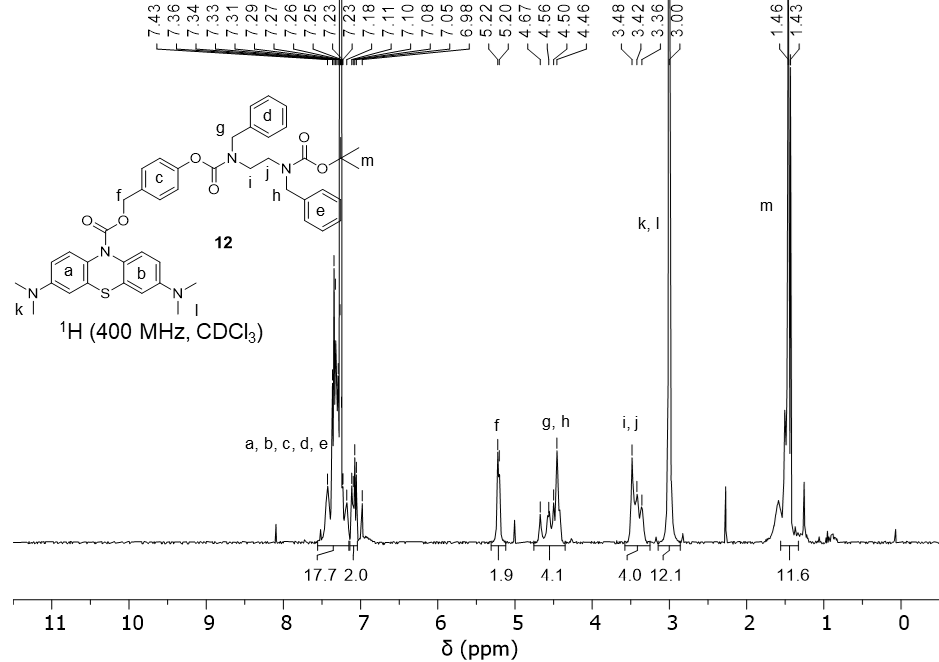


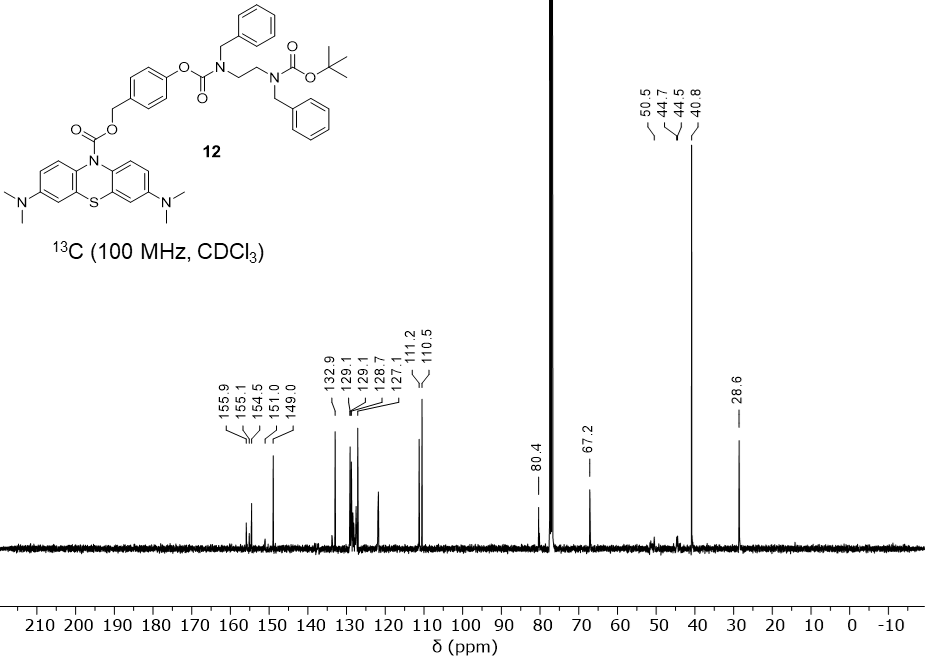


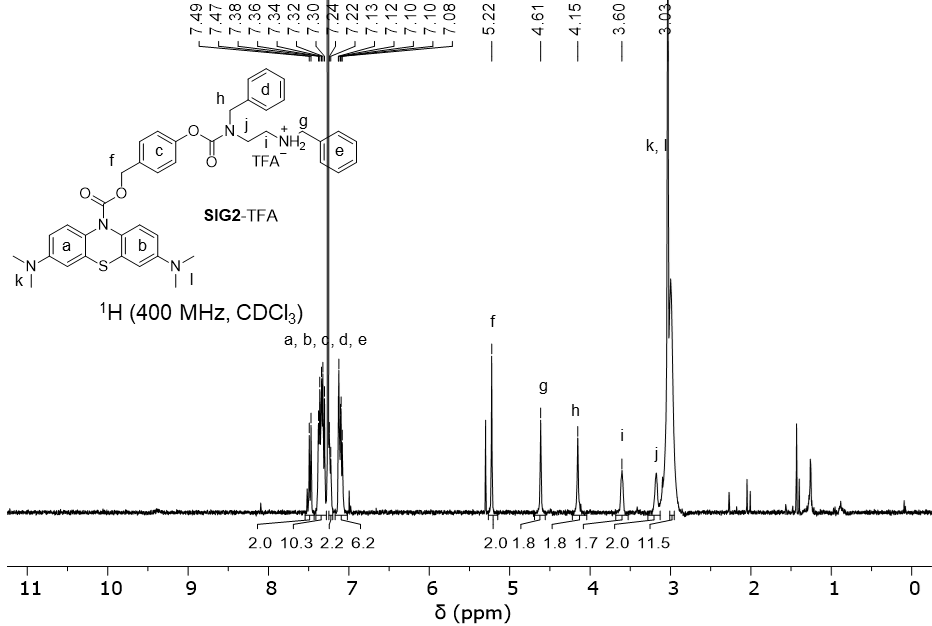


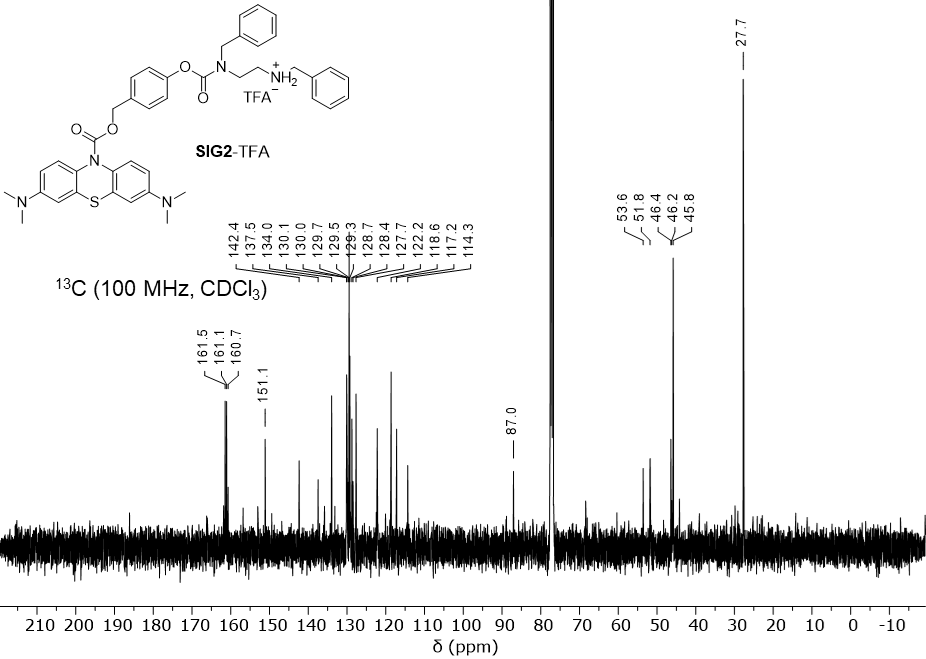

Supplement: Supplementary file 1 — Supporting Information [file ANIE-65-e15594-s001.docx]
